# Smoking cessation in pregnancy: a systematic review and meta-analysis of vulnerability approaches and expectant father involvement

**DOI:** 10.3389/phrs.2026.1609825

**Published:** 2026-09-14

**Authors:** Bibiana Barrera, Furqan Ahmed, Tilman Brand, Hajo Zeeb

**Affiliations:** 1 Faculty 11 Human and Health Sciences, University of Bremen, Bremen, Germany; 2 Department of Prevention and Evaluation, Leibniz Institute for Prevention Research and Epidemiology - BIPS, Bremen, Germany

**Keywords:** behavioral interventions, father involvement, meta-analysis, pregnancy, pregnant women, smoking cessation, vulnerable populations, second-hand smoke

## Abstract

**Objectives:**

To evaluate behavioral and psychosocial smoking cessation interventions and second-hand smoke reduction strategies in pregnancy, focusing on sociocultural vulnerability approaches and expectant father involvement.

**Methods:**

Systematic review and meta-analysis (Joanna Briggs Institute methodology; PRISMA 2020). MEDLINE, Scopus, PsycINFO, ASSIA, and CINAHL were searched to October 2023; exclusively pharmacological interventions were excluded. Random-effects meta-analyses examined five vulnerability approaches and two levels of father involvement. Protocol: https://doi.org/10.17605/OSF.IO/DUYF7.

**Results:**

Fifty-eight studies (41 RCTs) were included. Individual counseling was most effective for maternal cessation (OR = 1.66, 95% CI: 1.39–1.99). Vulnerability-adapted and standard interventions were comparably effective overall (OR = 1.55 vs. 1.51); socioeconomic inclusiveness yielded the largest subgroup effect (OR = 2.51), although subgroup estimates were exploratory (low to very low certainty). Father involvement did not significantly improve maternal cessation (OR = 1.12), but father-directed interventions were highly effective for paternal cessation (OR = 2.18; high-certainty evidence).

**Conclusion:**

Behavioral interventions, particularly individual counseling, are effective in pregnancy. Father-directed approaches merit consideration in antenatal care, although evidence derives from four trials. Socioeconomically inclusive and mental health-integrated interventions are promising but require confirmation.

**Systematic Review Registration:**

https://doi.org/10.17605/OSF.IO/DUYF7, identifier 10.17605/OSF.IO/DUYF7.

## Introduction

Tobacco use is a major cause of preventable morbidity and mortality worldwide, with smoking during pregnancy posing serious risks to both mother and child [1]. Adverse perinatal outcomes causally associated with maternal smoking include ectopic pregnancy, placental abruption, miscarriage, preterm birth, low birth weight, and congenital anomalies [2–5]. Many harmful substances in tobacco smoke cross the placenta, including carbon monoxide—which impairs fetal oxygen supply—and carcinogens such as polycyclic aromatic hydrocarbons and tobacco-specific nitrosamines [4]. Long-term consequences for children exposed prenatally include respiratory conditions, including asthma and wheezing, as well as neurodevelopmental and psychological challenges such as attention-deficit/hyperactivity disorder [6, 7]. After birth, second-hand smoke (SHS) exposure is causally linked to sudden infant death syndrome, middle ear disease, and lower respiratory tract infections [6, 8, 9]. Exposure to thirdhand smoke—residual nicotine and chemical deposits on indoor surfaces—poses additional risks [10]. Children of smoking parents are also more likely to become smokers themselves, perpetuating intergenerational tobacco dependence [11, 12].

Despite the known risks, smoking cessation during pregnancy remains suboptimal. While approximately 25%–50% of pregnant women who smoke achieve cessation during pregnancy [6, 13], a substantial proportion do not, and this proportion is markedly higher among those experiencing socioeconomic disadvantage [14]. Factors associated with continued smoking during pregnancy include low educational attainment, poor mental health, high stress levels, limited social support, and membership of a household where a partner also smokes [13]. Inequalities in smoking prevalence are glaring: in Europe, the prevalence of smoking during pregnancy is approximately five times higher among women with lower educational attainment compared with their more highly educated counterparts [4]. These health inequalities establish early pathways for tobacco-associated disease burden in already disadvantaged populations [15]. Interventions to support smoking cessation during pregnancy exist across a range of formats, but a recent systematic review concluded that women of low socioeconomic status may benefit less from standard cessation interventions than those with higher socioeconomic status, and require more intensive, targeted approaches [16]. In this review, vulnerability is defined as an elevated likelihood of continued smoking during pregnancy and of reduced access to, or benefit from, cessation support, arising from intersecting social, economic, cultural, and psychological disadvantages, including low socioeconomic status, limited literacy, minority ethnic or migrant background, mental health difficulties, and limited social support [13, 14, 16]. This definition underpins the analytical framework applied throughout the review.

Social networks and household dynamics are pivotal determinants of cessation success. Partner smoking is one of the strongest predictors of continued smoking during pregnancy and of relapse after quitting [17]. In families with traditional gender roles, negotiating smoke-free environments and receiving genuine cessation support from a partner can be especially difficult for women [13, 17]. The transition to parenthood represents a motivationally distinct window of opportunity for expectant fathers to quit smoking, as many are motivated to protect the health of their partner and child [17]. However, there is limited evidence on effective approaches to harness this motivation, particularly regarding how father-directed cessation interventions compare with father-involving couple-based approaches, and how fathers’ gender-specific attitudes to quitting influence their engagement with cessation support [18–20].

Existing systematic reviews, including the Cochrane review by Chamberlain et al. [21], have established that psychosocial interventions are broadly effective for smoking cessation during pregnancy, but have not applied a systematic analytical framework to characterize vulnerability-adapted approaches or the comparative effectiveness of father-involving versus father-directed interventions. The heterogeneity of intervention designs, populations, and outcome measures in the existing literature makes it difficult to identify which specific components drive effectiveness in diverse populations [21, 22].

This systematic review and meta-analysis evaluated the effectiveness of behavioral and psychosocial smoking-cessation interventions and SHS-reduction strategies during pregnancy, using an analytical framework that specifically examined sociocultural diversity, vulnerability, and father involvement—dimensions underrepresented in prior reviews. Specific objectives were to: (a) identify effective intervention delivery formats and strategies for maternal smoking cessation; (b) characterize and evaluate approaches used to address sociocultural vulnerability in pregnant women and their families; and (c) examine the effectiveness of different levels of expectant father involvement, including father-directed cessation interventions.

## Methods

### Protocol and reporting

This systematic review and meta-analysis were conducted in accordance with the Joanna Briggs Institute (JBI) methodology for systematic reviews and are reported following the Preferred Reporting Items for Systematic Reviews and Meta-Analyses (PRISMA) 2020 statement [23]. A PRISMA checklist is provided in Supplementary Material 1. The protocol was prospectively registered on the Open Science Framework (https://doi.org/10.17605/OSF.IO/DUYF7). While the review included all eligible controlled trials of smoking cessation interventions in pregnant women, the analytical framework specifically examined how interventions addressed sociocultural diversity, vulnerability, and father involvement—dimensions underrepresented in existing reviews.

### Inclusion criteria (PICOS)

Eligible study populations comprised pregnant women who smoked and/or their smoking partners (expectant fathers), regardless of age, socioeconomic status, educational level, cultural background, or substance use comorbidities. Eligible interventions targeted smoking cessation, smoke-free home environments, or SHS exposure reduction, delivered through behavioral or psychosocial approaches. Studies evaluating exclusively pharmacological interventions (e.g., nicotine replacement therapy, varenicline, bupropion) without a behavioral component were excluded; comprehensive pharmacological reviews are available elsewhere [22]. The comparator was no intervention, usual care, or a less-intensive intervention that did not explicitly target smoking cessation. Studies comparing two active cessation interventions without a usual care or control group were excluded. Primary outcomes were smoking cessation (self-reported or biochemically confirmed) and smoke-free home environments during or after pregnancy. Eligible study designs included RCTs (individual and cluster), non-randomized controlled trials, and before-after studies with a concurrent control group. No language restrictions were applied.

### Search strategy and data sources

Database searches were conducted in October 2023 across five bibliographic databases: MEDLINE, Scopus, PsycINFO, Applied Social Sciences Index and Abstracts (ASSIA), and Cumulative Index to Nursing and Allied Health Literature (CINAHL). Search strategies combined controlled vocabulary (MeSH) and free-text terms related to smoking, tobacco, pregnancy, and relevant intervention types. The search strategy was drafted and further refined through discussion with a specialist librarian. Full search strategies are provided in Supplementary Material 2. One additional reference was identified through screening the citation lists of included articles.

### Study selection

Following the initial database search, 9,045 references were identified. After removing 4,765 duplicates using the Systematic Review Accelerator DeDuplicator, 4,280 unique records were uploaded to ASReview [24], a validated machine-learning-assisted active-learning tool that ranks records by predicted relevance and allows reviewers to focus their effort on the most promising articles first [25–28]. Inclusion criteria were pre-specified in the system prior to screening to ensure the tool’s use was transparent and reproducible. Two independent reviewers screened titles and abstracts in the order ranked by the algorithm. To ensure the robustness of the screening process, the stopping criterion was defined *a priori* and applied conservatively, following established recommendations for active learning–assisted systematic reviews: screening would conclude once 5% of total records (n = 214) had been consecutively labelled as irrelevant by both reviewers. This criterion was met after 830 records (19.4% of the total) had been human-reviewed; the remaining 3,450 lower-priority records were excluded based on the ML ranking without further human review, consistent with validated ASReview protocols [25, 26]. The consecutive-irrelevant-records rule was selected *a priori* because it is among the most widely applied and empirically supported stopping criteria for active-learning screening, and simulation studies across heterogeneous review topics report recall of 95%–100% of relevant records at comparable thresholds [25–28]. Previous validation studies have demonstrated that ASReview achieves high recall of relevant studies while substantially reducing screening burden. In our screening process, all studies ultimately included in the review were identified prior to reaching the stopping threshold, supporting the adequacy of the screening procedure. Consequently, of the 830 records that were human-screened, 638 were excluded at the title and abstract level, leaving 192 records for a second, fully manual title and abstract review; a further 119 were excluded at this stage. The full texts of 73 potentially eligible records from the database searches, together with one record identified through citation searching, were retrieved and assessed against the inclusion criteria (n = 74). Sixteen were excluded with recorded reasons (Supplementary Material 3), leaving 58 studies for inclusion. Discrepancies at all stages were resolved by consensus.

### Data extraction

Two independent reviewers extracted data using a standardized modified JBI data extraction form [29], capturing: study design, sample size, and participant demographics; country and setting; intervention type, delivery format, duration, intensity, provider, and target; comparator description; outcome definitions, measurement timing, and verification method; and main findings. Interventions were additionally coded according to: delivery format (individual counseling, group-based, telephone, home-based, written materials, technology-mediated); approach to addressing sociocultural vulnerability (language and education adaptation, socioeconomic inclusiveness, specialized provider training, mental health and emotional support, and community and cultural involvement); and level of father involvement (active, passive, or not involved). Data extraction was supported by Elicit, an AI-assisted tool that uses natural language processing to organize paper content; all AI-assisted extractions were verified against the original source documents by both reviewers.

### Risk of bias and certainty of evidence

Risk of bias was independently assessed by two reviewers using the Cochrane Risk of Bias 2 (RoB 2) tool for RCTs and the Risk of Bias in Non-randomized Studies of Interventions (ROBINS-I) tool for quasi-experimental designs [30]. RoB 2 domains assessed were: (1) bias from the randomization process; (2) bias due to deviations from intended interventions; (3) bias from missing outcome data; (4) bias in outcome measurement; and (5) bias in selection of reported results. Each study received an overall rating of low risk, some concerns, or high risk. Discrepancies were resolved by consensus; where consensus was not achieved, a third reviewer was consulted. Complete risk of bias assessments are presented in Supplementary Material 4. The certainty of evidence for each meta-analytic comparison was assessed using the GRADE framework [31], considering risk of bias, inconsistency, indirectness, imprecision, and publication bias. Ratings of high, moderate, low, or very low certainty are presented in the GRADE Summary of Findings table (Supplementary Material 8).

### Data synthesis and analysis

Random-effects meta-analyses were conducted in two stages: (1) 50 studies reporting maternal smoking cessation outcomes, within which subgroup analyses examined five vulnerability approaches and two levels of father involvement; and (2) four studies reporting paternal smoking cessation outcomes. Effect sizes are reported as odds ratios (OR) with 95% confidence intervals. Statistical heterogeneity was assessed using Cochran’s Q test and the I^2^ statistic, interpreted as low (0%–30%), moderate (30%–60%), substantial (60%–90%), or considerable (>90%) [32, 33]. Sensitivity analyses were performed by excluding studies with a high risk of bias to assess the robustness of pooled estimates. Forest plots for all primary comparisons and subgroup analyses are presented in Supplementary Material 7. All meta-analyses were conducted using RevMan Web (Cochrane Collaboration) and R (version 4.4.1). Funnel plots were generated for all primary comparisons with ten or more studies; publication bias was assessed by visual inspection and Egger’s regression test (two-tailed α = 0.10; full results in Supplementary Material 7). Four studies measuring SHS exposure only were not pooled due to substantial heterogeneity in outcome measurement methods and are described in a narrative synthesis. Subgroup analyses were pre-specified based on the review’s conceptual framework but should be interpreted with caution given potential residual confounding and heterogeneity across studies.

## Results

### Study selection and characteristics

Database searches identified 9,045 references; one additional record was identified through citation tracking. After removing 4,765 duplicates, 4,280 unique records underwent machine-learning-assisted prioritization. Following full-text review of 74 records, 58 studies met the inclusion criteria (Figure 1). Sixteen full-text records were excluded with reasons documented in Supplementary Material 3. Studies were published between 1985 and 2023; 56 were in English, one in Turkish, and one in Spanish. Studies were conducted across 18 countries, with the majority from high-income settings in North America (n = 30), Northern Europe (n = 14), and Australia/New Zealand (n = 7). Of the 58 included studies, 41 were RCTs; full study characteristics, including design breakdown, country, setting, sample size, and intervention description, are presented in Supplementary Table 5.1 (Supplementary Material 5) [34–65]. The meta-analysis was conducted with 54 studies: 50 reporting maternal smoking cessation outcomes and four reporting paternal cessation outcomes. The remaining four studies, which focused exclusively on SHS exposure, were described narratively. Most interventions were delivered in antenatal care settings by healthcare professionals (midwives, nurses, or physicians) or trained lay counselors; a smaller proportion used telephone or technology-mediated delivery without face-to-face contact.

**FIGURE 1:**
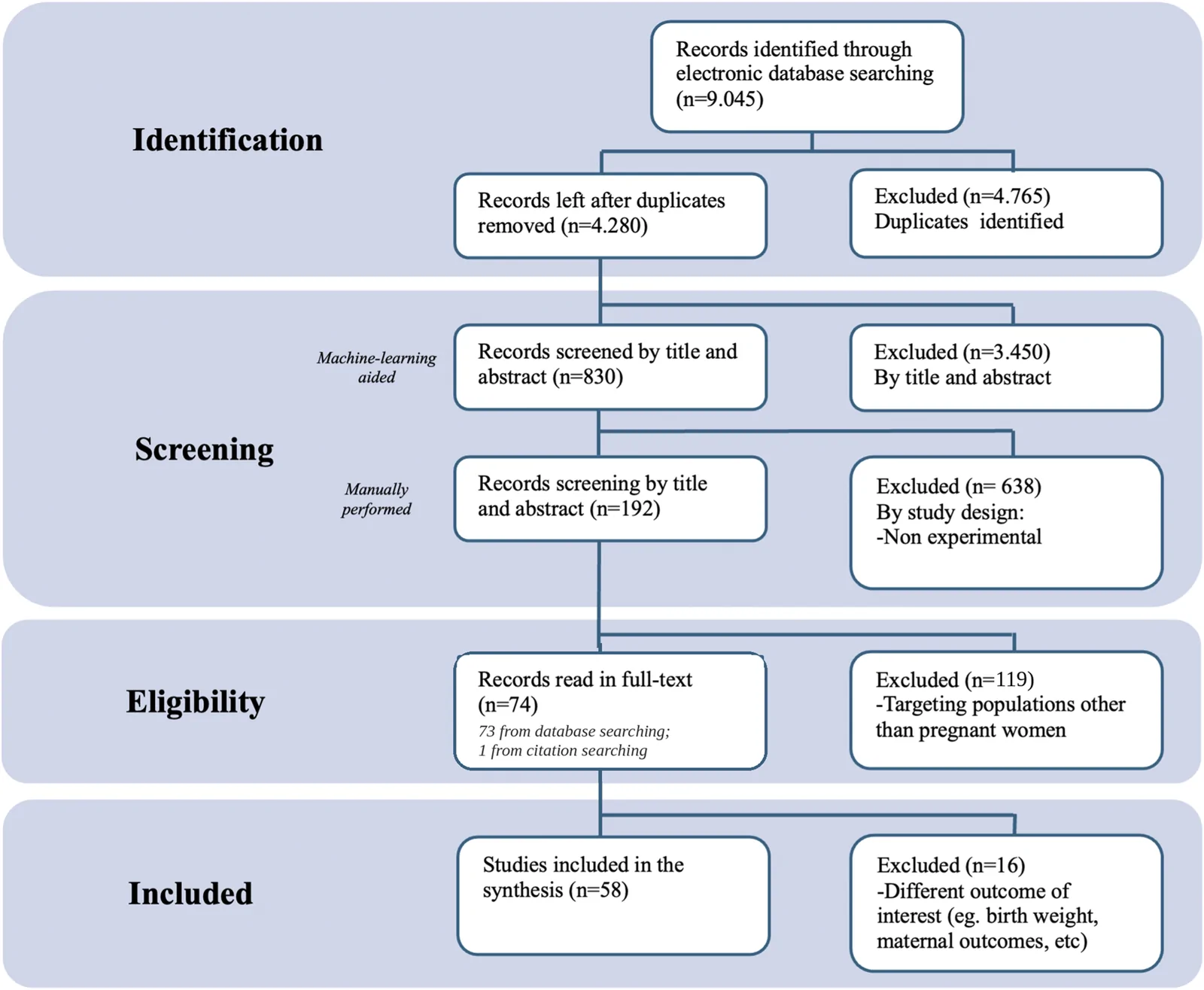
Study selection flow diagram according to the Preferred Reporting Items for Systematic Reviews and Meta-Analyses 2020 statement (multiple countries, 1985–2023).

### Overall intervention effectiveness and delivery formats

The primary meta-analysis of 50 studies confirmed that behavioral and psychosocial smoking cessation interventions are broadly effective during pregnancy across all delivery formats examined, including individual counseling, group-based approaches, telephone-based counseling, home visits, written materials, and technology-mediated interventions. Effect sizes ranged from OR = 1.39 to 1.66 across formats, with individual counseling showing the strongest pooled effect. Consistent with existing Cochrane reviews [21, 66], high heterogeneity across studies reflects the diversity of populations, implementation contexts, and outcome measurement approaches rather than fundamental inconsistency of effect. Full subgroup forest plots by delivery format are presented in Supplementary Material 7. The primary novel contribution of this review—the systematic analysis of vulnerability-adapted approaches and expectant father involvement—is reported in the sections that follow.

### Vulnerability approaches in maternal smoking cessation interventions

Five approaches to addressing sociocultural vulnerability were identified across the included studies. Language and education considerations involved tailoring materials and communication to participants’ linguistic and literacy profiles; for example, Gielen et al. [67] developed educational materials at a sixth-grade reading level for low-income African American women, and Cummins et al. [68] addressed language barriers by providing bilingual counseling in English and Spanish. Socioeconomic inclusiveness involved reducing financial and logistical barriers to participation through financial incentives, practical support, or extended access to resources; Tappin et al. [69] offered up to £400 in financial vouchers to pregnant women from deprived communities in the UK, and Pollak et al. [70] provided cell phones with unlimited texting to low-income Latina participants who lacked reliable access to mobile services. Specialized training for vulnerability involved preparing healthcare professionals and lay counselors to serve individuals with multiple vulnerabilities, including low socioeconomic status, mental health difficulties, domestic abuse exposure, and limited social support; Mejdoubi et al. [71] and Herbert et al. [72] trained staff to engage in non-threatening, culturally responsive communication. Mental health and emotional support integration targeted co-occurring psychological concerns such as depression and anxiety; Bullock et al. [73] provided individualized social support to poor rural pregnant smokers through the Baby BEEP trial, and Dornelas et al. [74] delivered telephone-based mental health counseling to address comorbid conditions alongside cessation. Involvement of local communities and reflecting cultural values involved co-creating or embedding interventions within local cultural contexts; Patten et al. [75] conducted a culturally sensitive cluster RCT for Indigenous women in rural Alaska incorporating trained local ‘Native Sisters’, and Eades et al. [76] co-created an intervention for Aboriginal communities with input from general practitioners, healthcare workers, and community representatives.

At the overall level, interventions reporting any vulnerability approach showed similar pooled effectiveness to those without explicit vulnerability strategies (OR = 1.55, 95% CI: 1.32–1.82; 23 studies; versus OR = 1.51, 95% CI: 1.14–2.00; 27 studies). However, this overall equivalence conceals clinically important differentiation across the five vulnerability approaches. Socioeconomic inclusiveness yielded by far the largest pooled effect (OR = 2.51, 95% CI: 1.79–3.53), driven by interventions that reduced material barriers through financial incentives, practical assistance, and extended resource provision. Mental health and emotional support integration showed the second-largest effect (OR = 2.01, 95% CI: 1.19–3.41), consistent with the high co-occurrence of depression, anxiety, and social isolation among disadvantaged pregnant smokers. Specialized training for vulnerability showed a small but statistically significant benefit (OR = 1.29, 95% CI: 1.03–1.61), supporting investment in workforce capacity for vulnerability-sensitive care. Involvement of local communities and reflecting cultural values showed a positive but non-significant pooled effect (OR = 1.38, 95% CI: 0.95–2.00), likely reflecting underpowered individual trials rather than a true absence of effect. Language and education considerations - the most frequently reported approach across included studies - did not demonstrate a statistically significant benefit (OR = 1.13, 95% CI: 0.93–1.37), suggesting that linguistic and literacy adjustments alone, without accompanying structural or psychological support, are insufficient to drive cessation outcomes in disadvantaged populations. GRADE certainty of evidence was low to very low across all vulnerability subgroups, primarily due to high heterogeneity and risk of bias concerns. Full GRADE ratings are presented in Supplementary Material 8. Given these certainty ratings and the between-study nature of subgroup comparisons, these findings should be interpreted as exploratory. The five vulnerability approaches and their pooled effect estimates are summarized in Table 1 and Figure 2.

**TABLE 1:** Pooled effects of behavioral and psychosocial smoking cessation interventions for pregnant women by vulnerability approach subgroup (multiple countries, 1985–2023).

Vulnerability approach	Number of studies	Pooled odds ratio (95% confidence interval)	I^2^, %
Language and education considerations	18	1.13 (0.93–1.37)	30
Socioeconomic inclusiveness	10	2.51 (1.79–3.53)	65
Specialized training for vulnerability	15	1.29 (1.03–1.61)	49
Mental health and emotional support	7	2.01 (1.19–3.41)	82
Involvement of local communities and reflecting cultural values	12	1.38 (0.95–2.00)	56
Any vulnerability approach	23	1.55 (1.32–1.82)	72
No explicit vulnerability approach	27	1.51 (1.14–2.00)	–

Odds ratios greater than 1 favor the intervention. Individual studies could employ more than one vulnerability approach and therefore contribute to more than one subgroup. The certainty of the evidence, according to the Grading of Recommendations Assessment, Development and Evaluation framework, was low to very low for all vulnerability subgroup comparisons (Supplementary Material 8). I^2^ = percentage of total variability attributable to between-study heterogeneity; – = not reported. Subgroup labels correspond to the subgroup headings in Figure 2.

**FIGURE 2:**
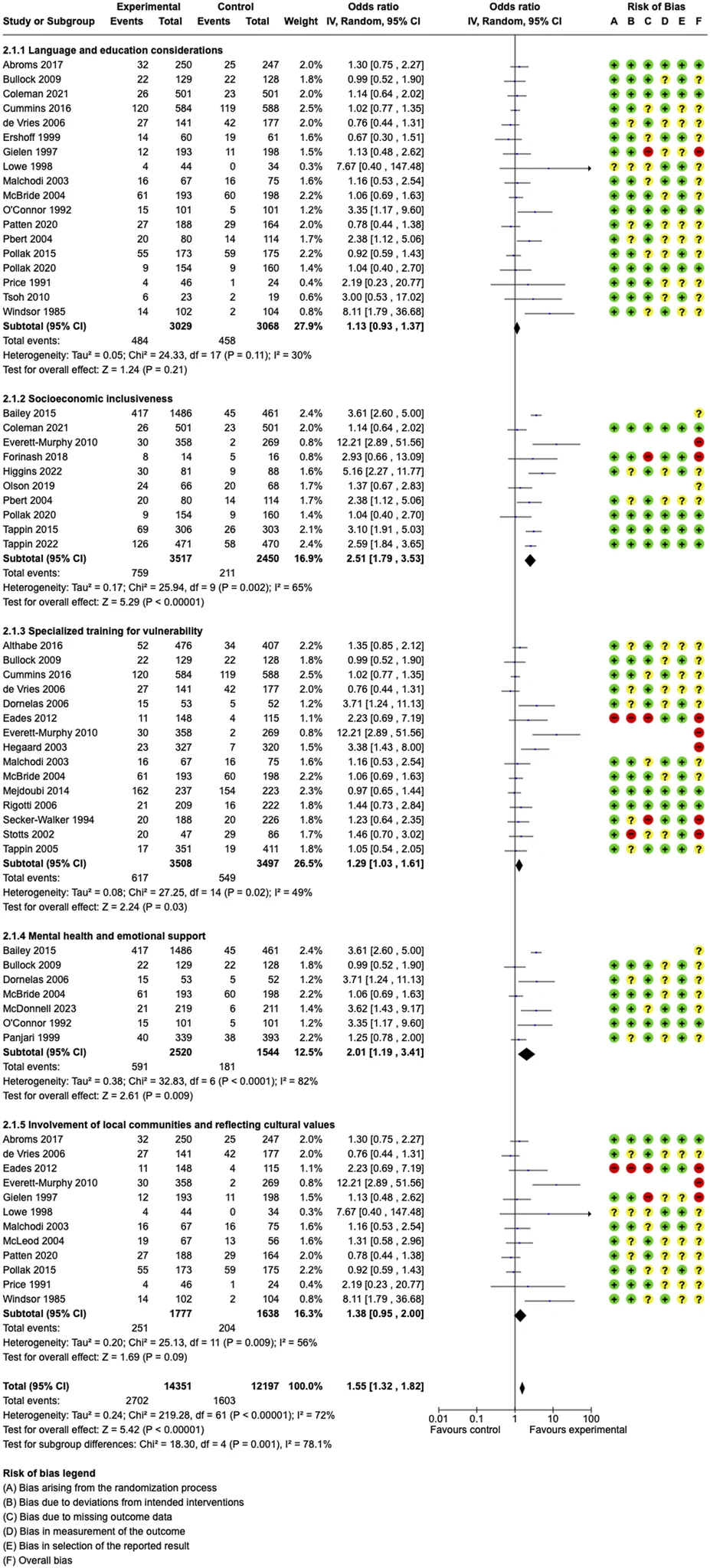
Forest plot of maternal smoking cessation outcomes by vulnerability approach subgroup from random-effects meta-analysis (multiple countries, 1985–2023). Studies employing more than one vulnerability approach contribute to more than one subgroup; the Total row therefore pools 62 study-subgroup contributions from 23 unique studies.

### Expectant father involvement in maternal smoking cessation

Of the 58 included studies, 22 involved interventions for expectant fathers, of which 15 assigned fathers an active role and 7 a passive role (Supplementary Table 6.4, Supplementary Material 6). Active involvement included father-only cessation interventions (e.g., Luk et al. [77], who evaluated brief advice, nicotine replacement therapy sampling, and active referral for expectant fathers; Xia et al. [78], who used a video-based intervention focused on maternal and child health to promote quitting), couple-based interventions (e.g., Pollak et al. [79], who compared written materials and NRT with additional couple-based counseling), and counseling that included fathers alongside pregnant women (McBride et al. [80], Stotts et al. [81]). Some active-involvement studies provided fathers with intervention materials specifically tailored to them (Stanton et al. [82]). Passive involvement was limited to support or witness roles: fathers were invited to accompany women to antenatal visits (Britton et al. [83], Eades et al. [76]), sign support contracts (Lowe et al. [84]), or were personalized to according to their own smoking status (Coleman et al. [85], McLeod et al. [86]).

The meta-analysis of 22 father-involving studies found no statistically significant overall benefit for maternal smoking cessation (OR = 1.12, 95% CI: 0.93–1.35; GRADE: low certainty). Subgroup analysis showed a positive but non-significant trend for active involvement (OR = 1.20, 95% CI: 0.90–1.59; GRADE: very low) and no benefit for passive involvement (OR = 1.05, 95% CI: 0.81–1.36; GRADE: moderate). Notably, interventions not involving fathers showed a larger and statistically significant effect on maternal cessation (OR = 1.85, 95% CI: 1.51–2.27; GRADE: very low), a pattern most likely attributable to the higher intervention intensity and greater format diversity in that subgroup rather than a beneficial effect of excluding fathers *per se*. Forest plots are presented in Supplementary Material 7.

### Paternal smoking cessation

Four studies evaluated father-directed interventions with paternal smoking cessation as the primary outcome, including brief advice with nicotine replacement therapy sampling and active referral [77], a video-based intervention focusing on maternal and child health [78], an RCT directed specifically at expectant fathers [82], and a partner-assisted cessation program [87]. The pooled effect was OR = 2.18 (95% CI: 1.67–2.83; I^2^ = 0%; Figure 3), indicating highly significant and consistent effectiveness across all four studies. Notably, this is the highest-certainty finding in the entire review: GRADE evidence was rated high, reflecting low and consistent heterogeneity, low overall risk of bias, and a narrow confidence interval.

**FIGURE 3:**
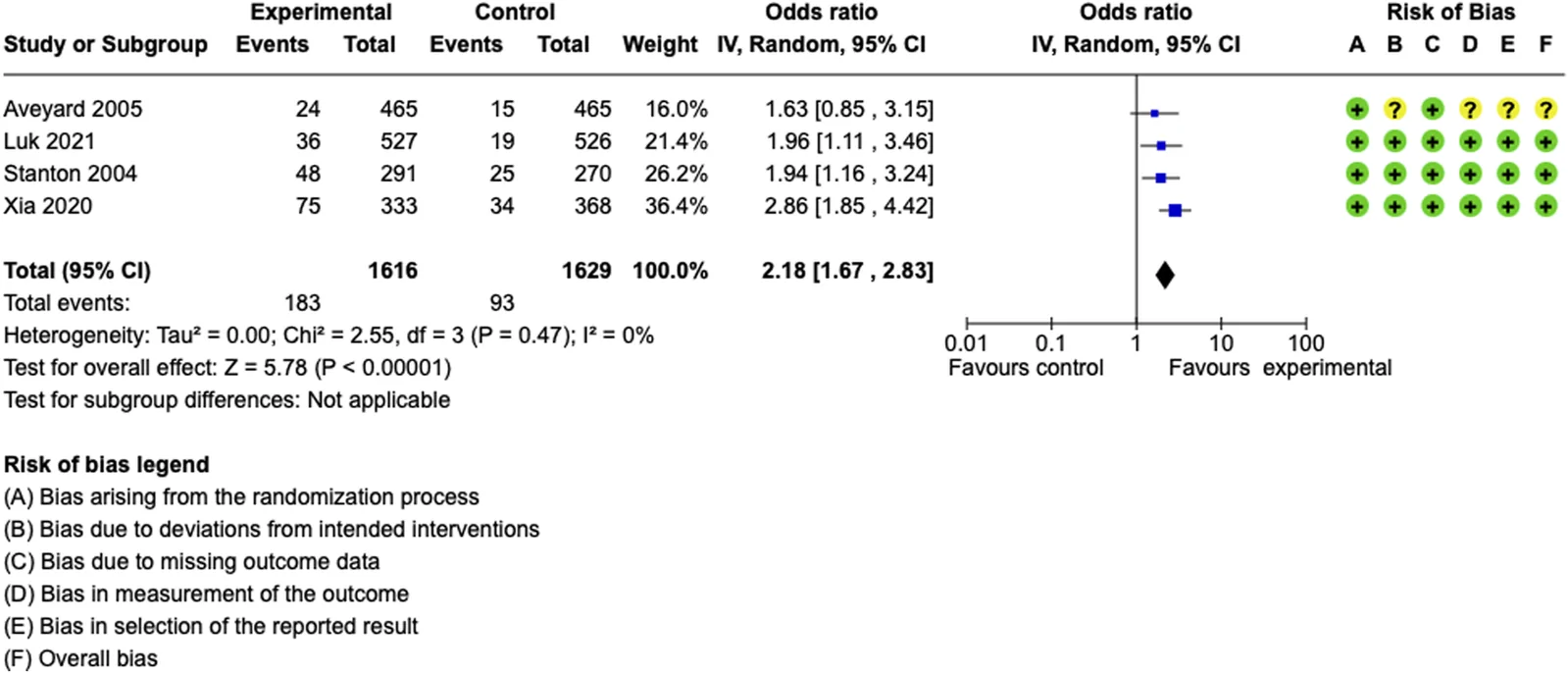
Forest plot of paternal smoking cessation outcomes from father-directed interventions from random-effects meta-analysis (Australia, China, Hong Kong SAR, and the United Kingdom, 2004-2021).

### Second-hand smoke exposure outcomes

Four studies focused exclusively on SHS exposure outcomes in pregnant non-smokers [72, 88–90]. Due to substantial heterogeneity in SHS measurement—including household smoking rules, self-reported exposure, urinary cotinine, and exhaled carbon monoxide—no meta-analysis was performed. Yang et al. [88], Soltani et al. [89], and Herbert et al. [72] each reported significant reductions in SHS exposure; by contrast, Alemán et al. [90] found that brief individual counseling alone was insufficient, highlighting the need for household-level approaches. These findings suggest that partner and household engagement are critical for effective SHS reduction.

### Risk of bias

Among the included RCTs, the majority were rated as presenting some concerns or high risk of bias on at least one domain. The most common concerns were deviations from intended interventions, inherent to the open-label nature of behavioral trials, and missing outcome data due to participant dropout. Non-randomized studies showed a moderate-to-serious risk of bias on the ROBINS-I, primarily due to confounding and selection bias. Sensitivity analyses excluding high-risk studies did not substantially alter the direction of pooled effects in most comparisons, though confidence intervals were wider. High statistical heterogeneity (I^2^ > 50%) was observed in several subgroup analyses, including those for individual counseling and telephone-based interventions, reducing confidence in the pooled estimates. The observed heterogeneity likely reflects variation in populations, intervention intensity, and implementation contexts rather than inconsistency in the direction of effect. Egger’s regression test confirmed no evidence of publication bias in the overall maternal meta-analysis (intercept = 0.05, t = 0.29, p = 0.78). Significant Egger’s tests in four vulnerability subgroups were characterized by negative intercepts in three of four cases, consistent with within-subgroup heterogeneity (I^2^ 30%–82%) rather than selective publication; a borderline-significant positive intercept was observed in the no-father-involvement comparison (intercept = 0.36, p = 0.08). Full Egger’s test results are provided in Supplementary Material 7. Complete risk of bias ratings is provided in Supplementary Material 4.

## Discussion

### Summary of main findings

This systematic review and meta-analysis synthesizes evidence from 58 studies on the effectiveness of behavioral and psychosocial smoking cessation interventions during pregnancy, with the primary analytical focus on two dimensions that existing reviews have not systematically addressed: approaches to sociocultural vulnerability and expectant father involvement. While confirming that behavioral interventions are broadly effective across delivery formats—consistent with the Cochrane evidence base [21, 66]—the review’s central contribution lies in demonstrating that how an intervention addresses vulnerability determines its reach and equity impact, and that the case for targeting expectant fathers rests primarily on paternal rather than maternal outcomes.

The vulnerability subgroup analyses reveal that not all approaches to diversity and disadvantage are equally effective, and that this difference matters considerably for program design. Socioeconomic inclusiveness—addressing material barriers through financial incentives and tangible practical support—and mental health integration both yielded effect sizes (OR = 2.51 and 2.01, respectively) substantially higher than the overall pooled effect, whereas language and education considerations alone, despite being the most commonly implemented approach, showed no statistically significant benefit [91–93]. This pattern suggests that practitioners and policymakers who rely primarily on translated materials or plain-language adaptations as their equity strategy may be underinvesting in the structural and psychological dimensions of disadvantage that more strongly predict cessation success. Implementation fidelity also emerged as a cross-cutting moderator: studies reporting insufficient counselor training, low contact frequency, and time-constrained antenatal visits consistently showed attenuated effects [68, 71, 74, 94], indicating that service delivery conditions must be addressed alongside intervention content. These subgroup comparisons are between-study observations of low to very low certainty; they generate hypotheses about which equity strategies are most promising rather than confirming differential effectiveness.

The finding that involvement of local communities and reflecting cultural values showed a clinically meaningful but non-significant pooled effect (OR = 1.38) warrants particular attention. Studies such as Patten et al. [75] in rural Alaska and Eades et al. [76] in Aboriginal communities in Australia demonstrated promising effects from co-produced, culturally grounded interventions. The non-significance of the overall estimate likely reflects the small number of such studies and their heterogeneous designs, rather than an absence of true effect. The strong theoretical rationale for community co-production [16, 95], combined with the persistent underrepresentation of Indigenous, migrant, and minority ethnic communities in cessation trials, makes this an urgent priority for future investment.

The concentration of evidence in high-income countries also constrains the transferability of these findings. In many low- and middle-income settings, smoking during pregnancy and second-hand smoke exposure occur within different cultural norms, gender dynamics, and tobacco-use patterns, including smokeless tobacco, and antenatal care systems may lack the workforce capacity and financial infrastructure that several of the effective interventions identified here presuppose [12, 95]. Financial-incentive and mental health-integrated models in particular will require careful adaptation and piloting before transfer to resource-constrained health systems, and the near-absence of trials from the settings where the burden of tobacco exposure in pregnancy is greatest is itself a key evidence gap.

The findings on father involvement require careful reframing. The non-significant effect of father involvement on maternal cessation (OR = 1.12) should not be interpreted as evidence that targeting fathers is ineffective or unhelpful. Rather, it reflects the mismatch between the intervention target (fathers) and the outcome measure (maternal cessation): including a father in a maternal cessation program does not straightforwardly translate into better maternal outcomes, particularly when the father’s own smoking behavior is not directly addressed. The review’s most important finding in this domain is that when expectant fathers are the explicit primary target of cessation support—whether through brief advice and NRT sampling [77], video-based behavior change [78], or a father-targeted randomized trial [82]—the results are exceptional: OR = 2.18, I^2^ = 0%, GRADE high certainty. This is the only high-certainty finding in the review. Even so, it rests on four trials, and replication in larger and more diverse samples is needed before firm implementation recommendations can be made. The pregnancy period is a well-documented window of heightened cessation motivation for men [17, 96, 97], and father-directed interventions act on this motivation directly, reducing household SHS exposure and creating a smoke-free environment that protects both mother and child. This contrast suggests a shift in emphasis: instead of asking whether including a father improves maternal outcomes, the field should ask how best to help expectant fathers quit. Evidence suggests that autonomy-supportive, sex-specific approaches aligned with fathers’ preferences are more effective than couple-based formats [20, 79] and that masculine identity and stress may need explicit attention in intervention design [98].

### Practical implications

The findings carry direct implications for how antenatal smoking cessation programs are designed and delivered. For programs targeting pregnant women from disadvantaged backgrounds, adding financial incentives and practical support (housing, transport, food assistance) alongside cessation counseling was associated with substantially larger pooled effects and merits consideration as an equity strategy, although the low certainty of the subgroup evidence means such approaches should be introduced with concurrent evaluation [69, 93, 99]. Similarly, integrating mental health and emotional support into cessation programs—rather than treating it as a specialist referral pathway—addresses the psychological co-morbidities that disproportionately burden the most disadvantaged pregnant smokers [73, 100]. Training midwives, nurses, and community health workers in vulnerability-sensitive communication and cessation counseling is a scalable, cost-effective investment with measurable benefit [71, 95]. For programs addressing cultural and linguistic diversity, the evidence suggests that linguistic adaptation alone is insufficient: interventions need to be co-produced with communities and must address structural and psychological barriers alongside language and literacy [75, 76]. Regarding expectant fathers, brief, father-directed cessation support, delivered at routine antenatal contact points, was highly effective in the available trials and could be considered for integration into antenatal care pathways [77, 78, 97]. Because this evidence derives from four trials, implementation should be accompanied by ongoing evaluation, and policymakers designing national smoking cessation programs for pregnancy may consider including a father-targeted component.

Finally, the scope of this review was deliberately restricted to behavioral and psychosocial interventions. Pharmacological cessation therapies during pregnancy involve distinct safety, dosing, and adherence considerations and have been comprehensively synthesized in a dedicated Cochrane review [22]; restricting the scope allowed a coherent analysis of the sociocultural and relational dimensions of intervention design that pharmacological trials rarely report. Population-level environmental and policy measures, such as smoke-free legislation and taxation, act through different mechanisms and require different evaluative designs, and were likewise beyond the scope of this review.

### Limitations

The primary limitation is that machine learning-assisted screening via ASReview excluded approximately 80% of records based on algorithmic ranking without full human review. Although the stopping criterion was pre-specified and applied conservatively, all included studies were identified well before the stopping threshold was reached, and validation studies report recall comparable to full manual screening [25–28], a residual risk remains that atypically indexed relevant studies were missed. The predominance of high-income country settings limits generalizability to low- and middle-income contexts, where the global burden of smoking during pregnancy is greatest. High statistical heterogeneity in several meta-analytic comparisons reduces confidence in pooled estimates, and GRADE certainty was low to very low for most vulnerability subgroup comparisons. Variability in outcome definitions, measurement timing, and verification methods further complicates interpretation. In particular, exclusively self-reported cessation outcomes are vulnerable to social desirability and recall bias, pressures amplified during pregnancy, and may overestimate cessation relative to biochemically verified outcomes; pooled estimates should be read with this in mind, and future trials should incorporate biochemical verification such as salivary or urinary cotinine or exhaled carbon monoxide. Evidence on father involvement in maternal cessation remains limited in volume and quality. Funnel plot asymmetry in some subgroup comparisons was not confirmed as systemic publication bias by Egger’s regression test in the overall analysis (p = 0.78), with significant subgroup-level tests most plausibly attributable to heterogeneity. Two further limitations merit acknowledgement. First, this review was strictly quantitative: trial-level variables are largely descriptive and cannot illuminate the mechanisms through which factors such as partner support influence smoking behavior, and such unmeasured processes may contribute to the observed heterogeneity; qualitative and theory-based syntheses are needed to understand how, and under what conditions, these interventions work. Second, the restriction to controlled trial designs may itself exclude the most vulnerable populations, who are frequently underrepresented in trials; future syntheses incorporating qualitative studies and a broader range of designs would enable a more inclusive evidence base.

## Conclusion

This review adds to the existing evidence base on smoking cessation in pregnancy by systematically characterizing two dimensions that prior reviews have not fully addressed. First, vulnerability-adapted approaches are not uniformly effective: interventions that address socioeconomic barriers and integrate mental health support appeared in exploratory subgroup analyses to outperform those that focus solely on language and education adaptation and may warrant confirmatory evaluation and prioritization in program design for diverse and disadvantaged families. Second, and most distinctively, father-directed cessation interventions are highly effective for paternal smoking cessation (OR = 2.18; high-certainty evidence)—the strongest and most consistent finding in this review, albeit one based on four trials. The pregnancy period is an underutilized window for father-targeted cessation support, and integrating brief father-directed interventions into antenatal care, accompanied by further evaluation across settings, offers both individual and household-level benefits. Future research should prioritize co-produced, culturally grounded interventions in low- and middle-income settings, standardize cessation and SHS outcome measures, evaluate family-level outcomes to capture the full impact of father-involving approaches, and integrate qualitative and theory-based approaches to elucidate the mechanisms through which social and relational factors shape intervention effectiveness.
