# Supplementary material for: Smoking cessation in pregnancy: a systematic review and meta-analysis of vulnerability approaches and expectant father involvement
Source: Public Health Rev. 2026 Sep 14;47:1609825. doi: 10.3389/phrs.2026.1609825 (PMC13616806; doi:10.3389/phrs.2026.1609825)
Supplement: Supplementary file 1 [file DataSheet1.pdf]

Table of Contents

1. PRISMA CHECKLIST **2**

2. SEARCH STRATEGY **6**

3. SCREENING FOR INCLUSION AND EXCLUSION **10**

I. MACHINE-LEARNING AIDED SCREENING BY TITLE AND ABSTRACT WITH ASREVIEW LAB SOFTWARE **10**

II. REASONS FOR EXCLUSION DURING FULL-TEXT SCREENING **11**

4. RISK OF BIAS ASSESSMENTS **13**

5. GENERAL CHARACTERISTICS OF INCLUDED STUDIES **16**

6. DETAILED CHARACTERISTICS OF INCLUDED STUDIES **28**

TABLE 6.1. DETAILED INTERVENTION DESCRIPTION 28
TABLE 6.2. DETAILED OUTCOMES AND EFFECT SIZES OF EACH INTERVENTION 51
TABLE 6.3. DETAILED VULNERABILITY CHARACTERISTICS AND APPROACH 68
TABLE 6.4. DETAILED DESCRIPTION OF FATHER INVOLVEMENT 76

7. META-ANALYSIS RESULTS **84**

I. FOREST PLOTS OF RESULTS **84**

II. FUNNEL PLOTS OF RESULTS **99**

III. EGGER'S TEST RESULTS **104**

8. GRADE ASSESSMENT **105**

1. PRISMA CHECKLIST

Table 1.1. PRISMA Checklist

Section and Topic	Item #	Checklist item	Location where item is reported
**TITLE**			
Title	1	Identify the report as a systematic review.	Title
**ABSTRACT**			
Abstract	2	See the PRISMA 2020 for Abstracts checklist.	Abstract
**INTRODUCTION**			
Rationale	3	Describe the rationale for the review in the context of existing knowledge.	Background section
Objectives	4	Provide an explicit statement of the objective(s) or question(s) the review addresses.	Background section
**METHODS**			
Eligibility criteria	5	Specify the inclusion and exclusion criteria for the review and how studies were grouped for the syntheses.	Methods section: Eligibility criteria
Information sources	6	Specify all databases, registers, websites, organisations, reference lists and other sources searched or consulted to identify studies. Specify the date when each source was last searched or consulted.	Methods section: Information sources
Search strategy	7	Present the full search strategies for all databases, registers, and websites, including any filters and limits used.	Methods section: Search strategy Supplementary file 2
Selection process	8	Specify the methods used to decide whether a study met the inclusion criteria of the review, including how many reviewers screened each record and each report retrieved, whether they worked independently, and if applicable, details of automation tools used in the process.	Methods section: Source of evidence selection
Data collection process	9	Specify the methods used to collect data from reports, including how many reviewers collected data from each report, whether they worked independently, any processes for obtaining or confirming data from study investigators, and if applicable, details of automation tools used in the process.	Methods section: Data extraction
Data items	10a	List and define all outcomes for which data were sought. Specify whether all results that were compatible with each outcome domain in each study were sought (e.g. for all measures, time points, analyses), and if not, the methods used to decide which results to collect.	Methods section: Eligibility criteria, Data extraction

Section and Topic	Item #	Checklist item	Location where item is reported
	10b	List and define all other variables for which data were sought (e.g. participant and intervention characteristics, funding sources). Describe any assumptions made about any missing or unclear information.	Methods section: Data charting process
Study risk of bias assessment	11	Specify the methods used to assess risk of bias in the included studies, including details of the tool(s) used, how many reviewers assessed each study and whether they worked independently, and if applicable, details of automation tools used in the process.	Methods section: Quality of evidence
Effect measures	12	Specify for each outcome the effect measure(s) (e.g. risk ratio, mean difference) used in the synthesis or presentation of results.	Methods section: Data synthesis
Synthesis methods	13a	Describe the processes used to decide which studies were eligible for each synthesis (e.g. tabulating the study intervention characteristics and comparing against the planned groups for each synthesis (item #5)).	Methods section: Data synthesis and analysis
	13b	Describe any methods required to prepare the data for presentation or synthesis, such as handling of missing summary statistics, or data conversions.	Methods section: Data synthesis and analysis
	13c	Describe any methods used to tabulate or visually display results of individual studies and syntheses.	Methods section: Data synthesis and analysis
	13d	Describe any methods used to synthesize results and provide a rationale for the choice(s). If meta-analysis was performed, describe the model(s), method(s) to identify the presence and extent of statistical heterogeneity, and software package(s) used.	Methods, data synthesis, heterogeneity assessment
	13e	Describe any methods used to explore possible causes of heterogeneity among study results (e.g. subgroup analysis, meta-regression).	Methods section: Data synthesis and analysis
	13f	Describe any sensitivity analyses conducted to assess robustness of the synthesized results.	Methods section: Data synthesis and analysis<br>Strengths and Limitations
Reporting bias assessment	14	Describe any methods used to assess risk of bias due to missing results in a synthesis (arising from reporting biases).	Methods section: Quality of the evidence and risk of bias assessment

Section and Topic	Item #	Checklist item	Location where item is reported
			Supplementary file 4
Certainty assessment	15	Describe any methods used to assess certainty (or confidence) in the body of evidence for an outcome.	Methods, study quality<br>Supplementary file 8
**RESULTS**			
Study selection	16a	Describe the results of the search and selection process, from the number of records identified in the search to the number of studies included in the review, ideally using a flow diagram.	Study results: Figure 1
	16b	Cite studies that might appear to meet the inclusion criteria, but which were excluded, and explain why they were excluded.	Supplementary file 3
Study characteristics	17	Cite each included study and present its characteristics.	Supplementary file 5<br>Supplementary file 6
Risk of bias in studies	18	Present assessments of risk of bias for each included study.	Results section Supplementary file 4
Results of individual studies	19	For all outcomes, present, for each study: (a) summary statistics for each group (where appropriate) and (b) an effect estimate and its precision (e.g. confidence/credible interval), ideally using structured tables or plots.	Supplementary file 7
Results of syntheses	20a	For each synthesis, briefly summarise the characteristics and risk of bias among contributing studies.	Methods section: Quality of the evidence and risk of bias assessment
	20b	Present results of all statistical syntheses conducted. If meta-analysis was done, present for each the summary estimate and its precision (e.g. confidence/credible interval) and measures of statistical heterogeneity. If comparing groups, describe the direction of the effect.	Results section: figures 2-5<br>Supplementary file 7
	20c	Present results of all investigations of possible causes of heterogeneity among study results.	Supplementary file 7
	20d	Present results of all sensitivity analyses conducted to assess the robustness of the synthesized results.	Supplementary file 7
Reporting biases	21	Present assessments of risk of bias due to missing results (arising from reporting biases) for each synthesis assessed.	Supplementary file 4<br>Supplementary file 7
Certainty of evidence	22	Present assessments of certainty (or confidence) in the body of evidence for each outcome assessed.	Results section<br>Supplementary file 8
**DISCUSSION**			
Discussion	23a	Provide a general interpretation of the results in the context of other evidence.	Discussion section
	23b	Discuss any limitations of the evidence included in the review.	Discussion section:

Section and Topic	Item #	Checklist item	Location where item is reported
			Strengths, and limitations
	23c	Discuss any limitations of the review processes used.	Discussion section: Strengths, and limitations
	23d	Discuss implications of the results for practice, policy, and future research.	Discussion section: Conclusion
**OTHER INFORMATION**			
Registration and protocol	24a	Provide registration information for the review, including register name and registration number, or state that the review was not registered.	Methods section
	24b	Indicate where the review protocol can be accessed, or state that a protocol was not prepared.	Methods section
	24c	Describe and explain any amendments to information provided at registration or in the protocol.	Methods section
Support	25	Describe sources of financial or non-financial support for the review, and the role of the funders or sponsors in the review.	Declarations: funding
Competing interests	26	Declare any competing interests of review authors.	Declarations: Competing interests
Availability of data, code and other materials	27	Report which of the following are publicly available and where they can be found: template data collection forms; data extracted from included studies; data used for all analyses; analytic code; any other materials used in the review.	Supplementary files publicly available

Note: This table is based on the PRISMA 2020 statement

Reference:

1. Page MJ, McKenzie JE, Bossuyt PM, et al. The PRISMA 2020 statement: An updated guideline for reporting systematic reviews. BMJ. 2021;372(n71). doi: https://doi.org/10.1136/bmj.n71.

2. SEARCH STRATEGY

Search conducted in all databases on 20th.October. 2023

Population Rationale: *all terms considered relevant for the population of pregnant women were considered and synonyms were added based on other relevant sources previously found. Expectant fathers alone are not explicitly included, since the main population target are expectant mothers, however the inclusion of expectant fathers in the interventions will be consider in the analysis of the review.*

Intervention Rationale*: terms included are characterized in two: the ones directed to smoking reduction or cessation and the ones addressing the tobacco control in the home environment. Not included are terms related to smoke-free environments in places different than home, such as workplace or other social environments.*

Table 2.1. Search syntax for Medline via PubMed

QUERY	SEARCH DETAILS	RESULTS
#1 population terms	"pregnan*"[Title/Abstract] OR "expectant mother*"[Title/Abstract] OR "expecting mother*"[Title/Abstract] OR "postpartum"[Title/Abstract] OR "prenatal"[Title/Abstract]	721,009
#2 population MESH terms	"pregnant women"[MeSH Terms]	14,810
#3 ALL population syntax	#1 OR #2	724,304
#4 intervention terms	"smoking cessation"[Title/Abstract] OR "smoke free"[Title/Abstract] OR "smoking prevention"[Title/Abstract] OR "quitting smoking"[Title/Abstract] OR "smoking reduction"[Title/Abstract] OR "cessation intervention*"[Title/Abstract] OR "cessation strateg*"[Title/Abstract] OR "tobacco use cessation*"[Title/Abstract] OR "smoke free home*"[Title/Abstract]	37,622
#5 intervention MESH terms	"smoking cessation"[MeSH Terms] OR "smoking prevention"[MeSH Terms] OR "tobacco control"[MeSH Terms]	45,857
#6 ALL intervention syntax	#4 OR #5	61,188
#7 **ALL blocks together**	#3 AND #6<br>No filter applied	3,118

Table 2.2. Search syntax for Scopus

QUERY	SEARCH DETAILS	RESULTS
#1 population terms	"pregnan*"[Title/Abstract] OR "expectant mother*"[Title/Abstract] OR "expecting mother*"[Title/Abstract] OR "postpartum"[Title/Abstract] OR "prenatal"[Title/Abstract]	853,448
#2 intervention terms	"smoking cessation"[Title/Abstract] OR "smoke free"[Title/Abstract] OR "smoking prevention"[Title/Abstract] OR "quitting smoking"[Title/Abstract] OR "smoking reduction"[Title/Abstract] OR "cessation intervention*"[Title/Abstract] OR "cessation strateg*"[Title/Abstract] OR "tobacco use cessation*"[Title/Abstract] OR "smoke free home*"[Title/Abstract]	42,578
#3 **ALL blocks together**	#1 AND #2<br>No filters applied	2,492

Table 2.3. Search syntax for CINAHL via EBSCOhost

QUERY	SEARCH DETAILS	RESULTS
S1 population terms	(TI pregnan* OR AB pregnan*) OR (TI "expectant mother*" OR AB "expectant mother*") OR (TI "expecting mother*" OR AB "expecting mother*") OR (TI postpartum OR AB postpartum) OR (TI prenatal OR AB prenatal)	204,985
S2 population MESH terms	(MH "Expectant Mothers")	13,854
S3 ALL population syntax	S1 OR S2	207,768
S4 intervention terms	(TI "smoking cessation" OR AB "smoking cessation") OR (TI "smoke free" OR AB "smoke free") OR (TI "smoking prevention" OR AB "smoking prevention") OR (TI "quitting smoking" OR AB "quitting smoking") OR (TI "smoking reduction" OR AB "smoking reduction") OR (TI "cessation intervention*" OR AB "cessation intervention*") OR (TI "cessation strateg*" OR AB "cessation strateg*") OR (TI "tobacco use cessation*" OR AB "tobacco use cessation*") OR (TI "smoke free home*" OR AB "smoke free home*")	19,639
S5 intervention MESH terms	(MH "smoking cessation" OR MH "tobacco control" OR MH "smoking cessation programs")	25,110
S6 ALL intervention syntax	S4 OR S5	32,451
S7 **ALL blocks together**	S3 AND S6<br>No filters applied	1,881

Table 2.4. Search syntax for PsycInfo via Ovid

QUERY	SEARCH DETAILS	RESULTS
1 population terms	(pregnan* or "expectant mother*" or "expecting mother*" or postpartum or prenatal).ti. or (pregnan* or "expectant mother*" or "expecting mother*" or postpartum or prenatal).ab.	73,322
2 population MESH terms	exp expectant mothers/	1,051
3 ALL population syntax	1 OR 2	73,426
4 intervention terms	("smoking cessation" or "smoke free" or "smoking prevention" or "quitting smoking" or "smoking reduction" or "cessation intervention*" or "cessation strateg*" or "tobacco use cessation*" or "smoke free home*").ti. or ("smoking cessation" or "smoke free" or "smoking prevention" or "quitting smoking" or "smoking reduction" or "cessation intervention*" or "cessation strateg*" or "tobacco use cessation*" or "smoke free home*").ab.	15,406
5 intervention MESH terms	exp smoking cessation/ OR exp smoking prevention/ OR exp tobacco control/	15,114
6 ALL intervention syntax	4 OR 5	20,115
7 **ALL blocks together**	3 AND 6<br>No filters applied	1,102

Table 2.5. Search syntax for ASSIA via ProQuest

QUERY	SEARCH DETAILS	RESULTS
S1 population terms	abstract((pregnan* or "expectant mother*" or "expecting mother*" or postpartum or prenatal)) OR title((pregnan* or "expectant mother*" or "expecting mother*" or postpartum or prenatal))	25,953
S2 population MESH terms	MAINSUBJECT.EXACT.EXPLODE("Pregnant women")	2,933
S3 ALL population syntax	S1 OR S2	26,248
S4 intervention terms	abstract(("smoking cessation" or "smoke free" or "smoking prevention" or "quitting smoking" or "smoking reduction" or "cessation intervention*" or "cessation strateg*" or "tobacco use cessation*" or "smoke free home*")) OR title(("smoking cessation" or "smoke free" or "smoking prevention" or "quitting smoking" or "smoking reduction" or "cessation intervention*" or "cessation strateg*" or "tobacco use cessation*" or "smoke free home*"))	5,592
S5 intervention MESH terms	MAINSUBJECT.EXACT.EXPLODE("Cessation")	5,148
S6 ALL intervention syntax	S4 OR S5	7,617
S7 **ALL blocks together**	S3 AND S6<br>No filters applied	452

Table 2.6. Summary of obtained results in all databases

**Database**	**Results**
Medline via PubMed	3118
Scopus	2492
CINAHL via EBSCOhost	1881
Psycinfo via Ovid	1102
ASSIA via ProQuest	452
**Total:**	9045

3. SCREENING FOR INCLUSION AND EXCLUSION

I. Machine-learning aided screening by title and abstract with ASReview Lab Software

Screening by title and abstract conducted between 21st.October.2023 and 15th.November.2023

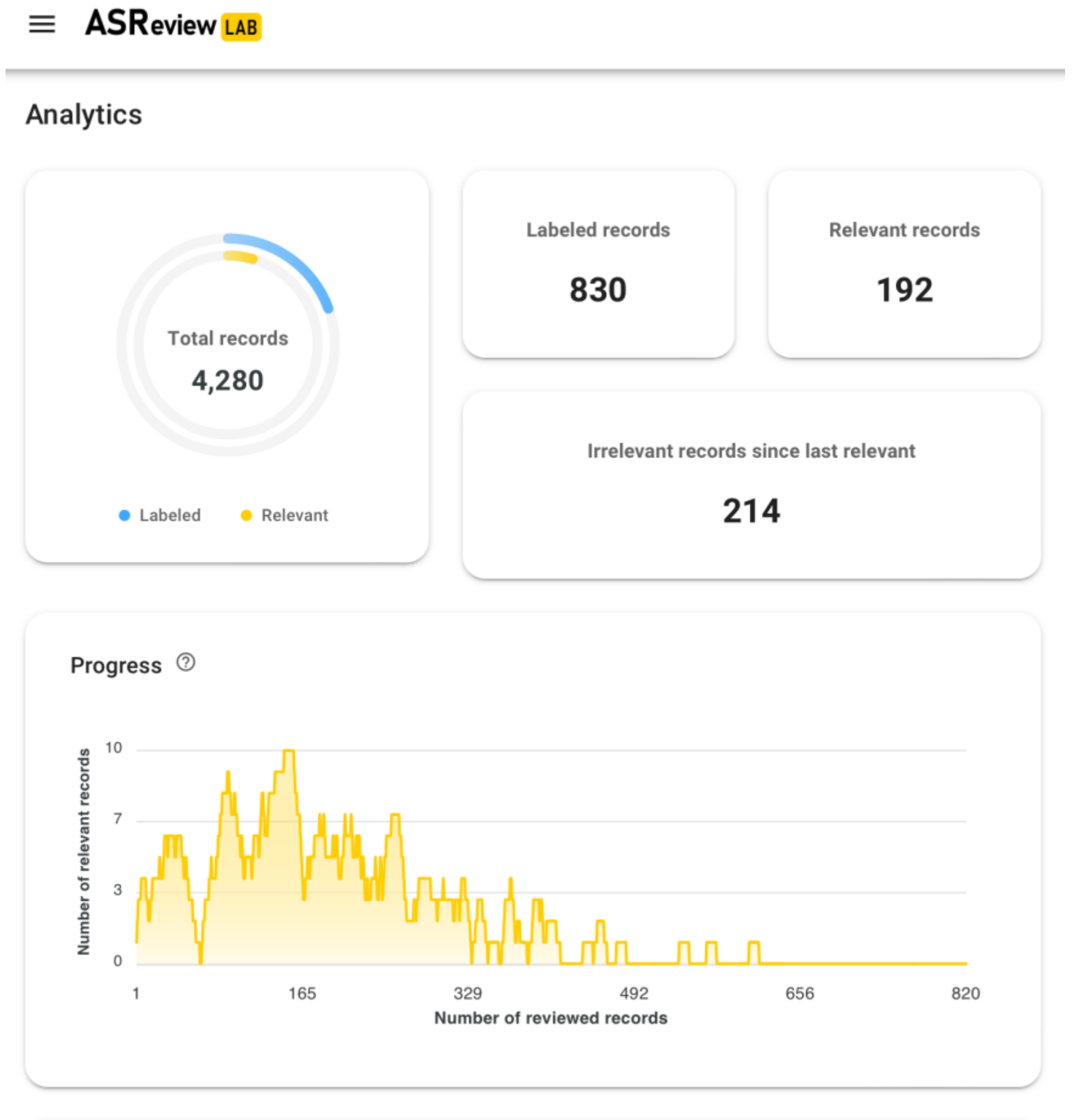

II. Reasons For Exclusion During Full-Text Screening

First author name, year of publication and article reference	Reason for exclusion
Gould, 2022 (1)	Design: Protocol for a randomized controlled trial
Agràs-Guàrdia, 2022 (2)	
Van Dijk, 2021 (3)	
Sinclair, 2020 (4)	
Oliete, 2004 (5)	
Lum, 2020 (6)	Design: Systematic review protocol
Kropa, 2006 (7)	Design: Literature review
Gebauer, 1998 (8)	Descriptive design
Risica, 2017 (9)	
Karatay, 2010 (10)	No control group
Mcgowan, 2010 (11)	
Huang, 2013 (12)	Outcome measured: self-efficacy
Hayes, 2013 (13)	Outcome measured: quitting postpartum
Bar-Zeev, 2019 (14)	Outcomes measured: attitude and knowledge
Widsor, 2000 (15)	Outcomes measured: behavioural impact
Aveyard, 2006 (16)	Outcomes measured: state of change in motivation

References:

1. Gould GS, Ryan NM, Kumar R, Stevenson LC, Carson-Chahhoud KV, Oldmeadow C, et al. SISTAQUIT: training health care providers to help pregnant Aboriginal and Torres Strait Islander women quit smoking. A cluster randomised controlled trial. Med J Aust. 2022;217(1):36-42.
2. Agras-Guardia M, Martinez-Torres S, Granado-Font E, Palleja-Millan M, Villalobos F, Patricio D, et al. Effectiveness of an App for tobacco cessation in pregnant smokers (TOBBGEST): study protocol. BMC Pregnancy Childbirth. 2022;22(1):933-undefined.
3. van Dijk W, Oosterman M, Jansen I, de Vente W, Huizink A. Stress- and smoke free pregnancy study protocol: a randomized controlled trial of a personalized eHealth intervention including heart rate variability-biofeedback to support pregnant women quit smoking via stress reduction. BMC Public Health. 2021;21(1):905-undefined.
4. Sinclair L, McFadden M, Tilbrook H, Mitchell A, Keding A, Watson J, et al. The smoking cessation in pregnancy incentives trial (CPIT): study protocol for a phase III randomised controlled trial. Trials. 2020;21(1):183.
5. Blasco Oliete M, Sanz Cuesta T, Girbés Fontana M, Pascual Malanda M, Ortiz Valdepeñas J, García López L. Effectiveness of two health interventions to get pregnant women to give up smoking. Aten Primaria. 2004;33(5):277-83.
6. Lum A, Skelton E, McCarter KL, Handley T, Judd L, Bonevski B. Smoking cessation interventions for people living in rural and remote areas: a systematic review protocol. BMJ Open. 2020;10(11):e041011.
7. Kropa L. What Is The Most Effective Way To Help Pregnant Smokers Quit: Telephone Counseling Or Midwife Delivered Home Based Counseling? The internet journal of academic physician assistants. 2006;6(1).
8. Gebauer C, Kwo CY, Haynes EF, Wewers ME. A nurse-managed smoking cessation intervention during pregnancy. J Obstet Gynecol Neonatal Nurs. 1998;27(1):47-53.
9. Risica PM, Gavarkovs A, Parker DR, Jennings E, Phipps M. A tailored video intervention to reduce smoking and environmental tobacco exposure during and after pregnancy: Rationale, design and methods of Baby's Breath. Contemp Clin Trials. 2017;52:1-9.
10. Karatay G, Kublay G, Emiroğlu ON. Effect of motivational interviewing on smoking cessation in pregnant women. J Adv Nurs. 2010;66(6):1328-37.
11. McGowan A, Hamilton S, Barnett D, Nsofor M, Proudfoot J, Tappin DM. 'Breathe': the stop smoking service for pregnant women in Glasgow. Midwifery. 2010;26(3):e1-e13.
12. Huang CM, Wu HL, Huang SH, Chien LY, Guo JL. Transtheoretical model-based passive smoking prevention programme among pregnant women and mothers of young children. Eur J Public Health. 2013;23(5):777-82.
13. Hayes CB, Collins C, O'Carroll H, Wyse E, Gunning M, Geary M, et al. Effectiveness of motivational interviewing in influencing smoking cessation in pregnant and postpartum disadvantaged women. Nicotine Tob Res. 2013;15(5):969-77.
14. Bar-Zeev Y, Bovill M, Bonevski B, Gruppetta M, Oldmeadow C, Palazzi K, et al. Improving smoking cessation care in pregnancy at Aboriginal Medical Services: 'ICAN QUIT in Pregnancy' step-wedge cluster randomised study. BMJ Open. 2019;9(6):e025293.
15. Windsor RA, Woodby LL, Miller TM, Hardin JM, Crawford MA, DiClemente CC. Effectiveness of Agency for Health Care Policy and Research clinical practice guideline and patient education methods for pregnant smokers in medicaid maternity care. Am J Obstet Gynecol. 2000;182(1 Pt 1):68-75.
16. Aveyard P, Lawrence T, Cheng KK, Griffin C, Croghan E, Johnson C. A randomized controlled trial of smoking cessation for pregnant women to test the effect of a transtheoretical model-based intervention on movement in stage and interaction with baseline stage. Br J Health Psychol. 2006;11(Pt 2):263-78.

4. RISK OF BIAS ASSESSMENTS

The 58 (1-58) selected articles were distributed by design as follows:

Table 4.1. Risk of bias assessment of individual randomised controlled trials[1]

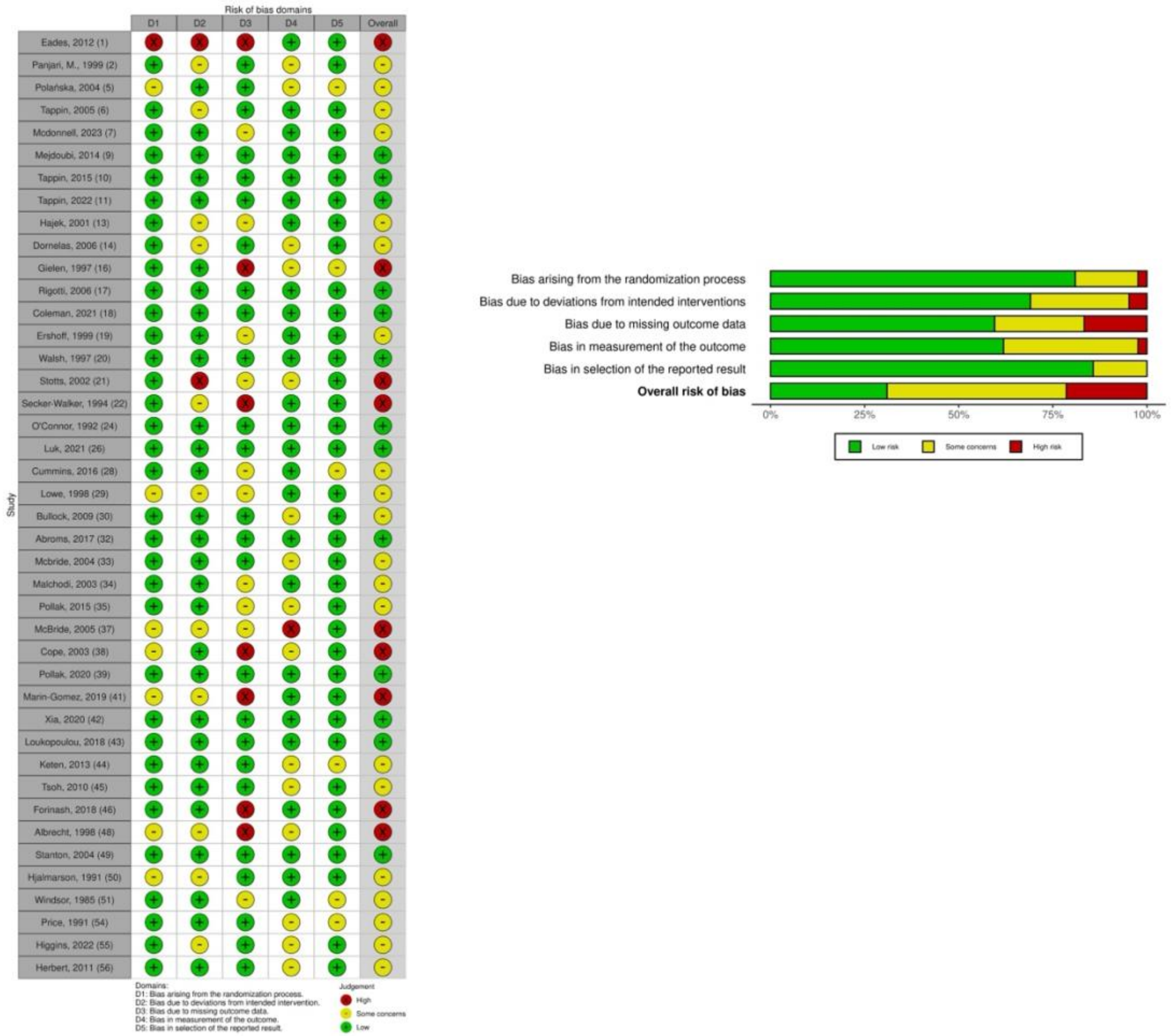

Table 4.2. Risk of bias assessment of cluster randomised controlled trials[2]

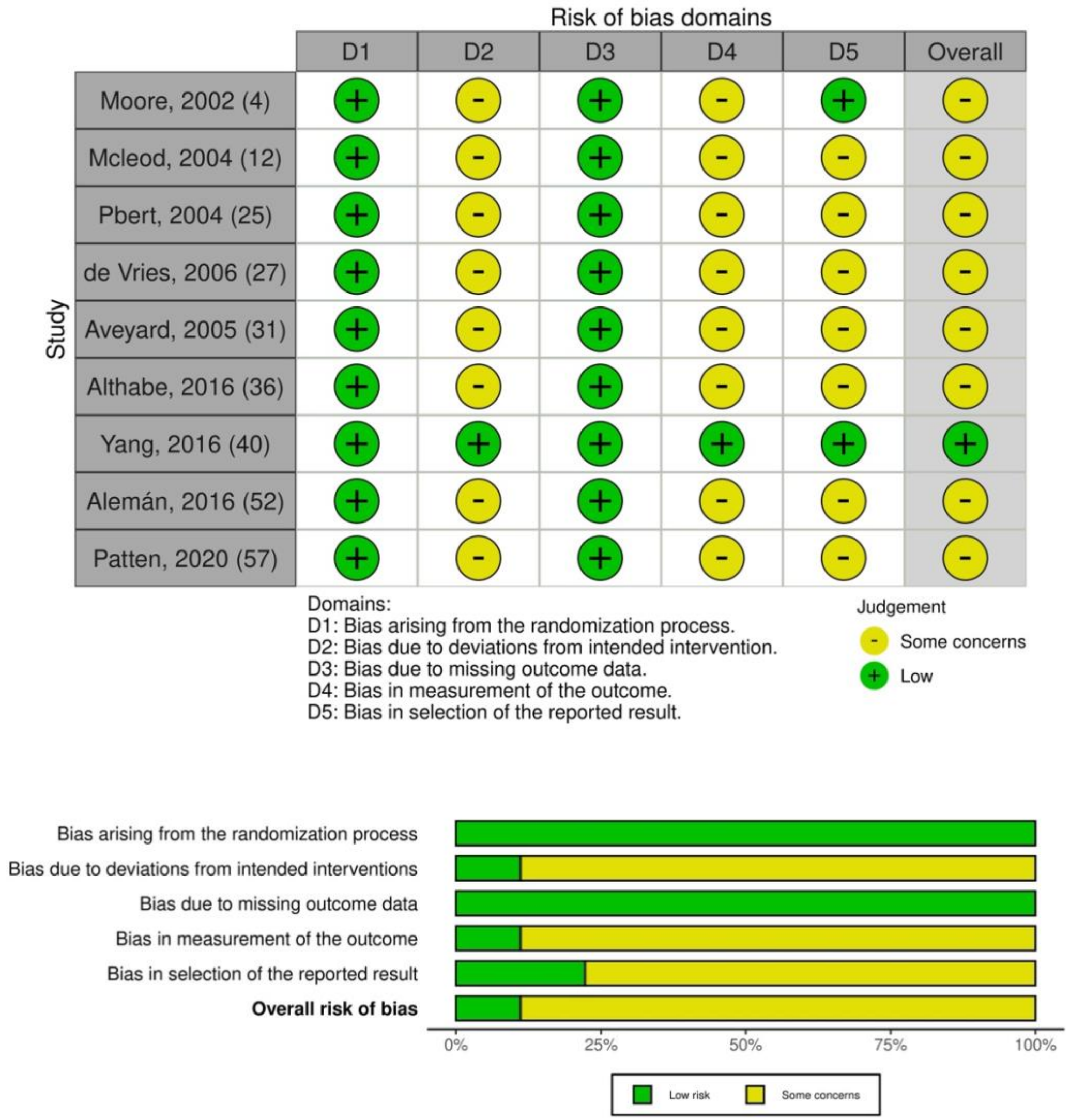

Table 4.3. Risk of bias assessment of non-randomized controlled trials[3]

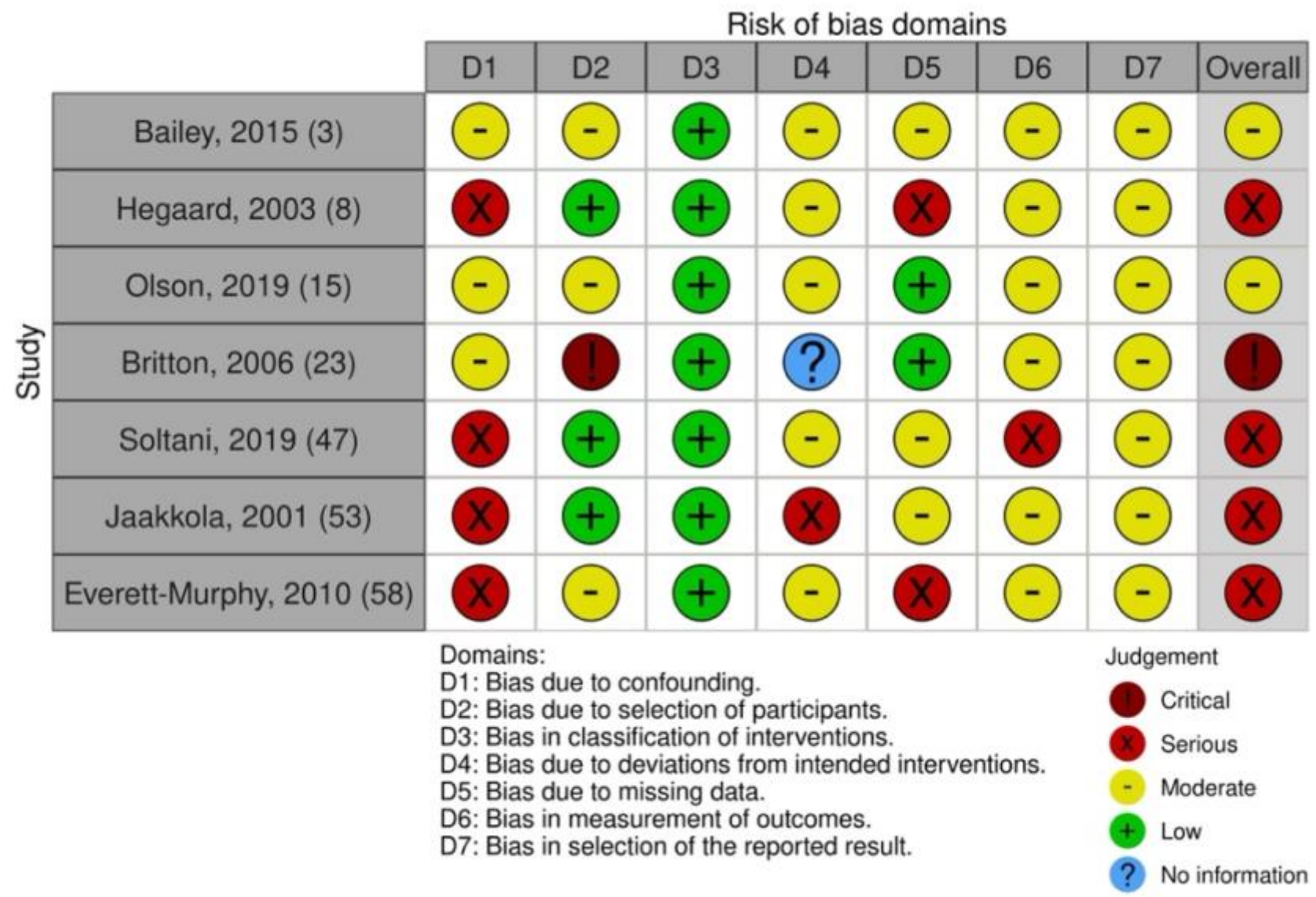

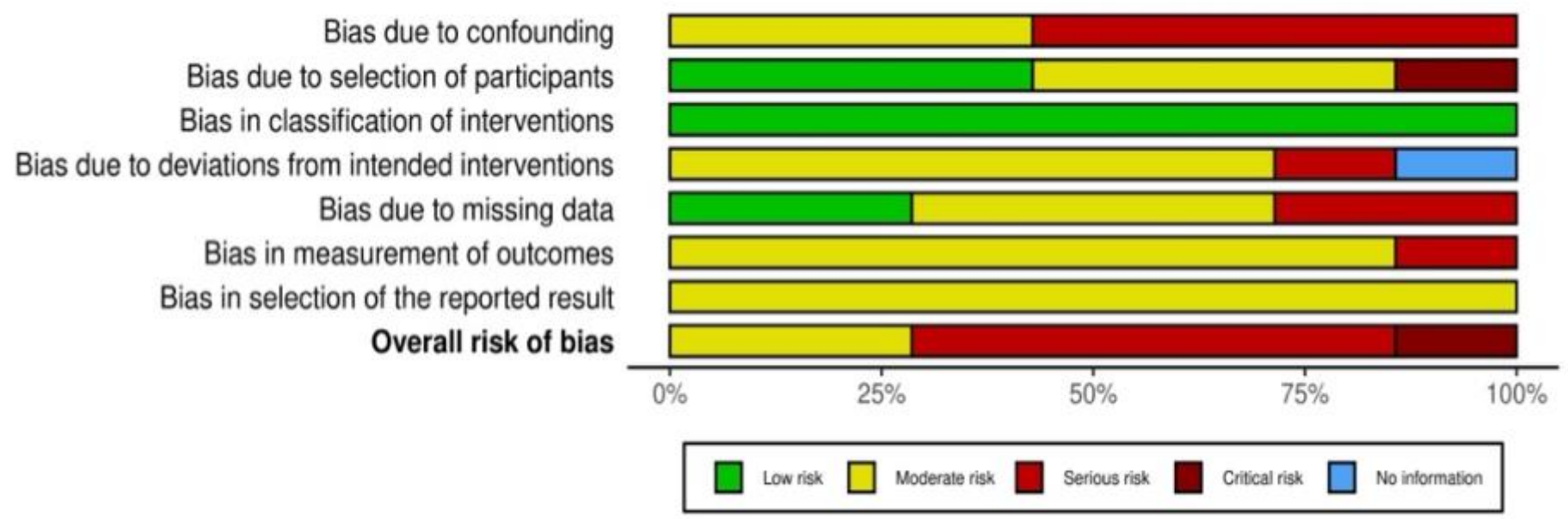

NOTES:

[1] Bias assessment of individual randomised controlled trials was conducted using the Cochrane Risk of Bias 2.0 (RoB 2.0) tool. Sterne JAC, Savović J, Page MJ, Elbers RG, Blencowe NS, Boutron I, Cates CJ, Cheng H-Y, Corbett MS, Eldridge SM, Hernán MA, Hopewell S, Hróbjartsson A, Junqueira DR, Jüni P, Kirkham JJ, Lasserson T, Li T, McAleenan A, Reeves BC, Shepperd S, Shrier I, Stewart LA, Tilling K, White IR, Whiting PF, Higgins JPT. RoB 2: a revised tool for assessing risk of bias in randomised trials. *BMJ* 2019; 366: l4898.

[2] Bias assessment of cluster randomised controlled trails was conducted using the Cochrane RoB 2 with Additional considerations for cluster-randomized trials (RoB 2.0 CRCT) tool. Sterne JAC, Savović J, Page MJ, Elbers RG, Blencowe NS, Boutron I, Cates CJ, Cheng H-Y, Corbett MS, Eldridge SM, Hernán MA, Hopewell S, Hróbjartsson A, Junqueira DR, Jüni P, Kirkham JJ, Lasserson T, Li T, McAleenan A, Reeves BC, Shepperd S, Shrier I, Stewart LA, Tilling K, White IR, Whiting PF, Higgins JPT. RoB 2: a revised tool for assessing risk of bias in randomised trials. *BMJ* 2019; 366: l4898.

[3] Bias assessment of non-randomised controlled trials was conducted using the Risk Of Bias In Non-randomized Studies of Interventions (ROBINS-I) tool. Sterne JAC, Hernán MA, Reeves BC, Savović J, Berkman ND, Viswanathan M, Henry D, Altman DG, Ansari MT, Boutron I, Carpenter JR, Chan AW, Churchill R, Deeks JJ, Hróbjartsson A, Kirkham J, Jüni P, Loke YK, Pigott TD, Ramsay CR, Regidor D, Rothstein HR, Sandhu L, Santaguida PL, Schünemann HJ, Shea B, Shrier I, Tugwell P, Turner L, Valentine JC, Waddington H, Waters E, Wells GA, Whiting PF, Higgins JPT. ROBINS-I: a tool for assessing risk of bias in non-randomized studies of interventions. BMJ 2016; 355; i4919; doi: 10.1136/bmj.i4919.

5. GENERAL CHARACTERISTICS OF INCLUDED STUDIES

Table 5.1. Characteristics of the studies included in the systematic review.

Author, Year, Reference	Study design	Target group	Vulnerability characteristics	Vulnerability approach	Father involvement
Eades, 2012 (1)<br>*Australia*	RCT	Aboriginal and Torres Strait Islander women attending antenatal clinics at specific Aboriginal community-controlled health services in urban communities in Queensland and Western Australia.	- Ethnic / racial minorities<br>-Socioeconomical disadvantages<br>- Financial insecurity	- Specialized training for vulnerability<br>- Involvement of local communities and reflecting cultural values	Active role
Panjari, M., 1999 (2)<br>*Australia*	RCT	Pregnant smokers attending antenatal care at the Royal Women's Hospital in Melbourne, Victoria.	- Socioeconomical disadvantages	- Mental health and emotional support	No role
Bailey, 2015 (3)<br>*United States*	Before-after study	Pregnant women in rural South-Central Appalachia, predominantly young (younger than 25 years old), Caucasian, with a low socioeconomic status qualifying for government medical insurance assistance (state Medicaid).	- Socioeconomical disadvantages<br>- Low educational level<br>- Young pregnancy	- Socioeconomic inclusiveness<br>- Mental health and emotional support	No role
Moore, 2002 (4)<br>*United Kingdom*	Cluster RCT	Pregnant women who smoked at the start of pregnancy, were aged 16 years or over, less than 17 weeks pregnant and attended their first antenatal appointment in the three participating trusts.	- Low educational level	Not mentioned	Active role

Polańska, 2004 (5) *Poland*	RCT	Pregnant women from maternity centres in Łódź, central Poland, including socially underprivileged women from an urban community.	- Socioeconomical disadvantages<br>- Low educational level<br>- Living alone or separated from partner: some participants	Not mentioned	No role
Tappin, 2005 (6) *United Kingdom*	RCT	Pregnant women who were regular smokers at antenatal booking in Glasgow.	- Socioeconomical disadvantages<br>- Low socioeconomical level<br>- Living alone or separated from partner: some participants	- Specialized training for vulnerability	No role
Mcdonnell, 2023 (7) *Ireland*	RCT	Pregnant women who smoke and are at less than 20 weeks' gestation attending a large urban maternity unit delivering over 8000 infants per year.	- Socioeconomical disadvantages<br>- Mental disorders (depression, anxiety)<br>- Living alone or separated from partner: some participants	- Mental health and emotional support	No role
Hegaard, 2003 (8) *Denmark*	Non-randomised controlled trial	Pregnant women who were daily smokers and paying their first prenatal visit to the Midwifery Center at a large university hospital.	- Low educational level: some participants had less than 12 years of schooling<br>- Alcohol use: some participants reported alcohol consumption	- Specialized training for vulnerability	No role
Mejdoubi, 2014 (9) *Netherlands*	RCT	Young pregnant women with low educational level living in 20 municipalities in the Netherlands.	- Ethnic/racial minorities<br>- Socioeconomical disadvantages<br>- Low educational level<br>- Young pregnancy<br>- Financial insecurity<br>- Alcohol use	- Specialized training for vulnerability	Passive role

Tappin, 2015 (10)<br>*United Kingdom*	RCT	Pregnant smokers in Glasgow, Scotland, aged 16 years or more, less than 24 weeks pregnant, residing in NHS Greater Glasgow and Clyde, and able to understand and speak English for telephone consent.	- Socioeconomical disadvantages<br>- Financial insecurity	- Socioeconomic inclusiveness	No role
Tappin, 2022 (11)<br>*United Kingdom*	RCT	Pregnant women smokers aged 16 years or older and less than 24 weeks' gestation.	- Socioeconomical disadvantages<br>- Young pregnancy	- Socioeconomic inclusiveness	No role
Mcleod, 2004 (12)<br>*New Zealand*	Cluster RCT	Pregnant women who smoke during pregnancy in New Zealand, with a focus on those in lower socio-economic groups and Maori women.	- Ethnic/ racial minorities<br>- Socioeconomical disadvantages<br>- Low educational level<br>- Financial insecurity	- Involvement of local communities and reflecting cultural values	Passive role
Hajek, 2001 (13)<br>*United Kingdom*	RCT	Pregnant women in the third month of pregnancy, including recent ex-smokers and current smokers, receiving care from midwives in hospital and community trusts.	- Socioeconomical disadvantages<br>- Low educational level<br>- Living alone or separated from partner: some participants	Not mentioned	Passive role
Dornelas, 2006 (14)<br>*United States*	RCT	Low-income, predominantly Hispanic, pregnant women in an urban prenatal clinic with a mean age of 26.1 years.	- Ethnic/racial minorities<br>- Socioeconomical disadvantages<br>- Low educational level<br>- Financial insecurity<br>- Living alone or separated from partner: some participants<br>- Depression	- Specialized training for vulnerability<br>- Mental health and emotional support	No role
Olson, 2019 (15)<br>*United States*	Non-randomised	Pregnant women who were smokers at their first prenatal visit from low-income populations in rural New Hampshire.	- Socioeconomical disadvantages<br>- Low educational level	- Socioeconomic inclusiveness	No role

	controlled trial		- Low socioeconomical level<br>- Financial insecurity		
Gielen, 1997 (16)<br>*United States*	RCT	Low-income, African-American pregnant women receiving care at an urban prenatal clinic in Baltimore, Maryland.	- Ethnic/ racial minorities<br>- Socioeconomical disadvantages<br>- Low educational level<br>- Financial insecurity	- Language and education considerations<br>- Involvement of local communities and reflecting cultural values	No role
Rigotti, 2006 (17)<br>*United States*	RCT	Pregnant smokers referred by prenatal providers and a managed care plan, predominantly daily smokers, aged 18 years or older, at 26 weeks or less of gestation, willing to consider altering their smoking during pregnancy, reachable by telephone, English-speaking, and expected to live in New England for the next year.	- Ethnic/ racial minorities<br>- Socioeconomical disadvantages<br>- Low educational level<br>- Financial insecurity<br>- Depression	- Specialized training for vulnerability	No role
Coleman, 2021 (18)<br>*United Kingdom*	RCT	Pregnant women who were at least 16 years old, up to 25 weeks' gestation, and smoked at least one daily cigarette, predominantly white with an average age of 27 years.	- Socioeconomical disadvantages: some participants	- Language and education considerations<br>- Socioeconomic inclusiveness	Passive role
Ershoff, 1999 (19)<br>*United States*	RCT	English-speaking pregnant women aged 18 years or older who self-reported as active smokers at their initial prenatal appointment.	- Ethnic/ racial minorities	- Language and education considerations	No role
Walsh, 1997 (20)<br>*Australia*	RCT	Pregnant women attending a public antenatal clinic in an urban setting in Australia.	- Low educational level: some participants	Not mentioned	No role

Stotts, 2002 (21)<br>*United States*	RCT	Resistant pregnant smokers receiving prenatal care at multispecialty clinics in the Houston and Dallas metropolitan areas who were members of group insurance plans.	- Ethnic/ racial minorities<br>- Low educational level	- Specialized training for vulnerability	Active role
Secker-Walker, 1994 (22)<br>*United States*	RCT	Pregnant women who smoked receiving prenatal care at the University of Vermont, including both private obstetricians/nurse-midwives and the Maternal-Infant Care (MIC) clinic for underinsured or uninsured women.	- Socioeconomical disadvantages<br>- Low educational level<br>- Financial insecurity	- Specialized training for vulnerability	No role
Britton, 2006 (23)<br>*United States*	Before-after study	Pregnant women who were smokers at the beginning of their pregnancies, living in rural communities, predominantly White, single, or separated, and with Medicaid insurance.	- Socioeconomical disadvantages<br>- Low educational level<br>- Living alone or separated from partner: some participants<br>- Financial insecurity	Not mentioned	Passive role
O'Connor, 1992 (24) *Canada*	RCT	Pregnant smokers attending antenatal clinics in a diverse socioeconomic and linguistic setting in Canada.	- Socioeconomical disadvantages<br>- Financial insecurity	- Language and education considerations<br>- Mental health and emotional support	No role
Pbert, 2004 (25)<br>*United States*	Cluster RCT	Pregnant and postpartum women receiving care at community health centres in the greater Boston area, including those utilizing prenatal and paediatric services and the Special Supplemental Nutrition Program for Women, Infants, and Children (WIC).	- Ethnic/ racial minorities<br>- Socioeconomical disadvantages<br>- Low educational level<br>- Low socioeconomical level<br>- Financial insecurity<br>- Living alone or separated from partner: some participants	- Language and education considerations<br>- Socioeconomic inclusiveness	No role

Luk, 2021 (26)<br>*China*	RCT	Male adults who were expectant fathers and smokers.	- Low educational level	- Language and education considerations<br>- Socioeconomic inclusiveness	Active role
de Vries, 2006 (27)<br>*Netherlands*	Cluster RCT	Pregnant women who smoke and receive prenatal care from midwives in the Netherlands.	- Low educational level<br>- Alcohol use	- Language and education considerations<br>- Specialized training for vulnerability<br>- Involvement of local communities and reflecting cultural values	Active role
Cummins, 2016 (28)<br>*United States*	RCT	Pregnant smokers in the first 27 weeks of gestation who called a state quit line for help with quitting, predominantly white with a mean age of 26.3 years and 66% having a high school education or less.	- Ethnic/racial minorities<br>- Socioeconomical disadvantages<br>- Low educational level<br>- Low socioeconomical level<br>- Financial insecurity<br>- Living alone or separated from partner	- Language and education considerations<br>- Specialized training for vulnerability	No role
Lowe, 1998 (29)<br>*Australia*	RCT	Pregnant women who smoke and present to the antenatal clinic of a metropolitan hospital in Brisbane.	- Socioeconomic disadvantages<br>- Low educational level	- Language and education considerations<br>- Involvement of local communities and reflecting cultural values	Passive role
Bullock, 2009 (30)<br>*United States*	RCT	Low-income rural pregnant women attending Women Infant and Children Nutritional Supplement (WIC) clinics in a Midwest state who reported smoking at least 1 cigarette per day, spoke English, were 18 years or older, and were less than 24 weeks gestation.	- Socioeconomical disadvantages<br>- Low educational level<br>- Low socioeconomical level<br>- Stress and depression	- Language and education considerations<br>- Specialized training for vulnerability<br>- Mental health and emotional support	No role

Aveyard, 2005 (31)<br>*United Kingdom*	Cluster RCT	Pregnant women who were smokers at booking for antenatal care and their partners.	- Socioeconomical disadvantages<br>- Low educational level<br>- Young pregnancy<br>- Low socioeconomical level	Not mentioned	Passive role
Abroms, 2017 (32)<br>*United States*	RCT	Pregnant smokers enrolled in the Text4baby program, aged ≥14 years, predominantly white, with Medicaid or Medicare insurance, with a high school degree or less as their highest level of education, and predominantly unemployed.	- Socioeconomical disadvantages<br>- Low educational level<br>- Low socioeconomical level<br>- Financial insecurity<br>- Living alone or separated from partner: some participants<br>- Substance abuse: Marijuana use<br>- Alcohol use<br>- Mental disorders (depression, anxiety)	- Language and education considerations<br>- Involvement of local communities and reflecting cultural values	No role
Mcbride, 2004 (33)<br>*United States*	RCT	Young pregnant women (mean age of 24) who were current smokers or recent quitters, living with an intimate partner, and willing to involve their partners in the study.	- Ethnic/ racial minorities<br>- Low educational level<br>- Young pregnancy	- Language and education considerations<br>- Specialized training for vulnerability<br>- Mental health and emotional support	Active role
Malchodi, 2003 (34)<br>*United States*	RCT	Predominantly Hispanic pregnant women who were low-income, smoked at least one cigarette per day before pregnancy, intended to carry the pregnancy to term, were less than 20 weeks' gestation, spoke English or Spanish, and were 18 years or older.	- Ethnic/ racial minorities<br>- Socioeconomical disadvantages<br>- Low educational level<br>- Low socioeconomical level<br>- Financial insecurity<br>- Living alone or separated from partner: some participants	- Language and education considerations<br>- Specialized training for vulnerability<br>- Involvement of local communities and reflecting cultural values	No role

Pollak, 2015 (35)<br>*United States*	RCT	Latino expectant fathers who smoked recruited through their pregnant partners from county health departments. Men who were at least 16 years old, had smoked at least 100 cigarettes in their lifetime, and were smoking in the past 30 days.	- Immigrants or migration background<br>- Ethnic/ racial minorities<br>- Low educational level<br>- Young pregnancy<br>- Financial insecurity	- Language and education considerations<br>- Involvement of local communities and reflecting cultural values	Active role
Althabe, 2016 (36)<br>*Argentina and Uruguay*	Cluster RCT	Pregnant women attending ANC clinics in public maternity hospitals in Buenos Aires, Argentina, and Montevideo, Uruguay, particularly those from deprived economic sectors.	- Socioeconomical disadvantages<br>- Young pregnancy	- Specialized training for vulnerability	No role
McBride, 2005 (37)<br>*United States*	RCT	Pregnant women who were less than 20 weeks pregnant and their male partners, predominantly Caucasian, married, and with some male partners being current smokers.	Not mentioned	Not mentioned	Active role
Cope, 2003 (38)<br>*United Kingdom*	RCT	Pregnant women attending three large inner-city hospital antenatal clinics in the UK.	Not mentioned	Not mentioned	No role
Pollak, 2020 (39)<br>*United States*	RCT	Pregnant women between 10 and 30 weeks pregnant who were current smokers, willing to quit, enrolled in prenatal care, aged 18 or older, and English speakers.	- Ethnic/ racial minorities<br>- Socioeconomical disadvantages<br>- Low educational level<br>- Financial insecurity	- Language and education considerations<br>- Socioeconomic inclusiveness	No role
Yang, 2016 (40)<br>*China*	Cluster RCT	Non-smoking pregnant women in China, specifically in Sichuan province, who had husbands who smoked and lived with them.	- Socioeconomical disadvantages<br>- Low educational level<br>- Low socioeconomical level<br>- Financial insecurity	Not mentioned	Active role

Marin-Gomez, 2019 (41) *Spain*	RCT	Pregnant women smokers above 18 years old attending Sexual and Reproductive Health Units during the first trimester of pregnancy in Catalonia, Spain.	- Low educational level	Not mentioned	No role
Xia, 2020 (42) *China*	RCT	Smoking expectant fathers who registered with their pregnant partners in China, with a mean age of 32 years, where approximately half were first-time expectant fathers and about two-thirds had completed tertiary education.	Not mentioned	Not mentioned	Active role
Loukopoulou, 2018 (43) *Greece*	RCT	Pregnant smokers in Athens, Greece who were randomly assigned to either a high-intensity smoking cessation intervention group or a low-intensity control group.	- Immigrants or migration background<br>- Socioeconomical disadvantages<br>- Low/ medium educational level<br>- Low socioeconomical level<br>- Financial insecurity: some unemployed	Not mentioned	No role
Keten, 2013 (44) *Turkey*	RCT	Pregnant women who smoke.	- Socioeconomical disadvantages<br>- Financial insecurity	Not mentioned	No role
Tsoh, 2010 (45) *United States*	RCT	Pregnant smokers regardless of their intention to quit smoking, English-speaking women 18 years or older, less than 26 weeks pregnant, receiving prenatal care at the participating clinics	- Ethnic/ racial minorities<br>- Socioeconomical disadvantages<br>- Low educational level<br>- Low socioeconomical level<br>- Living alone or separated from partner: some participants	- Language and education considerations	No role

			- Substance abuse: alcohol and illicit drug use		
Forinash, 2018 (46) *United States*	RCT	Pregnant women receiving obstetric care at the maternal foetal care centre, who were at least 18 years of age and in the preparation stage of change for smoking cessation.	- Ethnic/ racial minorities<br>- Socioeconomical disadvantages<br>- Living alone or separated from partner: some participants<br>- Mental disorders (depression, anxiety)<br>- Alcohol use	- Socioeconomic inclusiveness	No role
Soltani, 2019 (47) *Iran*	Before-after study	Pregnant women exposed to second-hand smoke at home.	- Low educational level	Not mentioned	Active role
Albrecht, 1998 (48) *United States*	RCT	Pregnant adolescents who smoke.	- Ethnic/ racial minorities<br>- Young pregnancy	Not mentioned	No role
Stanton, 2004 (49) *Australia*	RCT	Men who smoked when their partners booked into the public antenatal clinic at two large Brisbane metropolitan hospitals, aged 16 to 56 years, primarily Australian, with a mix of unskilled, semiskilled, and skilled workers.	- Immigrants or migration background<br>- Ethnic/ racial minorities<br>- Socioeconomical disadvantages<br>- Low educational level<br>- Low socioeconomical level	Not mentioned	Active role
Hjalmarson, 1991 (50) *Sweden*	RCT	Pregnant smokers attending public health maternity clinics in Gothenburg for their first appointment, with specific inclusion criteria of daily smoking, gestational age of less than 12 weeks, and ability to understand Swedish.	Not mentioned	Not mentioned	No role

Windsor, 1985 (51)<br>*United States*	RCT	Pregnant smokers attending public health maternity clinics, excluding those who entered care after 32 weeks.	- Socioeconomical disadvantages<br>- Low socioeconomical level	- Language and education considerations<br>- Involvement of local communities and reflecting cultural values	No role
Alemán, 2016 (52)<br>*Argentina and Uruguay*	Cluster RCT	Women who were non-smokers or smokers who quit during pregnancy.	- Socioeconomical disadvantages<br>- Young pregnancy	Not mentioned	No role
Jaakkola, 2001 (53)<br>*Finland*	Non-randomised controlled trial	Women who were smokers at their first visit to maternity health clinics and who gave birth in the two birth clinics in The Kymi Hospital District.	Not mentioned	Not mentioned	Active role
Price, 1991 (54)<br>*United States*	RCT	Indigent inner-city pregnant women of low socioeconomic status, with low education levels, typically lower income, unmarried, and young (mean age of 22.6 years).	- Socioeconomical disadvantages<br>- Low educational level<br>- Young pregnancy<br>- Low socioeconomical level<br>- Financial insecurity<br>- Living alone or separated from partner	- Language and education considerations<br>- Involvement of local communities and reflecting cultural values	No role
Higgins, 2022 (55)<br>*United States*	RCT	Pregnant and newly postpartum women who were smokers, with a focus on those still smoking at the initiation of prenatal care, mostly young, with lower levels of education, and of Non-Latino White race/ethnicity.	- Socioeconomical disadvantages<br>- Low educational level<br>- Substance abuse<br>- Mental disorders (depression, anxiety)	- Socioeconomic inclusiveness	No role
Herbert, 2011 (56)<br>*Canada*	RCT	Families with children aged below 5 years who were exposed to environmental tobacco smoke in the home.	- Socioeconomical disadvantages<br>- Low educational level<br>- Low socioeconomical level	- Language and education considerations<br>- Specialized training for vulnerability	Active role

			- Financial insecurity<br>- Living alone or separated from partner: some participants	- Mental health and emotional support<br>- Involvement of local communities and reflecting cultural values	
Patten, 2020 (57)<br>*United States*	Cluster RCT	The target group of the study was pregnant Alaska Native (AN) women in rural western Alaska, with a mean age of 25.8 years, predominantly married or partnered, and at various stages of gestation.	- Ethnic/ racial minorities<br>- Socioeconomical disadvantages<br>- Low educational level<br>- Young pregnancy<br>- Low socioeconomical level	- Language and education considerations<br>-Involvement of local communities and reflecting cultural values	No role
Everett-Murphy, 2010 (58)<br>*South Africa*	Before-after study	Pregnant women of low socio-economic status, of mixed ethnic descent, living in Cape Town, with a significant percentage being teenagers between 14 and 19 years old. The study focused on pregnant smokers attending public sector antenatal clinics in South Africa.	- Ethnic minorities<br>- Socioeconomical disadvantages<br>- Low educational level<br>- Young pregnancy<br>- Low socioeconomical level<br>- Financial insecurity<br>- Some living alone or separated from partner	- Language and education considerations<br>- Socioeconomic inclusiveness<br>- Specialized training for vulnerability<br>- Involvement of local communities and reflecting cultural values	Active role

6. DETAILED CHARACTERISTICS OF INCLUDED STUDIES

Table 6.1. Detailed intervention description

Author, Year, Reference	Name of intervention	Intervention group summary of intervention	Control group summary of intervention	Health professional delivering intervention	Duration and frequency of intervention	Role of technology
Eades, 2012 (1)	Not mentioned	The intervention group involved tailored advice and support to quit smoking delivered by a general practitioner and other health care workers, incorporating culturally sensitive elements and specific strategies such as advising women to quit "cold turkey," involving significant others in the quitting process, providing evidence-based communication, offering nicotine replacement therapy after two failed quit attempts, and scheduling booster support sessions.	The control group received general advice from their GP about quitting smoking based on existing brief intervention guidelines.	GP<br>Advisor	The intervention involved tailored advice and support to quit smoking at the first antenatal visit, engagement of the woman's partner and other adults in supporting quit attempts, and follow-up visits scheduled at 3-5 days and 7-10 days after the first antenatal visit. Training was provided on-site at each clinic at baseline and throughout the intervention period.	Use of mobile phones to send text message reminders to participants about their clinic appointments.
Panjari, M., 1999 (2)	Personalized smoking cessation intervention	The intervention group received personalized smoking cessation counselling sessions based on cognitive therapy techniques, including discussions on risks of smoking in pregnancy, identification of smoking cues, benefits of quitting, goal setting, and emotional support. Follow-up sessions were provided at specific gestational weeks to aid in smoking cessation efforts.	The control group received standard antenatal care with the distribution of a Quit Victoria pamphlet on Smoking and Pregnancy.	Midwife	The intervention included counselling on 4 occasions by the same midwife trained in smoking cessation techniques. The first session lasted about 25 minutes and subsequent sessions were 5-10 minutes long. Follow-up sessions were offered at around 16-20, 24, and 28 weeks' gestation.	Not mentioned

Bailey, 2015 (3)	Tennessee Intervention for Pregnant Smokers (TIPS) program	The intervention group in the study consisted of pregnant women recruited over a 4-year period who were current cigarette smokers. The intervention aimed to help pregnant women quit smoking by the end of the second trimester using the 5A's model and personalized assistance tailored to individual needs.	The control group received usual care during their prenatal visits, which included an initial assessment of smoking status, brief advice to quit, tips for smoking cessation, referrals for assistance, and checking about smoking at subsequent visits.	Advisor	The duration of the initial intervention sessions ranged from 15 to 30 minutes, while subsequent sessions lasted from 10 to 20 minutes. Additional intervention sessions were provided during three prenatal visits, which typically occurred monthly in the first and second trimester of pregnancy. Fidelity checks were conducted to ensure the intervention was tailored to each patient's needs.	Information was provided about online resources and referrals to the state-administered smoking quit line.
Moore, 2002 (4)	Stop for Good	The intervention in the intervention group consisted of a series of five self-help booklets aimed at increasing motivation for quitting smoking and teaching strategies for cessation and relapse prevention. The first booklet was given in person by a midwife during antenatal care, with the remaining four booklets sent by mail directly to the women.	The control group received usual care from midwives during the first antenatal appointment without the introduction of self-help booklets on smoking cessation. The delivery of normal care varied among midwives in this group.	Midwife	The intervention consisted of a series of five self-help booklets, with the first booklet given in person by a midwife and the remaining four mailed weekly to the participants. Midwives were instructed to spend about five minutes introducing the first booklet, but the actual time varied.	Not mentioned
Polańska, 2004 (5)	Not mentioned	The intervention group received a smoking cessation program consisting of midwife visits, assessment of smoking addiction, declaration to quit smoking, follow-up visits, and support for maintaining smoking abstinence. Spontaneous quitters were also included in the intervention and received support to maintain smoking abstinence.	The control group received standard written information about the health risks of maternal smoking to the foetus, but did not receive the midwife-assisted smoking cessation intervention provided to the intervention group.	Midwife	The intervention program consisted of four midwife visits during pregnancy and one post-delivery visit, with additional visits offered if needed.	Not mentioned

Tappin, 2005 (6)	Not mentioned	The intervention group received motivational interviewing at home, with additional home visits and multiple contact attempts by midwives.	The control group received standard health promotion information but did not receive motivational interviewing at home or additional home visits from the study midwife.	Midwife	The duration of each intervention session ranged from about 30 minutes, with women receiving two to five additional home visits. The median intervention time was 56 minutes, with a range of 9 to 219 minutes. The intervention frequency included multiple attempts to contact women, resulting in varying numbers of interviews per woman.	Not mentioned
Mcdonnell, 2023 (7)	Smoking cessation Through Optimization of clinical care in Pregnancy (STOP clinic)	The intervention group in the study consisted of pregnant women attending the STOP antenatal clinic, where they received specialized care for smoking cessation including behavioural interventions, scheduled follow-up sessions, and access to nicotine replacement therapy.	The control group in the study received usual care with basic smoking cessation advice at the initial prenatal visit but did not have access to the specialized antenatal care and smoking cessation intervention provided in the STOP clinic. The smoking cessation rate was significantly lower in the control group compared to the intervention group.	Midwife<br>OB-GYN	The duration and frequency of the intervention sessions included follow-up assessments at 1 week, 4 weeks, 3 months, and 6 months post the quit date, with each assessment involving 1-on-1 counselling lasting approximately 10 minutes. Nicotine replacement therapy was offered if smoking continued at 4 weeks post the quit date. Smoking status was recorded at each visit using self-report and measurement of exhaled breath CO. The initial behavioural intervention was conducted by the smoking cessation practitioner, with the quit date typically set in the second trimester.	Not mentioned
Hegaard, 2003 (8)	Not mentioned	The intervention group received initial individual smoking cessation counselling, joined a smoking cessation program with nicotine replacement therapy, participated in a cognitive behaviour modification	The control group in the study received standard counselling from a midwife, routine information about the risks of smoking during pregnancy, and general advice on smoking cessation or reduction.	Midwife	The intervention program consisted of an initial extended visit, followed by a 14-week program with nine appointments, including preparation for quitting and maintenance sessions. Group	Not mentioned

		program, attended nine appointments over 14 weeks, and used nicotine replacement therapy with dosage regulated based on nicotine dependence.			sessions lasted 90 minutes, and individual sessions lasted 15-30 minutes. Nicotine replacement therapy was offered as part of the program.	
Mejdoubi, 2014 (9)	Voorzorg programme	The intervention group received the VoorZorg program, which included home visits by specialized nurses focusing on smoking cessation using the V-MIS program and promoting breastfeeding. Nurses also educated mothers on the negative health effects of smoking near the baby.	The control group received usual care provided by Dutch Youth Health Care Organizations, including maternal health care by midwives or obstetricians, health education, physical examinations, monitoring of foetal development, registration in a well-baby clinic for newborns, and support for parents.	Nurse	The VoorZorg program consisted of approximately 40-60 home visits by specialized nurses from pregnancy until two years after birth, with women receiving around 20 home visits during the period of the study, from pregnancy until six months postpartum.	Not mentioned
Tappin, 2015 (10)	Up to £400 of shopping vouchers added to routine specialist pregnancy NHS 'Stop Smoking Services'	The intervention group received routine specialist pregnancy support along with financial incentives in the form of shopping vouchers for engaging with stop smoking services or for quitting during pregnancy. The incentives included multiple vouchers at different stages of smoking cessation, with close monitoring of smoking status through various tests.	The control group was offered routine specialist pregnancy support by the stop smoking services without any financial incentives for smoking cessation.	Research Team Advisor	The intervention involved offering financial incentives (shopping vouchers) to pregnant smokers to help them quit smoking, with participants in the incentives group receiving vouchers at different stages of their smoking cessation journey during pregnancy. The specific duration and frequency of intervention sessions or program sessions are not explicitly mentioned in terms of specific session numbers or intervals.	Not mentioned
Tappin, 2022 (11)	Lovetoshop financial voucher incentives	The intervention group was offered financial voucher incentives up to £400 at four different time points to support smoking cessation during pregnancy, in addition to the	The control group received standard stop smoking services including counselling and free nicotine replacement therapy, without any financial incentives.	Midwife Research Team	The duration and frequency of the intervention involved offering financial incentives at four different time points: £50 voucher initially, followed by additional vouchers at different	Not mentioned

		usual support from stop smoking services.			stages based on smoking cessation progress.	
Mcleod, 2004 (12)	Programme of education and support for smoking cessation or reduction, programme of education and support for breast feeding	The intervention group included a smoking cessation intervention using brief intervention and motivational interviewing, supported by resources like a flipchart and a videotape, along with training sessions by a smoking cessation counsellor. The breast-feeding intervention focused on increasing the duration of full breastfeeding through practical information, training, and support sessions with a lactation consultant.	The control group received usual care from midwives, with regular phone contact from the research team for support and communication.	Midwife	The duration of the intervention sessions varied, with a four-hour training session conducted for smoking education and support, along with additional problem-solving sessions. The breast-feeding intervention also included training sessions with a lactation consultant and problem-solving sessions.	Not mentioned
Hajek, 2001 (13)	Not mentioned	The intervention in the intervention group included advice from midwives, written materials tailored for different smoker groups, a quiz to reinforce understanding, commitment to a quit date, and the option for mutual support through a 'Buddy Card'.	The control group in the study received standard care, which typically included access to anti-smoking leaflets and the midwives' usual procedures with smokers and recent ex-smokers.	Midwife	The duration of the intervention was approximately 15 minutes on average, and it was described as a 'one-off' intervention.	Not mentioned
Dornelas, 2006 (14)	Not mentioned	The intervention group received a 90-minute psychotherapy session at the clinic, followed by bi-monthly prenatal telephone calls during pregnancy and monthly calls after delivery, aiming to assist women in recognizing the link between psychological distress and smoking during pregnancy.	The control group received usual care consisting of standard smoking cessation treatment guidelines implemented by the health care provider, training of Ob-GYN residents and nurses in smoking cessation during pregnancy, provision of an educational booklet, and documentation of advice to quit smoking in the prenatal record.	Nurse<br>OB-GYN<br>Advisor	The intervention consisted of one 90-minute psychotherapy session at the clinic, followed by bi-monthly prenatal telephone calls during pregnancy and monthly telephone calls after delivery. The study coordinator made three proactive telephone calls to reschedule missed counselling appointments. The average number of completed	Not mentioned

					telephone calls per patient was 2.6.	
Olson, 2019 (15)	Smoke-free moms	The intervention group received 5A's smoking counselling and financial incentives in the form of gift cards for smoking cessation confirmed by negative urinary cotinine tests.	The control group intervention involved providing control subjects with $5 for urine testing at each visit without informing them of the results.	Nurse<br>Midwife<br>Advisor	The duration and frequency of the intervention involved providing financial incentives at each prenatal visit for smoking cessation, with an average cost of $148 per woman who had confirmed smoking cessation through cotinine testing.	Not mentioned
Gielen, 1997 (16)	Smoke-free moms project	The intervention group received a "Pregnant Woman's Guide to Quit Smoking" with a sixth-grade reading level at their first prenatal visit, developed based on a needs assessment and incorporating constructs from a PRECEDE/PROCEED diagnosis and Social Learning Theory. The guide's appeal was tested through focus groups, and at delivery, the counsellor provided educational materials and brief counselling on a new guide for either cessation or relapse prevention based on the woman's smoking status. Postpartum, mothers who had not quit during pregnancy received a guide with both cessation and relapse prevention content, while those who had quit during pregnancy received a guide with only relapse prevention content.	The control group received routine clinic advice about smoking cessation and relapse prevention, including a brief discussion from a nurse, a recommendation to quit, and pamphlets from voluntary agencies.	Nurse	The duration of the intervention included a 15-minute one-to-one counselling session with a peer health counsellor and the frequency involved receiving components of the intervention at each prenatal visit. Compliance with the intervention was good, with almost all women in the E group receiving and using the printed materials.	Not mentioned
Rigotti, 2006 (17)	Proactive pregnancy-tailored telephone counselling for smoking cessation	The intervention group received proactive pregnancy-tailored telephone counselling with a dedicated counsellor, lasting up	The control group received a single 5-minute counselling call as part of the "best practice" intervention, along with a	Advisor	The intervention consisted of a series of telephone calls throughout pregnancy and for 2 months postpartum, with each	Not mentioned

		to 90 minutes during pregnancy and up to 15 minutes postpartum. The counselling was tailored to individual readiness to quit and interest in pregnancy and health-related topics, drawing on various psychological theories and models.	validated pregnancy-tailored smoking cessation booklet and referral to the Massachusetts telephone quit line for additional support.		subject receiving up to 90 minutes of counselling during pregnancy and up to 15 minutes of counselling postpartum. The number of calls varied based on the subject's needs, with a mean of 5 calls during pregnancy. The content included setting goals, making a cessation plan, cognitive strategies for readiness to change, and cognitive-behavioural cessation techniques.	
Coleman, 2021 (18)	Miquit	The intervention group received the MiQuit program, which is a tailored, self-help, text message stop smoking program for pregnancy. It consists of 12 weeks of personalized smoking cessation text messages aimed at inducing and aiding cessation for pregnant women who smoke. The program encourages quit attempts and helps women succeed in quitting.	The control group received usual care within the NHS antenatal clinics, including access to any smoking cessation information, advice, or support available to them, along with the 'Baby on the way, quit today' smoking cessation booklet.	Research Team	The intervention duration is 12 weeks, consisting of tailored smoking cessation text messages with personalized content and varying message frequency.	Tailored text message program called MiQuit, designed to support pregnant women in smoking cessation by sending personalized messages and providing interactive features for on-demand support. Additionally, some participants used other technological supports such as additional text message services and mobile phone apps related to smoking cessation.

Ershoff, 1999 (19)	Not mentioned	Intervention 1: Booklet only provided a tailored self-help booklet for pregnant smokers; Intervention 2: IVR offered access to a computerized interactive telephone support system; Intervention 3: MI involved telephone counselling using motivational interviewing techniques.	The control group received a self-help booklet called Living Smoke-Free tailored to pregnant smokers' lifestyle and readiness to change, without any additional resource-intensive interventions. Smoking-related advice during prenatal visits was not standardized.	Nurse<br>Research Team	The self-help booklet intervention was a one-time provision, the IVR program allowed 24/7 access with approximately five-minute calls, and the MI counselling involved between four and six weekly calls lasting 10 to 15 minutes each.	Use of a computerized telephone cessation program based on interactive voice response (IVR) technology.
Walsh, 1997 (20)	Structured, cognitive-behavioural smoking cessation program	The intervention group received a comprehensive smoking cessation program at their first visit to the antenatal clinic, including doctor advice, a 14-minute videotape, midwife counselling, self-help manual teaching, and confectionary gum packets.	The control group received standard smoking advice from the doctor and midwife at the first visit, along with a package of anti-smoking materials that were not specifically designed for pregnant women.	Midwife<br>GP	The duration of the intervention sessions varied from 2 to 14 minutes for different components, with midwives spending more time than doctors. The frequency included sessions at the first visit, second visit, and the 34-to 36-week visit.	Not mentioned
Stotts, 2002 (21)	One-to-One	The intervention group received personalized feedback letters and two telephone counselling calls using Motivational Interviewing strategies based on the stages of change model.	The control group received brief counselling and self-help booklets at the first prenatal visit, followed by weekly booklets, without the personalized feedback letter or Motivational Interviewing calls.	Research Team<br>Advisor	The intervention included a mix of face-to-face and telephone components, with counselling sessions lasting 3-30 minutes and feedback letters being sent at various intervals. Additional face-to-face counselling sessions were recommended to improve effectiveness.	Not mentioned
Secker-Walker, 1994 (22)	Individualized smoking cessation counselling	The intervention group received individualized smoking cessation counselling from trained counsellors in addition to the usual care provided by obstetricians or nurse-midwives. The counselling sessions followed a defined protocol and included discussions on concerns about smoking and pregnancy, health benefits of	The control group received the usual care, which consisted of standard advice about smoking provided by obstetricians or nurse-midwives. The content and nature of this advice were assessed through surveys during the study.	Nurse<br>Midwife<br>OB-GYN	The duration and frequency of the intervention sessions varied, with individual counselling provided at the first, second, and third prenatal visits, as well as at the 36-week visit and the 6-weeks-postpartum visit. The average duration of these sessions ranged from 11 to 18 minutes.	Not mentioned

		quitting, problem-solving, decision-making about quitting, formulating a quitting plan, rehearsing skills, providing educational materials, and addressing the effects of parental smoking on the child's health during follow-up visits.				
Britton, 2006 (23)	Smoke Free Baby & Me program	The intervention group in the study received the Smoke Free Baby & Me program, which included structured assessments, tailored health messages, readiness to quit evaluations, and provision of materials like a calendar for documenting smoking behaviour.	The control group received routine prenatal care without any specific smoking cessation intervention, but assessments for tobacco use, urine samples for cotinine analysis, and documentation of demographic and smoking-related behaviours were conducted.	Nurse	The intervention sessions or program lasted from November 1999 to August 2003, with participants in the control group enrolled for 15 months and those in the experimental group for 17 months. The intervention was implemented by nurse providers at four different points during the perinatal period. The intervention sessions occurred at least four times.	Not mentioned
O'Connor, 1992 (24)	Windsor's self-help smoking cessation program	The intervention group involved offering pregnant smokers the option to learn Windsor's self-help smoking cessation program during the first antenatal visit, provided on an individual basis by a public health nurse. Follow-up contact was made with a high percentage of subjects, and motivation levels varied among participants.	The control group received a brief verbal explanation of the hazards of smoking during pregnancy and a pamphlet inviting them to a 2-hour group cessation class using Windsor's self-help cessation program.	Nurse	The duration of the intervention sessions was approximately 20 minutes per individual, and the frequency was once during the first antenatal visit.	Not mentioned
Pbert, 2004 (25)	Quit together (qt)	The intervention in the intervention group involved existing healthcare providers delivering smoking treatment based on national clinical practice guidelines tailored to the woman's stage of change, supported by an office practice	The control group did not receive any specific smoking cessation training or intervention. Providers in the control group did not follow the structured intervention protocol followed by the intervention group.	Nurse<br>OB-GYN<br>Advisor	The duration and frequency of the intervention involved provider training based on national clinical practice guidelines, tailored to the woman's stage of change, and delivered through obstetric, paediatric, and WIC providers.	Not mentioned

		management system and program boards for coordination. Providers were trained to deliver clear quit messages, increase self-efficacy in quitting, offer assistance, and elicit a commitment to quit or maintain abstinence.			An office practice management system was used to routinely screen for smoking status and prompt providers to intervene, along with the establishment of program boards for coordination.	
Luk, 2021 (26)	Brief advice, nicotine replacement therapy sampling, and active referral (BANSAR)	The intervention group received brief cessation advice, a 1-week free sample of nicotine replacement therapy (NRT), and active referral to a community-based smoking cessation service.	The control group in the study received brief cessation advice along with a leaflet on the hazards of perinatal exposure to tobacco smoke and a toll-free quit line telephone number. They did not receive the additional components provided to the intervention group, such as a 1-week sample of NRT and active referral to a smoking cessation service.	Research Team	The duration of the intervention sessions in the study involved offering a 1-week sample of NRT, providing information about smoking cessation services, referral to a smoking cessation service, and two telephone boosters within the first month after enrolment. The median time spent on recruitment, data collection, and intervention delivery was approximately 12 minutes.	Technology played a role in delivering the intervention through the use of a QR code for online registration, telecommunication for referrals and follow-up support, and a toll-free quit line telephone number for the control group.
de Vries, 2006 (27)	MIS smoking cessation protocol	The intervention group received brief health counselling, self-help materials on smoking cessation during pregnancy and early postpartum, a partner booklet on non-smoking, a video raising awareness about the consequences of smoking during pregnancy, and a self-help guide adapted for pregnant women. The partner booklet aimed to emphasize the importance of the partner's commitment to the pregnancy, the benefits of quitting together for the health of the baby,	The control group received usual care and a general folder from the Dutch Smoking and Health Foundation, without any specific counselling or intervention for smoking cessation.	Midwife	The intervention sessions lasted approximately 10 minutes during midwife consultations, with women in the experimental group receiving the intervention twice during their pregnancy. The intervention included a video, a self-help manual, a partner booklet, and health counselling by midwives.	Not mentioned

		father, and mother, and provided suggestions on how to quit smoking.				
Cummins, 2016 (28)	Pregnancy-specific counselling protocol	The intervention group received pregnancy-specific telephone counselling consisting of nine sessions covering various pregnancy-specific topics to support quitting and maintaining abstinence during pregnancy and postpartum.	The control group received self-help materials only, including information from the American Cancer Society and additional tips for quitting while pregnant.	Research Team Advisor	The intervention consisted of nine counselling sessions, including a prequit call, follow-up calls, a pre-birth call, and post-birth follow-up sessions. The counselling was provided by twenty-one staff members who received training on the pregnancy-specific protocol.	Not mentioned
Lowe, 1998 (29)	Healthy 2	The intervention group received a self-help booklet for smoking cessation, a motivational counselling session, and a magazine-style booklet called 'Healthy 2' focusing on pregnancy and smoking cessation.	The control group in the study received only the caution against smoking given by the midwife, without any additional intervention or materials.	Midwife	The intervention involved daily activities for nicotine withdrawal, psychological aspects of smoking, counselling sessions with a flip chart demonstration, and women were encouraged to use the self-help booklet and refer back to the magazine-style booklet for reference.	Not mentioned
Bullock, 2009 (30)	Baby BEEP	The intervention group received weekly telephone calls throughout pregnancy, along with 24/7 beeper access for additional support, based on a definition emphasizing non-judgmental listening, discussing pregnancy needs, providing information when requested, and making referrals to other professionals when needed, with the aim of decreasing stress and depression in pregnant women.	The control group received the Quit Smoking for Good pamphlet from the American Heart Association and did not receive the weekly telephone calls or 24-hour access to a nurse for social support like the intervention groups. They were also part of the monthly saliva sample collection and interviews during late pregnancy and post-delivery.	Nurse	The intervention consisted of weekly telephone calls lasting on average 13 minutes, with additional time spent addressing on-call pages. The mean time for a call ranged from 9.5 to 17.5 minutes.	Not mentioned
Aveyard, 2005 (31)	Pro-change programme for a healthy pregnancy	The intervention group received training on the Transtheoretical Model, stage-based self-help manuals for different stages of	The control group received standard smoking cessation advice from midwives, which included providing a leaflet	Midwife	The duration of the intervention sessions varied across arms, with Arm A having half a day's training, Arm B having 2 1/2	Use of an interactive computer program installed on a

		change, and a computer program for assessing and providing feedback on participants' progress in smoking cessation during pregnancy.	titled "Thinking about Stopping" that contained information on why women should stop smoking and how to do so. The intervention did not directly address partner quitting.		days of training along with self-help manuals and assessments, and Arm C having similar training to Arm B along with a computer program at specific time points during pregnancy.	laptop, which provided questions to assess the participant's stage of change, onscreen and audio feedback, and strategies for moving through stages of change. This program was used at multiple points during the pregnancy and postpartum period and required additional visits for completion.
Abroms, 2017 (32)	Quit4baby	The intervention group received the Quit4baby program, which was developed based on the Social Cognitive Theory and Clinical Practice Guidelines for pregnancy smoking cessation. The program included messages aimed at improving self-efficacy, describing outcomes of quitting, increasing social support, and enhancing behavioural capability for quitting. Messages were timed around enrolment, quit date, and baby's due date, with additional postpartum relapse prevention messages. Participants could also request additional support by texting specific keywords.	Participants in the control group received only the Text4baby program, without the additional Quit4baby smoking cessation text messaging program.	Research Team	The Quit4baby intervention program sends between one and eight messages per day, with the highest frequency around the quit date and tapering over time. The program continues for 3 months, with monthly surveys for 6 months, and includes postpartum relapse prevention messages. Participants were enrolled in Quit4baby for an average of 129.08 days, starting about 7.84 days before their quit date.	The role of technology in this study is to deliver a smoking cessation intervention via an interactive and intensive text messaging program called Quit4baby, which is added to an existing health text messaging program called Text4baby. The intervention involves sending multiple text messages per day

						to pregnant smokers, with interactive features that allow participants to receive additional support by texting keywords to the program.
Mcbride, 2004 (33)	Usual care (UC), woman-only (WO), partner-assisted (PA)	The intervention group received a partner-assisted (PA) intervention that aimed to improve couple communication about support behaviours, assist partners in developing alternatives to negative behaviours, prompt couples to plan for high-risk situations, and encourage partner smoking cessation. Partners also received six separate counselling calls guided by motivational interviewing techniques.	The control group received provider advice, a self-help guide, a relapse-prevention kit, and six counselling calls conducted by a health advisor using motivational interviewing techniques.	Advisor	The duration of the intervention was six counselling calls, with three calls during pregnancy and three calls postpartum.	Not mentioned
Malchodi, 2003 (34)	Peer-led smoking cessation program	The intervention group in the study received smoking cessation peer counselling from clinic health care providers and lay community health outreach workers who were selected based on specific criteria. Peer counsellors received standardized training in smoking cessation and were instructed to have eight client contacts with each participant to support and reinforce quit smoking messages.	The control group received usual care for smoking cessation provided by clinic staff physicians, residents, nurse practitioners, and nurses. The usual care included standardized training for smoking cessation using specific guidelines, delivering a strong quit message, discussing risks of smoking during pregnancy, distributing educational materials, assessing readiness to quit, providing counselling, and documenting counselling at each prenatal visit.	Nurse<br>GP<br>Advisor	The duration of the intervention involved two standardized training sessions for peer counsellors, one lasting 3 hours and the other 2 hours. Peer counsellors were instructed to have eight client contacts with each participant in the experimental group. They were also required to document details of each visit.	Not mentioned

Pollak, 2015 (35)	No lo deje para mañana deje de fumar hoy: Guía para Dejar de Fumar	The intervention group received a combination of written materials, nicotine replacement therapy (NRT), and a series of counselling sessions during pregnancy and postpartum to promote smoking cessation among expectant Latino fathers. The counselling sessions focused on emotional triggers related to family, health risks of smoking, setting goals for quitting, addressing barriers to quitting, improving communication skills, and promoting healthy behaviours for both partners.	The control group received written materials plus nicotine replacement therapy (NRT) without additional couple-based counselling.	Research Team	The intervention included three counselling sessions during pregnancy (one face-to-face and two via phone) and three postpartum (one face-to-face and two via phone). The first session lasted approximately two hours, subsequent sessions were around 15 minutes, and postpartum counselling included a phone session at 4 weeks, a face-to-face session at 8-10 weeks, and a final call at 4 months postpartum.	Not mentioned
Althabe, 2016 (36)	Not mentioned	The intervention in the intervention group consisted of training ANC providers on implementing the 5As intervention during ANC, with two main implementation models, additional components like printed materials and reminders, and monthly monitoring visits.	The control group in the study received a brief seminar to increase awareness of the importance of smoking cessation during pregnancy.	Nurse<br>Midwife<br>GP	The intervention lasted for 14-18 months and included a 2-day workshop for ANC providers, followed by replication of training to other providers, distribution of printed materials, and monthly monitoring of implementation.	Not mentioned
McBride, 2005 (37)	"Partner-assisted", "Women-only", "Usual care"	The intervention group in the study received six telephone counselling sessions from a health advisor using motivational interviewing techniques to encourage smoking cessation and abstinence, both during pregnancy and postpartum. The counselling focused on communication, developing alternatives to negative	The control group in the study received six telephone counselling sessions from a health advisor using motivational interviewing techniques to encourage smoking cessation and abstinence, a late-pregnancy relapse-prevention kit (gift item and booklet), and usual care including smoking cessation advice and a self-help smoking	Advisor	The intervention involved six telephone counselling sessions in total, with three sessions during pregnancy and three sessions postpartum for both the partner-assisted and women-only groups.	Not mentioned

		behaviour, planning for high-risk situations, and potentially encouraging the partner to quit smoking. Additionally, the women received a late-pregnancy relapse-prevention kit and usual care.	cessation guide targeted to pregnant women.			
Cope, 2003 (38)	Smokescreen 1	The intervention group in the study involved both smokers and non-smokers who were given a brief explanation of the smoking test and research aims at their initial visit. A specific quit date was chosen within 14 days, participants received a printed leaflet with advice on smoking reduction, and the protocol was repeated at subsequent visits up to the 36-week mark.	The control group in the study did not receive the point-of-care smoking test but instead received counselling about smoking in pregnancy from their hospital midwife and obstetrician as part of routine care.	Nurse Midwife Research Team Advisor	The intervention sessions involved an initial visit interview and smoking test, visual and numerical feedback, follow-up and retesting at subsequent visits, choosing a specific quit date within the next 14 days, and repeating the protocol at each clinic visit up to and including the 36-week visit.	Not mentioned
Pollak, 2020 (39)	SMS text-delivered scheduled gradual reduction (SGR) program plus support texts	The intervention group involved pregnant women who were asked to gradually reduce their smoking from baseline to 0 cigarettes by the 4th or 5th week of enrolment. They received scheduled texts for smoking times and supportive messages, were required to text back after alerts, and set a quit date within 2-3 weeks.	The control group in the study received theory-based support messages throughout pregnancy to promote cessation and prevent relapse, without the scheduled gradual reduction intervention provided to the intervention group.	Research Team	The intervention lasted from the first day of the 4th or 5th week of enrolment until the end of the study at 35 weeks gestation. Women received 1-2 text messages per day during weeks 1-6 and three texts per week from week 8 until 35 weeks gestation. The program aimed to gradually reduce smoking from baseline to 0 cigarettes by the first day of the 4th or 5th week of enrolment, with a reduction algorithm of 1/3 fewer cigarettes per day each week.	The role of technology was to deliver a smoking cessation intervention via SMS text messages. This included sending scheduled alert texts for smoking reduction and supportive messages as part of a randomized controlled trial to help pregnant women quit smoking. Technology was

						also used to assess adherence and gather feedback on the intervention's helpfulness.
Yang, 2016 (40)	Not mentioned	The intervention group received a multi-component intervention including large group educational sessions, clinician advice, monthly follow-up calls, and educational materials/resources. The educational activities covered motivational speeches, lectures on smoking dangers and SHS, videos, role-playing exercises, and games on SHS knowledge. The intervention lasted for approximately 6 months after enrolment and ended before the participant gave birth.	The control group in the study received usual clinical care without any specific intervention related to reducing second-hand smoke (SHS) exposure.	Research Team	The duration of the intervention consisted of three large group educational sessions over 3 months, along with standardized clinician advice at prenatal check-ups, brief monthly follow-up calls, and the provision of educational materials and resources.	Not mentioned
Marin-Gomez, 2019 (41)	Tobbstop	The intervention group used the Tobbstop serious game app on their mobile devices for 3 months to support smoking cessation by guiding players through a detoxification process represented as cleaning and purifying an initially polluted island. Continuous smoking abstinence until delivery was the primary outcome measured using carbon monoxide concentration, with progress monitored based on specific variables.	The control group in the study received standard care for smoking cessation without the use of the Tobbstop app, while undergoing the same assessments as the intervention group during follow-up visits.	Midwife	The duration of the intervention was 3 months, starting 1 week before the chosen cessation day and continuing until 90 days after that day. The frequency of engagement with the serious game was continuous throughout this period.	The role of technology was used to deliver a digital intervention through a mobile app called Tobbstop, which is a serious game designed to help pregnant smokers quit smoking. The app is used by the intervention group on their mobile phones or tablets for three months

						and incorporates behaviour change techniques and gamification. The effectiveness of the app is measured by continuous smoking abstinence at delivery, validated by carbon monoxide concentration tests.
Xia, 2020 (42)	Not mentioned	The intervention group received 4 videos on the risks of smoking for maternal and child health via WeChat, with content developed using the theory of planned behaviour. Participants found the videos easy to comprehend and comprehensive, leading to increased awareness of smoking hazards. The video group had a significantly higher validated abstinence rate compared to the text group at 6 months.	Participants in the control group received a leaflet with generic information on smoking cessation at baseline and did not receive any further intervention during the study period.	Research Team	The duration of the intervention was 7 weeks, with participants receiving 4 videos focusing on different risks of smoking for maternal and child health, each lasting approximately 1 minute.	Technology played a crucial role in delivering the intervention in this study. It was used to send videos and text messages to participants through instant messaging, specifically WeChat, allowing for convenient access to the intervention content.
Loukopoulou, 2018 (43)	Intensive smoking cessation intervention	The intervention in the experimental group included a 30-minute face-to-face cognitive-behavioural counselling session based on the '5As' model, delivered by a trained nurse, along with a	The control group intervention involved minimal contact with participants, including a 5-minute face-to-face communication session, brief advice, and a leaflet on smoking cessation during pregnancy.	Nurse	The high intensity smoking cessation intervention involved a single 30-minute behavioural counselling session, while the minimal contact intervention for the control group included face-	Not mentioned

		tailored self-help manual covering key points on smoking effects during pregnancy, benefits of cessation, preventing relapse, and practical solutions for quitting.			to-face communication for 5 minutes.	
Keten, 2013 (44)	Not mentioned	The intervention group (Group I) received brief education, telephone counselling, and a booklet explaining the effects of smoking during pregnancy, benefits of smoking cessation, and coping strategies with withdrawal symptoms through a letter from the foetus to the mother. This combination was found to be more effective in smoking cessation.	The control group (Group III) received brief education and counselling only, without additional support like telephone counselling or booklet.	Nurse Midwife	The duration and frequency of the intervention sessions varied across the three groups. Group I received the most comprehensive intervention with additional materials and more sessions compared to Group II and Group III.	Not mentioned
Tsoh, 2010 (45)	Video Doctor plus provider cueing	The intervention group received the Video Doctor plus provider cueing intervention, which included interactive tailored messages for participants, a cueing sheet for providers summarizing the patient's risk profile and suggested counselling statements, and an educational worksheet for participants with self-reflection questions, harm reduction tips, and local resources.	Participants in the control group did not receive the Video Doctor intervention, cueing sheet, or educational worksheet. They proceeded directly to their prenatal appointment and received usual care determined by the clinician.	GP	The duration of each intervention session was 15 minutes, and the frequency of the intervention was at baseline and 1 month before routine prenatal visits.	Use of the Video Doctor, a multimedia interactive intervention delivered on a laptop computer. This technology provided tailored messages to pregnant smokers and generated cueing sheets for providers and educational worksheets for participants.
Forinash, 2018 (46)	Text messaging	The intervention group received text messaging with motivational messages focused on smoking cessation and	The control group received a 40-minute smoking cessation visit with a pharmacist, pharmacotherapy if appropriate	Research Team Pharmacist	The duration of the intervention was until completion of pharmacotherapy or 8 weeks for	Technology was used to deliver motivational text messages to

		pregnancy in addition to standard of care smoking cessation services.	(NRT or bupropion), follow-up calls, and biweekly visits for therapy refills.		those not using pharmacotherapy,	pregnant women as part of a smoking cessation intervention. These messages were sent through Google Voice and were intended to support and encourage the women in their efforts to quit smoking.
Soltani, 2019 (47)	Family counselling using the BASNEF model	The intervention in the intervention group consisted of four weekly family counselling sessions using the BASNEF model, including lectures, group discussions, brainstorming, and distribution of educational brochures about the harms of smoking on pregnant women and unborn children. Each session lasted 45-60 minutes.	The control group received routine prenatal care without any specific family counselling sessions or interventions based on the BASNEF model.	Research Team	The intervention consisted of four weekly family counselling sessions, each lasting 45-60 minutes, including 45 minutes of counselling and 15 minutes of questions and answers.	Not mentioned
Albrecht, 1998 (48)	Teen freshstart (TFS) and Teen Freshstart Plus Buddy (TFSB)	The intervention group used a modified Teen FreshStart (TFS) program with one-to-one peer support through a non-smoking peer selected by the subject. The peer attended all eight sessions to provide support and reinforcement for smoking cessation.	The control group received a 30-minute individual educational session with a project nurse, including information about the risks of smoking to both the teen and the foetus, along with written materials like brochures on smoking and pregnancy.	Nurse	The intervention consisted of eight sessions in total, with the Teen FreshStart program having eight modules designed for smoking cessation. The Teen FreshStart with a buddy program included peer support through a non-smoking peer attending all eight sessions with the subjects. The Usual Care intervention involved a single 30-minute individual educational session with the project nurse.	Not mentioned

Stanton, 2004 (49)	Not mentioned	The intervention group in the study received a multifaceted intervention program including an 18-minute video, nicotine patches with information, and support materials like newsletters with tips on quitting, delivered in multiple stages.	The control group received a brochure providing contact details for smoking cessation options.	Research Team<br>GP	The duration of the intervention program was approximately 6 months, with intervention components being sent at different intervals throughout the program.	Not mentioned
Hjalmarson, 1991 (50)	Self-help manual	The intervention group in the study received a self-help manual designed for pregnant women, with a preparatory phase lasting one week and a cessation phase providing daily information for the first 5 days after quitting, based on behaviour therapy principles. The manual included tasks like nicotine fading, recognizing triggers, relaxation techniques, coping strategies, specific advice, and relapse prevention counselling.	The control group received an information sheet with basic facts about smoking and pregnancy, along with a recommendation to stop smoking.	OB-GYN	The intervention consisted of a self-help manual divided into a one-week preparatory phase and a five-day cessation phase with daily assignments and information.	Not mentioned
Windsor, 1985 (51)	A Pregnant Woman's Self-Help Guide to Quit Smoking	The intervention group in the study received a 10-minute skills counselling session and were taught to use a self-help manual called "A Pregnant Woman's Self-Help Guide to Quit Smoking," which included a seven-day quit plan and 10 specific skills related to smoking cessation.	The control group received routine smoking cessation advice during a prenatal education session but did not receive any additional structured intervention or self-help materials.	Research Team	The intervention sessions in Group #2 and Group #3 consisted of a standardized health education skills counselling session lasting 10 minutes, provided at each clinic by the same person using a standardized protocol. The frequency of these sessions is not explicitly mentioned.	Not mentioned
Alemán, 2016 (52)	A multifaceted strategy to promote screening for tobacco exposure, based on the 'Five As' strategy for tobacco cessation	The intervention group in the study received a multifaceted strategy including brief counselling sessions based on the "Five As" strategy for tobacco cessation, training	The control group did not receive any specific training or intervention related to smoking cessation or second-hand smoke exposure. They were allowed to implement other strategies for	Midwife<br>GP	The duration of the intervention involved a two-day training workshop for facilitators, who then provided counselling at every antenatal care visit. Posters and brochures were used	Not mentioned

		workshops for healthcare providers, implementation strategies for consistent counselling, display of educational materials, and emphasis on making homes smoke-free.	smoking cessation if desired. The characteristics of the control group were similar to those of the intervention group.		to provide information on smoking risks and SHS exposure. Counselling on SHS harms was emphasized during antenatal care visits.	
Jaakkola, 2001 (53)	Population-based smoking cessation programme	The intervention group in the study consisted of pregnant women who were smokers at their first visit to maternity health clinics in the Kymi Hospital District. The intervention included a population-based smoking cessation program that involved training sessions for public health nurses and the distribution of health education material in various settings.	The control group received routine maternity care practice with documentation of family smoking habits, encouragement and assistance with smoking cessation, and provision of health education material, without a structured individual-based intervention.	Nurse	The intervention included a one-day training session for public health nurses, distribution of health education material, and routine maternity care practice for all smoking pregnant women in the target population. No structured individual-based intervention was provided, and all counselling was integrated into scheduled visits.	Not mentioned
Price, 1991 (54)	Not mentioned	The American Lung Association's intervention group received a self-help booklet emphasizing behaviour modification skills, relaxation techniques, and support from significant others, while the physician's advice group received usual information from their physicians without a standardized protocol.	The control group received usual information from physicians about the importance of not smoking while pregnant, without a standardized protocol for communication. Smoking was identified as a risk factor and was addressed during prenatal visits.	Research Team<br>GP<br>Advisor	The duration and frequency of the intervention sessions varied: participants in the American Lung Association group received a booklet with a brief overview, participants in the physician's advice group received usual information from their physicians, and participants in the educational videotape group watched two videos (6.5 minutes initially and 4 minutes at the next visit) with a health educator present to answer questions.	The role of technology in this study was the use of educational videotapes as part of the intervention to inform pregnant women about the risks of smoking and strategies for quitting.
Higgins, 2022 (55)	Best practices combined with financial incentives (BP + FI)	Participants in the BP + FI group received voucher-based financial incentives contingent on meeting specific criteria	The control group in the study received usual care for smoking cessation, including referral to a tobacco quit line, brief-cessation	Research Team	The intervention sessions or program involved participants selecting a quit date within two weeks of enrolment, attending	Not mentioned

		related to breath CO and urine-cotinine levels, with different values for lighter and heavier smokers, to encourage smoking cessation.	counselling, and referral to the Vermont quit line for perinatal-specific individualized counselling. The never-smokers condition completed the same assessments without counselling or quit line referrals.		clinic or meeting with staff for monitoring during the initial 5 days of cessation, and then having monitoring sessions at decreasing frequencies until 12-weeks postpartum. Vouchers were earned based on meeting specific criteria related to breath CO and urine-cotinine levels, with mean average earnings of $510.02.	
Herbert, 2011 (56)	Not mentioned	The intervention group received 2-hour group sessions once a week for three weeks, followed by weekly telephone calls for three additional weeks. Participants were asked to implement a total ban on smoking in the household and engaged in discussions, problem-solving, action planning, and reflection on the effectiveness of the plan. The sessions covered topics such as the effects of environmental tobacco smoke, benefits of smoke-free homes, and strategies for creating smoke-free environments.	The control group received a brochure on environmental tobacco smoke (ETS) but did not participate in the group sessions or follow-up telephone calls that were part of the intervention.	Research Team	The intervention consisted of three weekly group sessions held once a week for three consecutive weeks, followed by three weekly follow-up telephone calls over six consecutive weeks. Each group session lasted for 2 hours and was interactive, guided by an experienced facilitator. Participants were encouraged to implement a total ban on smoking in the household.	Not mentioned
Patten, 2020 (57)	Healthy pregnancies project	The intervention group in the study consisted of a community-level intervention for Alaska Native women in rural western Alaska, which included a social marketing campaign focusing on healthy pregnancies and individual peer counselling sessions delivered by local AN "Native Sisters" based on the 5	The control group received usual care, including provision of written materials on tobacco use risks during pregnancy, minimum cessation counselling, referral to clinical tobacco cessation services, and access to state quit line cessation resources.	Advisor	The intervention consisted of up to 3 sessions during pregnancy and up to 3 sessions postpartum, lasting approximately 10-60 minutes each. The mean number of calls completed was 2.4, with an average duration of 22 to 25 minutes. Completion rates were 60% for at least one call during	The role of technology in delivering the intervention included the use of DVDs for digital stories, posting information on village-specific

		As approach for being tobacco-free. The intervention aimed to lower the tobacco use rate at 6 months postpartum, with higher campaign exposure being associated with tobacco abstinence at 6 months postpartum among intervention participants.			pregnancy and 50% during postpartum.	Facebook pages, and suggestions for future use of smartphones and social media platforms like Facebook for broader dissemination and engagement.
Everett-Murphy, 2010 (58)	ACOG 5As Guideline	The intervention group in the study received a smoking cessation intervention that included brief counselling by midwives trained in the ACOG 5As Guideline, support from peer counsellors, and tailored educational materials such as a self-help Quit Guide and leaflets for women and their partners or family members.	The control group, or "natural history cohort," received usual care which consisted of midwives asking about smoking status and advising pregnant women to quit or reduce smoking in a prescriptive manner without offering practical assistance or in-depth counselling. The only educational materials available were general pregnancy pamphlets with minimal information on smoking.	Midwife	The duration of the intervention spanned from February to November 2007. The frequency of the intervention sessions is not explicitly stated, but it involved routine clinic visits where brief counselling based on the ACOG 5As Guideline was delivered by midwives and peer counsellors. Midwives received a total of four hours of training for the intervention. Follow-up discussions about smoking were also part of the intervention and were primarily conducted by the Stop Smoking counsellor during subsequent clinic visits.	Not mentioned

Table 6.2. Detailed outcomes and effect sizes of each intervention

Author, year	Outcomes defined	Outcome measurement	Effect size calculation measure	Adjusted variables	Exclusion criteria
Eades, 2012 (1)	- Smoking status between 36 weeks' gestation and delivery.	Smoking status self-report<br>Urinary cotinine level	Risk Ratio (RR) or Relative risk	- Recruitment centre	- Pregnancy complicated by a mental illness<br>- Receiving treatment for chemical dependencies other than tobacco or alcohol use
Panjari, M., 1999 (2)	- Smoking status during pregnancy<br>- Mean cotinine concentrations in early and late pregnancy<br>- Average number of cigarettes smoked per day<br>- Postpartum smoking status	Smoking status self-report<br>Urinary cotinine level	Not mentioned	Not mentioned	- Gestation greater than 20 weeks at first visit<br>- Twin pregnancy<br>- Inability to speak and read English<br>- Drug dependency requiring referral to the Chemical Dependency Unit
Bailey, 2015 (3)	- Smoking status at entry to prenatal care<br>- Smoking status by third trimester<br>- Amount of smoking at entry to prenatal care and at delivery<br>- Number of quit attempts during pregnancy<br>- Percentage of participants who quit smoking by the end of the second trimester	Smoking status self-report<br>Urinary cotinine level<br>Exhaled CO reading	Not mentioned	Not mentioned	- Women whose pregnancy ended prior to 20 weeks.<br>- Women who delivered less than 4 weeks after entering prenatal care.
Moore, 2002 (4)	- Validated smoking cessation at the end of the second trimester of pregnancy<br>- Smoking status<br>- Cigarette consumption among daily smokers	Urinary cotinine level<br>Smoking status self-report	Not mentioned	- Cigarette consumption before pregnancy<br>- Cigarette consumption at recruitment	- Women under 16 years of age<br>- Women over 17 weeks pregnant<br>- Women unable to speak sufficient English<br>- Women who did not provide written consent<br>- Women who experienced miscarriage, termination, delivery before 27 weeks, stillbirth, or neonatal death after recruitment

Polańska, 2004 (5)	- Effectiveness of anti-smoking counselling<br>- Smoking cessation rates<br>- Reduction in smoking<br>- Maintenance of smoking abstinence	Smoking status self-report	Odds Ratio (OR)	- Number of children<br>- Level of smoking addiction<br>- Marital status<br>- Age<br>- Level of education<br>- Number of cigarettes per day smoked before the first prenatal visit<br>- Smoking husband	- Women who had a miscarriage<br>- Women who were lost to follow-up (although they were considered as smokers in the analysis)
Tappin, 2005 (6)	- Self-reported smoking cessation verified by plasma or salivary cotinine concentration<br>- Quitting defined as self-report plus cotinine concentrations of < 13.7 ng/ml serum or < 14.2 ng/ml saliva<br>- Cutting down as self-report of smoking half that at booking plus cotinine concentrations half the previous measurement<br>- Same as self-report unless cotinine concentration was twice the previous level<br>- More as cotinine concentration twice that at booking or self-report of twice the amount smoked<br>- Use of nicotine replacement therapy during pregnancy	Smoking status self-report<br>Plasma cotinine concentration<br>Salivary cotinine concentration	Relative risk (RR)	- Maternal age<br>- Parity<br>- Height<br>- Body mass index (BMI)<br>- Ethnic group<br>- Smoking status at booking	Not mentioned
Mcdonnell, 2023 (7)	- Continuous abstinence from smoking before 28+0 weeks' gestation sustained throughout pregnancy<br>- Smoking cessation rates at delivery<br>- Number of cigarettes smoked daily at the time of delivery<br>- Postpartum relapse rates at 4 to 6 months following delivery	Smoking status self-report<br>Measurement of exhaled breath CO<br>Urinary cotinine level	Odds Ratio (OR)	- Age<br>- Parity<br>- History of foetal growth restriction (FGR)	- Presence of significant foetal anomaly<br>- Maternal intellectual disability or other inability to provide informed consent<br>- Lack of spoken English ability<br>- Maternal need for specialist antenatal care owing to significant medical, psychiatric, or other disorder

Hegaard, 2003 (8)	- Self-reported smoking cessation in the 37th week of pregnancy<br>- Cotinine-validated smoking cessation in the 37th week of pregnancy	Smoking status self-report<br>Salivary cotinine concentration	Odds Ratio (OR)	- Caffeine consumption (mg/day)<br>- Previous attempts at smoking cessation<br>- Exposure to passive smoking outside the home (no/yes)<br>- Years in school (≥ 12/< 12)	- Inability to speak Danish<br>- Age below 18 years<br>- Gestation of more than 22 weeks<br>- Verified psychiatric diseases<br>- Alcohol or drug abuse
Mejdoubi, 2014 (9)	- Prevalence of cigarette smoking (percentage of smokers)<br>- Average number of cigarettes smoked per day<br>- Quantity of cigarettes smoked near the baby	Smoking status self-report	Odds Ratio (OR) and Mean differences (β)	Not mentioned	Not mentioned
Tappin, 2015 (10)	- Quit in late pregnancy<br>- Quit at 4 weeks<br>- Quit at 6 months after birth	Smoking status self-report<br>Carbon monoxide breath test<br>Salivary cotinine concentration<br>Urinary cotinine level<br>Plasma cotinine concentration	Relative risk	- Baseline smoking dependence (Fagerstrom questionnaire)<br>- Deprivation status	- Smokers with an exhaled carbon monoxide level below 7 ppm<br>- Individuals under 16 years of age<br>- Those more than 24 weeks pregnant<br>- Non-residents of NHS[1] Greater Glasgow and Clyde<br>- Individuals unable to understand and speak English for telephone consent<br>- Participants that stop smoking services were unable to contact
Tappin, 2022 (11)	- Self-reported smoking cessation in late pregnancy corroborated by saliva cotinine and anabasine levels.<br>- Engagement with stop smoking services.<br>- Biochemically validated abstinence from smoking at four weeks after the stop smoking date.<br>- Point abstinence from smoking six months after the expected date of delivery.<br>- Continuous abstinence from smoking from late pregnancy to six months postpartum.	Salivary cotinine concentration<br>Salivary anabasine concentration	Odds Ratio (OR)	- Age<br>- Smoking years<br>- Index of multiple deprivation<br>- Fagerström score<br>- Before or after COVID-19 lockdown<br>- Recruitment site<br>- Height and weight of the mother at booking (for birth weight analysis)<br>- Income status (for birth weight analysis)	- Non-smokers (did not smoke at least one cigarette in the past week)<br>- Women under 16 years of age<br>- Women more than 24 weeks gestation<br>- Non-English speakers (unable to provide verbal telephone consent)

Mcleod, 2004 (12)	- Rates of smoking cessation or reduction<br>- Smoking status changes during pregnancy and postnatally<br>- Plans to stop or reduce smoking<br>- Associations between socio-demographic variables and smoking cessation<br>- Relationship between smoking cessation and breastfeeding outcomes	Smoking status self-report<br>Plasma cotinine concentration	Odds Ratio (OR)	- Deprivation (assessed by CSC and NZDep96)<br>- Age<br>- Weeks gestation at registration with the midwife<br>- Maori ethnicity<br>- Socio-demographic variables (potentially including income and educational achievement)	- Women who moved away from the study locality<br>- Women who did not want to change their smoking behaviour<br>- Women who experienced a miscarriage<br>- Women who left the study locality
Hajek, 2001 (13)	- Continuous abstinence for at least 3 months prior to delivery<br>- Point prevalence abstinence immediately post-delivery<br>- Continuous abstinence from 3 months pre-delivery to 6 months post-delivery	Expired-air carbon monoxide (CO) reading	Odds Ratio (OR)	- Desire to stop smoking<br>- Time to first cigarette<br>- Smoking status at booking<br>- Cigarettes per day when smoking<br>- Continuous abstinence for 12 weeks up to birth<br>- Married/living with partner<br>- No educational qualifications	- Non-smokers or those who quit smoking more than 3 months prior to the study<br>- Women who did not provide informed consent<br>- Participants who moved away and were untraceable<br>- Participants deemed unsuitable for follow-up, for example, due to miscarriage
Dornelas, 2006 (14)	- Smoking status at baseline, end of pregnancy, and 6 months post-partum<br>- Point prevalence abstinence at end of pregnancy and 6 months post-partum	Smoking status self-report<br>Carbon monoxide reading	Not mentioned	- Age of participants<br>- Week of gestation<br>- Number of existing children<br>- Number of previous pregnancies	- Recent history (previous 6 months) of abuse or dependence on alcohol or other non-nicotine substance<br>- Major psychiatric illness<br>- Lack of telephone
Olson, 2019 (15)	- Quitting smoking without relapse during pregnancy<br>- Smoking cessation in pregnancy that continued to the postpartum visit at 6-8 weeks	Urinary cotinine level	Odds Ratio (OR)	- Medicaid/uninsured vs. Private insurance<br>- Education (years)<br>- Prior births >20 weeks<br>- Confidence can quit<br>- Intervention vs. Control group	- Women who had already quit smoking upon learning of their pregnancy before the first prenatal visit.<br>- Women who were seen only once after their enrolment visit.<br>- Subjects for whom cotinine logs were misfiled and could not be accessed.<br>- Subjects affected by an interruption in the intervention protocol at one clinic during staff changes, where no financial incentives were provided.<br>- Women who transferred care to another facility.

					- Women who had an early miscarriage or termination of pregnancy.
Gielen, 1997 (16)	- Smoking cessation (measured by self-report and biochemical confirmation)<br>- Significant reduction in cigarette consumption<br>- Smoking patterns (including pre-pregnancy quit attempts and relapse/slip episodes)<br>- Support from family and friends in smoking cessation efforts	Smoking status self-report<br>Salivary cotinine concentration	Not mentioned	Not mentioned	- Smokers who were 28 weeks or more pregnant<br>- Smokers who were neither African-American nor White<br>- Smokers who were changing to another prenatal clinic<br>- Smokers who could not complete the baseline interview at the first prenatal visit
Rigotti, 2006 (17)	- Cotinine-verified 7-day point-prevalence non-smoking at end of pregnancy<br>- Cotinine-verified 7-day non-smoking at 3 months postpartum<br>- Sustained abstinence (cotinine-verified 7-day abstinence at both outcome points)<br>- Significant reduction in smoking (≥ 50% decrease in cigarettes per day from baseline) assessed by self-report<br>- Number of quit attempts (defined as ≥ 24 hours of self-reported abstinence) between enrolment and end of pregnancy	Salivary cotinine concentration<br>Smoking status self-report	Odds Ratio (OR)	- Parity<br>- Weeks gestation at enrolment<br>- Prior quit attempt in this pregnancy<br>- Partner social support<br>- Referral source<br>- Age<br>- Cigarettes per day	- Referral after 26 weeks of gestation<br>- Miscarriage<br>- Patient denial of smoking in the past week<br>- Under 18 years of age<br>- Not willing to consider altering their smoking during the pregnancy<br>- Not reachable by telephone<br>- Not English-speaking<br>- Not expected to live in New England for the next year
Coleman, 2021 (18)	- Validated prolonged abstinence between 4 weeks after randomisation and late pregnancy<br>- Self-reported prolonged abstinence<br>- Seven-day abstinence at both 4 weeks (self-report) and late	Smoking status self-report<br>Salivary cotinine concentration<br>Exhaled CO reading	Odds Ratio (OR)	- Allocation<br>- Weeks' gestation at baseline<br>- Recruitment site<br>- Partner's smoking status<br>- Strength of nicotine dependence<br>- Educational attainment<br>- Maternal education	- Already using text message support for smoking cessation<br>- Non-smokers or those who smoked less frequently than one daily cigarette<br>- Under 16 years of age<br>- Beyond 25 weeks gestation<br>- Unable to receive or understand English text messages

	pregnancy (validated)<br>- Self-reported 7-day abstinence at both 4 weeks and late pregnancy<br>- Validated 7-day abstinence at late pregnancy<br>- Self-reported 7-day abstinence at late pregnancy<br>- Self-reported 7-day abstinence at 4 weeks<br>- Use of nicotine replacement therapy<br>- Use of e-cigarettes				
Ershoff, 1999 (19)	- Biochemically confirmed abstinence from smoking<br>- Quit rates by intervention group<br>- Smoking-related outcomes among self-reported smokers (including attempts to quit and periods of abstinence)	Urinary cotinine level	Not mentioned	- Baseline smoking rate<br>- Confidence in ability to quit<br>- Exposure to passive smoke<br>- Education	- Women under the age of 18<br>- Women beginning prenatal care beyond the 26th week of pregnancy<br>- Non-English speaking women<br>- Women with a pre-pregnancy smoking rate of less than 7 cigarettes per week<br>- Women who had not smoked in the seven days prior to the interview<br>- Women who had experienced a miscarriage or abortion
Walsh, 1997 (20)	- Smoking status via self-reports<br>- Smoking status via urine cotinine tests<br>- Point prevalence of cessation of smoking based on self-reports and chemical validation<br>- Indicators of smoking cessation on consecutive visits based on self-reports and chemical validation	Smoking status self-report<br>Urinary cotinine level	Not mentioned	Not mentioned	- More than 26 weeks' gestation<br>- Judged by the midwife to be too ill<br>- Judged by the midwife to be psychologically disturbed
Stotts, 2002 (21)	- Smoking abstinence at 34 weeks of pregnancy as indicated by cotinine levels<br>- Self-reported smoking status at 6 weeks postpartum<br>- Self-reported smoking status at	Urinary cotinine level<br>Smoking status self-report	Not mentioned	- Number of cigarettes smoked per week at baseline<br>- Partner smoking status	- Non-fluency in English<br>- Age under 18 years<br>- Smoking frequency less than 5 cigarettes per week prior to pregnancy<br>- Gestational age at first prenatal visit over 20 weeks

	3 months postpartum<br>- Self-reported smoking status at 6 months postpartum<br>- Current stage of change for pregnancy smoking cessation				- No longer in care at the clinic by the 28th week of gestation<br>- Had a foetal demise or abortion<br>- New due date indicating gestational age over 34 weeks<br>- Could not be reached for the follow-up interview at approximately the 28th week of gestation
Secker-Walker, 1994 (22)	- Smoking status measured by self-report<br>- Smoking status measured by urinary cotinine/creatinine ratios<br>- Quitting rates during pregnancy<br>- Long-term postpartum quitting rates<br>- Influence of other smokers in the household on quitting rates	Smoking status self-report<br>Urinary cotinine level	Odds Ratio (OR)	- Cigarettes per day at the first prenatal visit<br>- The presence of other smokers in the household<br>- The level of intention to quit smoking during pregnancy	- Women more than 25 weeks pregnant at their first prenatal visit<br>- Women who quit smoking before their first prenatal visit<br>- Women who experienced miscarriages, foetal deaths, or infant deaths during the study
Britton, 2006 (23)	- Self-reported smoking status during pregnancy and postpartum<br>- Validated smoking cessation rates using urinary cotinine assays during pregnancy and postpartum<br>- Discordance between self-reported smoking status and urinary cotinine assays<br>- Influence of the intervention on stability in recent quitters<br>- Influence of the intervention on women reporting that they smoked at least 1 cigarette/day at the first prenatal visit<br>- Cotinine-validated quit rates among women who smoked at the beginning of their pregnancies	Smoking status self-report<br>Urinary cotinine level	Not mentioned	- Group<br>- Place of residence<br>- Age<br>- Grade<br>- Type of insurance<br>- Marital status<br>- Gravidity<br>- Parity<br>- Number of smokers in the home<br>- Practice setting<br>- Number of cigarettes smoked per day at the first prenatal visit	- Women who were not confirmed to be pregnant<br>- Women who did not self-report as smokers at the onset of pregnancy<br>- Women who were more than 16 weeks into their gestation at the time of the study<br>- Women who were not receiving prenatal care at the participating private offices

	- Sustained validated quit rates at postpartum				
O'Connor, 1992 (24)	- Smoking cessation confirmed by urinary cotinine levels<br>- Self-reported cigarette smoking<br>- Number of cigarettes smoked daily	Smoking status self-report<br>Urinary cotinine level	Relative risk (RR)	Not mentioned	- Less than 31 weeks' gestation<br>- Missed abortions<br>- Consultations for therapeutic abortion<br>- Congenital anomaly<br>- Participation in the study during a previous pregnancy<br>- Severe retardation, psychiatric illness, or only 1-day consultation
Pbert, 2004 (25)	- Smoking status at the end of pregnancy<br>- Smoking status at 1 month postpartum<br>- Smoking status at 3 months postpartum<br>- Smoking status at 6 months postpartum<br>- Number of cigarettes smoked prior to pregnancy<br>- Time to first cigarette after waking, prior to pregnancy<br>- Biochemical validation of smoking abstinence via salivary cotinine analysis	Smoking status self-report<br>Salivary cotinine concentration	Odds Ratio (OR)	Not mentioned	- Not speaking English or Spanish<br>- Being less than 2 months before their due date<br>- Not being a current smoker or a spontaneous quitter (quit smoking after learning of pregnancy)<br>- Not planning to remain in the area for at least 6 months following delivery
Luk, 2021 (26)	- Biochemically validated tobacco abstinence at 6 months after intervention initiation<br>- Self-reported 24-week continuous abstinence at 6 months after intervention initiation<br>- Self-reported 7-day point prevalence abstinence at 3 and 6 months after intervention initiation<br>- Use of any nicotine replacement therapy (NRT) at 3 and 6 months after intervention	Exhaled carbon monoxide level using a Smokerlyzer device for biochemical validation.<br>Self-reported measures for continuous abstinence, point prevalence abstinence, use of NRT, and use of smoking cessation services	Odds Ratio (OR)	Not mentioned	- Smokers with a history of angina, arrhythmia, or acute myocardial infarction<br>- Individuals with psychiatric diseases<br>- Individuals taking regular psychotropic medications<br>- Those who used a smoking cessation aid or program in the past 3 months

	initiation<br>- Use of a smoking cessation service at 3 and 6 months after intervention initiation<br>- Self-reported smoking reduction at the 3-and 6-month follow-up				
de Vries, 2006 (27)	- 7-day abstinence<br>- Continuous abstinence<br>- Partner smoking	Smoking status self-report<br>Urinary cotinine level	Odds Ratio (OR)	- Age<br>- Level of education<br>- Paid job woman<br>- Number of prior pregnancies<br>- Number of weeks pregnant at intake<br>- Number of cigarettes a day before pregnancy<br>- Number of cigarettes a day at first visit with midwife<br>- Age began smoking<br>- Minutes to first cigarette<br>- Drinks alcohol<br>- Partner smoking<br>- Anticipated help in effort to quit from partner	- Women who had been pregnant more than twice<br>- Women who could not speak and understand Dutch<br>- Women who did not smoke at least one cigarette a day at the time of recruitment<br>- Women who did not consent to the study<br>- Women who were not invited by midwives due to time constraints<br>- Women who refused to participate for various reasons (did not want to quit smoking, feared confrontation with their smoking status, regarded the study as an intrusion of privacy, anticipated feelings of guilt and shame, did not want to be burdened by participating in a research study)
Cummins, 2016 (28)	- Prolonged abstinence from smoking at the third trimester (about 29 weeks' gestation)<br>- Prolonged abstinence from smoking at 2 months postpartum<br>- Prolonged abstinence from smoking at 6 months postpartum<br>- Cotinine-corrected 7-day abstinence rates at the end of pregnancy	Salivary cotinine concentration<br>Smoking status self-report	Risk Ratio (RR) or Relative risk	Not mentioned	- Active psychiatric disorders<br>- Current substance or alcohol abuse<br>- Being in recovery for ≤6 months<br>- Specifically requested counselling (precluding randomization)<br>- Planned to use pharmacotherapy<br>- Provided insufficient contact information
Lowe, 1998 (29)	- Smoking cessation rates at 20 weeks' gestation<br>- Change in smoking status (including stopped smoking and decreased smoking)	Smoking status self-report<br>Urinary cotinine level	Not mentioned	Not mentioned	- Not in their first trimester of pregnancy<br>- Had current complications<br>- Planning to give the child up for adoption at birth

Bullock, 2009 (30)	- Point prevalence abstinence in late pregnancy (measured by salivary cotinine levels)<br>- Point prevalence abstinence post-delivery (measured by salivary cotinine levels)<br>- Patterns of quitting during pregnancy (early quitters, middle quitters, late quitters, relapsers)	Salivary cotinine concentration	Odds Ratio (OR)	Not mentioned	- Ineligibility or inability to be reached for the initial home visit at the address provided to the clinic<br>- Being under 18 years of age<br>- Having a spontaneous abortion prior to the home visit
Aveyard, 2005 (31)	- Reported cessation in the woman's partner at 30 weeks gestation<br>- Reported cessation in the woman's partner at 10 days postpartum	Smoking status self-report	Odds Ratio (OR)	- Baseline cigarette consumption<br>- Fagerstrom Test for Nicotine Dependence<br>- Weeks of gestation at booking<br>- Ethnic group<br>- Parity<br>- Education<br>- Income<br>- Baseline stage of change	- Non-smokers at the time of booking for maternity care
Abroms, 2017 (32)	- 7-day biochemically confirmed point prevalence abstinence (PPA) at the 3-month follow-up<br>- 30-day biochemically confirmed PPA<br>- Self-report of abstinence (7-day and 30-day) at the 1-, 3-, and 6-month follow-up, late in pregnancy, and in the postpartum period	Smoking status self-report<br>Salivary cotinine concentration	Relative risk (RR)	- Education	- Due date less than 8 weeks in the future at the time of sending the recruitment message.<br>- Residency in California, Oklahoma, Ohio, and Louisiana where Quit4baby was already available.<br>- Not having a cell phone for personal use.<br>- Unwillingness to receive text messages on their cell phone.<br>- Being under 14 years of age.<br>- Not being currently pregnant.<br>- Not having smoked at least one puff of a cigarette in the past 2 weeks.
Mcbride, 2004 (33)	- Self-reported smoking status (assessed at each follow-up)<br>- Sustained abstinence (no smoking across all four follow-up times)<br>- Prevalent abstinence (no smoking in the prior 7 days at each of the four follow-up	Smoking status self-report<br>Salivary cotinine concentration	Odds Ratios (OR) and Hazard Ratios (HR)	- Age (years)<br>- Education (≤ high school vs > high school)<br>- Race/ethnicity (minority vs white)	- Women more than 20 weeks pregnant<br>- Women whose partners were not available for participation<br>- Women who declined to allow partner contact

	times)<br>- Postpartum relapse<br>- Partners' prevalent abstinence<br>- Biochemical confirmation of self-reported abstinence				
Malchodi, 2003 (34)	- Reduction in the number of cigarettes smoked daily<br>- Quit rates at 36 weeks' gestation<br>- Urinary cotinine levels to validate smoking status<br>- Measurement of expired carbon monoxide to evaluate smoking cessation and reduction	Smoking status self-report<br>Urinary cotinine level<br>Exhaled carbon monoxide level	Not mentioned	- Baseline number of cigarettes smoked daily	- Use of smokeless tobacco or nicotine replacement products<br>- Self-reported current substance abuse or dependence
Pollak, 2015 (35)	- 7-day point prevalence smoking abstinence<br>- Continuous abstinence<br>- 30-day point prevalence abstinence	Smoking status self-report<br>Salivary cotinine concentration	Odds Ratio (OR)	- Education (≤9th grade vs. >9th grade)<br>- Wantedness of pregnancy (yes/no)<br>- Number of cigarettes per day (≤5 vs. >5)	- Women who were smoking<br>- Women who were not between 13- and 29-weeks' gestation<br>- Women and men under 16 years of age<br>- Men who had not smoked at least 100 cigarettes in their lifetime<br>- Men who had not smoked in the past 30 days<br>- Women and men who were 16-18 years old and were not married
Althabe, 2016 (36)	- Frequency of women who recalled receiving the 5As during ANC at more than one visit<br>- Frequency of women who smoked until the end of pregnancy<br>- Quit smoking during pregnancy<br>- Rates of asking and advising on SHS at home and at work	Smoking status self-report<br>Salivary cotinine concentration	Odds Ratio (OR) and Relative Odds Ratio (ROR)	- Frequency of women recalling the 5As<br>- Frequency of women who smoked during pregnancy<br>- Relative number of providers per women attended at the clinics<br>- Relative frequency of midwives or nurses who attended ANC over the total of providers<br>- Country of location	- Did not attend ANC[2] at one of the participating clinics<br>- Mental or physical impairments preventing them from being interviewed<br>- Diagnosis of stillbirth at admission
McBride, 2005 (37)	- Women's self-reported abstinence from smoking at 12 months<br>- Abstinence at any follow up	Smoking status self-report	Not mentioned	Not mentioned	- Women under 18 years old<br>- Not current smokers or recent quitters<br>- Not living with an intimate partner<br>- Unwilling for partner to be contacted

	- Abstinence at all four follow ups<br>- Proportion abstinent at 28 weeks of pregnancy<br>- Remaining abstinent postpartum				
Cope, 2003 (38)	- Self-reported cigarette consumption changes (fall, cessation, reduction)<br>- Changes in smoking test results from 'booking' to 36 weeks' gestation<br>- Smoking cessation rates in the intervention and control groups<br>- Smoking reduction rates in the intervention and control groups<br>- Changes in smoking habits as determined by the smoking test device (including quitting, reducing, and exposure to environmental tobacco smoke or occasional cigarette use)	Urinary cotinine level	Not mentioned	- Nicotine metabolite results	- Non-smokers with a negative smoking test result<br>- Participants who failed to attend their final appointment, were lost, moved, or could not be located<br>- Participants who were referred to another care provider<br>- Participants who had a premature delivery prior to 36 weeks<br>- Participants who had a suspected miscarriage<br>- Participants who refused to take further part in the study<br>- Control group participants with false positive test results
Pollak, 2020 (39)	- Biochemically validated 7-day point prevalence abstinence at late pregnancy<br>- Reduction in cigarettes per day<br>- Percentage of participants who quit smoking by the end of pregnancy<br>- Percentage of participants who quit smoking at 3 months postpartum<br>- Percentage of participants who achieved a 50% reduction in cigarettes per day at late pregnancy<br>- Percentage of participants who smoked 5 or fewer cigarettes per day at late pregnancy	Salivary cotinine concentration	Not mentioned	- Education<br>- Partner smoking	- Women with evidence of cognitive or mental health problems who could not reliably provide informed consent were excluded from the study.

Yang, 2016 (40)	- Self-reported "no SHS exposure" before and 3 months after birth<br>- Biochemically validated SHS exposure using hair nicotine levels<br>- Self-reported past week total daily average SHS exposure<br>- Self-reported past week home daily average SHS exposure<br>- Husband's daily cigarette consumption<br>- Household smoking rule<br>- Scores of SHS knowledge<br>- Attitudes towards SHS	Smoking status self-report<br>Hair nicotine	Odds Ratio (OR)	- Age<br>- Educational background<br>- Working situation<br>- Monthly family net income<br>- Urban vs. rural status<br>- Husband's daily cigarette consumption<br>- Knowledge of SHS<br>- Attitudes towards SHS<br>- Household smoking rules	- Women who were not never smokers (i.e., past or current smokers)<br>- Women without a husband who smoked<br>- Women not living with the husband who smoked
Marin-Gomez, 2019 (41)	- Continuous tobacco abstinence until delivery confirmed by carbon monoxide levels<br>- Total days of smoking abstinence during pregnancy	Carbon monoxide concentration measured with a carboxymeter	Hazard Ratio (HR)	Not mentioned	- No interest in participation<br>- Language barriers
Xia, 2020 (42)	- Validated abstinence from smoking at the 6-month follow-up<br>- 7-day point prevalence of abstinence (PPA)<br>- Level of readiness to quit at each follow-up	Validated 7-day point prevalence of abstinence (7-day PPA) confirmed by a carbon monoxide level in expired air of less than 4 ppm<br>Self-reported 7-day PPA.	Odds Ratio (OR)	- Age<br>- Hospital<br>- Employment status<br>- Annual income level<br>- First-time expectant father<br>- Level of nicotine dependence<br>- Level of readiness to quit<br>- Smoking self-efficacy at baseline	- Inability to provide informed consent<br>- Impaired mental status<br>- Cognitive impairment<br>- Communication barriers as identified from medical records<br>- Participation in other smoking cessation programs or services
Loukopoulou, 2018 (43)	- 7-day point prevalence abstinence (self-reported and urine cotinine levels)<br>- Maternal tobacco-specific carcinogens (NNAL urine concentrations)<br>- Self-reported changes in smoking status (continuation, reduction, cessation)<br>- Changes in urinary	Smoking status self-report<br>Urinary cotinine level<br>Urinary NNAL concentration	Not mentioned	Not mentioned	- Gestational age less than 24 weeks at the time of enrolment<br>- Limited or no telephone access<br>- Not planning to live at the same address for the next year<br>- Unable to read and/or speak Greek fluently<br>- Current alcohol or substance abuse<br>- Current depression according to the Greek validated version of the GHQ[3]

	cotinine/nicotine and NNAL levels				
Keten, 2013 (44)	- Smoking behavior<br>- Smoking cessation<br>- Smoking reduction	Smoking status self-report	Not mentioned	- Age<br>- Education level<br>- Employment status<br>- Gestational week<br>- Number of pregnancies<br>- Number of births<br>- Number of living children	- Women who had smoked less than 100 cigarettes in their lifetime and were not currently smoking were excluded.<br>- Women who reported quitting smoking at the time of the interview and had not smoked in the last 7 days prior to the interview were excluded.
Tsoh, 2010 (45)	- Receipt of advice from the provider on smoking cessation<br>- Self-reported 30-day smoking abstinence<br>- Decrease in the number of days smoked<br>- Decrease in the number of cigarettes smoked per day	Smoking status self-report	Odds Ratio (OR)	Not mentioned	- Non-English speaking women<br>- Women under 18 years of age<br>- Women more than 26-weeks pregnant<br>- Women not receiving prenatal care at one of the participating clinics<br>- Women unable to complete the risk assessment on a laptop computer
Forinash, 2018 (46)	- Smoking cessation verified with exhaled carbon monoxide levels (eCO) 2 weeks from quit date<br>- Comparison between self-reported and verified cessation rates at 2 weeks<br>- Change in exhaled carbon monoxide (eCO) levels from baseline to 2 weeks	Smoking status self-report<br>Exhaled carbon monoxide levels (eCO)	Odds Ratio (OR)	Not mentioned	- Patients in the pre-contemplative or contemplative stages of change<br>- Patients who had already received SOC[4] pharmacy education during this pregnancy<br>- Patients with allergies or contraindications to NRT[5] or bupropion
Soltani, 2019 (47)	- Reduction in the number of days with reported exposure to second-hand smoke<br>- Decrease in the mean number of exposure times to second-hand smoke at home<br>- Increase in behavioural intention of spouse to reduce smoking	Smoking status self-report	Not mentioned	Not mentioned	- Presence of medical and obstetric complications<br>- Intra-uterine foetal death<br>- Divorce<br>- Change of residence<br>- Absence of more than one counselling session
Albrecht, 1998 (48)	- Smoking cessation rates (percentage of adolescents who quit smoking)	Smoking status self-report	Not mentioned	Not mentioned	- Pregnancy complications that might prevent the subject from attending all group sessions.<br>- Participation in a home-study program through

	- Reduction in the average number of cigarettes smoked per day<br>- Changes in exhaled carbon monoxide levels	Exhaled carbon monoxide level			the school (for those subjects who were still in school).
Stanton, 2004 (49)	- Self-reported cessation of smoking at 6-month follow-up<br>- Verification of smoking cessation through carbon monoxide testing	Smoking status self-report<br>Carbon monoxide level in expired air	Odds Ratio (OR)	- Baseline differences (not specified)<br>- Occupation<br>- Number of quit attempts of 2 weeks or more in the last year<br>- Time to first cigarette	- Not male<br>- Not the partner of a woman less than 25 weeks pregnant<br>- Not living with the pregnant partner (in Brisbane)<br>- Not a current cigarette smoker of at least 10 cigarettes per day within 3 days of the baseline interview
Hjalmarson, 1991 (50)	- Smoking cessation rates up to 8 weeks after delivery<br>- Acceptance rate of the self-help manual for smoking cessation<br>- Estimated quit rates for both treatment and control groups<br>- Continuous abstinence from smoking<br>- Biochemical verification of smoking status using blood thiocyanate levels	Blood thiocyanate measurement	Odds Ratio (OR)	Not mentioned	- Non-daily smokers<br>- Gestational age of 12 weeks or more<br>- Inability to understand Swedish
Windsor, 1985 (51)	- Smoking cessation confirmed by behavioural report and saliva thiocyanate (SCN) levels<br>- Smoking reduction documented by behavioural report and at least a 30 percent reduction from baseline SCN levels	Smoking status self-report<br>Saliva thiocyanate (SCN) concentration	Not mentioned	Not mentioned	- Women who entered care beyond 32 weeks of pregnancy.<br>- Women who left the system or moved from the Birmingham area.<br>- Women who chose to abort or miscarried between baseline and mid-point observation.<br>- Women who participated in group discussions that were not feasible due to scheduling issues.
Alemán, 2016 (52)	- Rates of self-reported second-hand smoke (SHS) exposure at home<br>- Rates of self-reported SHS exposure at work<br>- Rates of self-reported SHS	Smoking status self-report<br>Salivary cotinine concentration	Odds Ratio (OR), Relative Odds Ratio (ROR)	Not mentioned	- Women who continued to smoke throughout their pregnancy were not included in the secondary analysis.<br>- Individuals who did not give informed consent were excluded from the study.

	exposure indoors (including home, work, or public places)<br>- Attitudes towards SHS exposure: trying to avoid breathing smoke exhaled by people smoking near them<br>- Presence of rooms in the home where smoking was forbidden				
Jaakkola, 2001 (53)	- Quitting smoking during pregnancy<br>- Change in smoking rate in pregnancy<br>- Amount of information received from the maternity health clinics on smoking<br>- Amount of reading or studying the material on smoking<br>- Influence of the information/support received from the maternity health clinics on smoking behavior<br>- Avoidance of environmental tobacco smoke during pregnancy<br>- Number of attempts at quitting smoking during pregnancy	Smoking status self-report<br>Hair nicotine concentration	Reports percentage differences with confidence intervals as the measure of effect between the intervention and reference groups.	Not mentioned	- Non-smokers at the first visit to maternity health clinics.<br>- Women who did not give birth in the specified hospitals during the study period.
Price, 1991 (54)	- Smoking cessation<br>- Cigarette reduction	Minicarbon monoxide analyzer. Participants who scored 8 ppm or higher were considered smokers, and those who reported smoking zero cigarettes and had a reading of 7 ppm or less were considered to have quit smoking.	Not mentioned	Not mentioned	- Women who were more than 28 weeks pregnant were excluded from the study.

Higgins, 2022 (55)	- 7-day point-prevalence abstinence antepartum<br>- 7-day point-prevalence abstinence postpartum<br>- Small-for-gestational-age (SGA) deliveries	Smoking status self-report<br>Urinary cotinine level<br>Breath CO specimens	Odds Ratio (OR)	- Maternal pre-pregnancy BMI<br>- First pregnancy<br>- Foetal gender<br>- Gestational age at delivery (for analysis of birthweight)	- Incarceration<br>- Previous participation in a study on incentives for smoking cessation<br>- Residing with a current trial participant<br>- Regular use of opioid, stimulant, or antipsychotic medications
Herbert, 2011 (56)	- Average number of cigarettes smoked in the home daily<br>- Number of smokers in the home<br>- Conflict between household members regarding smoking in the home<br>- Number of quit attempts during the past 12 months by the respondent and/or other parent<br>- Maternal smoking during pregnancy<br>- Nicotine dependence as measured by the Fagerstrom Test for Nicotine Dependence (FTND)	Smoking status self-report	Not mentioned	- Season when the outcome data were collected (November–April)	- No cigarette was smoked within the home daily.<br>- The family did not have one or more children aged 5 years or younger who resided in the home at least 50% of the time.<br>- No parent was willing to participate in the intervention.
Patten, 2020 (57)	- Tobacco use rate at delivery<br>- Tobacco use rate at 2 months postpartum<br>- Tobacco use rate at 6 months postpartum<br>- Biochemically confirmed tobacco use at 6 months postpartum	Salivary cotinine concentration<br>NicAlert test strip reading	Odds Ratio (OR)	- Yup'ik language<br>- Second-hand smoke exposure<br>- CES-D score (depressive symptoms)<br>- Type of tobacco used at baseline	- Resided in a non-study village
Everett-Murphy, 2010 (58)	- Smoking cessation during pregnancy (defined as urinary cotinine level below 100 ng/ml)<br>- Smoking reduction (defined as at least half the level of urinary cotinine at study entry) - Quit attempts by self-report (defined	Smoking status self-report - Urinary cotinine level	Risk difference (RD)	- Demographic variables (unplanned pregnancies, living conditions) - Smoking behaviour variables (cotinine levels, self-reported quitting rates)	- Women who were found not to be pregnant<br>- Women beyond 24 weeks gestational age at first booking<br>- Women intending to seek private antenatal care or an abortion<br>- Women who refused to give consent

	as not having even a puff for at least 24 hours)				
Notes: [1]NHS: National Health Service; [2]ANC: Antenatal Care; [3] GHQ: Goldberg General Health Questionnaire; [4] SOC: Standard of Care; [5]NRT: Nicotine Replacement Therapy					

Table 6.3. Detailed vulnerability characteristics and approach

Author, Year, Reference	Name of intervention	Vulnerability characteristics	Approach to vulnerability characteristics
Eades, 2012 (1)	Not mentioned	- Ethnic / racial minorities<br>-Socioeconomical disadvantages<br>- Financial insecurity	The study population of the research is Aboriginal community-controlled health services, it was ensured that the intervention was culturally sensitive and specific, involving significant others to support the women, using Aboriginal or Torres Strait Islander health workers and midwives for follow-up, and providing on-site training for intervention agents by professionals familiar with the cultural context.
Panjari, M., 1999 (2)	Personalized smoking cessation intervention	- Socioeconomical disadvantages	Not mentioned
Bailey, 2015 (3)	Tennessee Intervention for Pregnant Smokers (TIPS) program	- Socioeconomical disadvantages<br>- Low educational level<br>- Young pregnancy	The study included a diverse range of prenatal care practices to ensure accessibility, not excluding any smokers from participation, and providing personalized assistance that addressed socioeconomic disadvantages and mental health issues. The intervention included practical assistance for needs such as housing, food, and transportation, as well as stress management and mental health support, which facilitated the intervention for vulnerable women.
Moore, 2002 (4)	Stop for Good	- Low educational level	Not mentioned
Polańska, 2004 (5)	Not mentioned	- Socioeconomical disadvantages<br>- Low educational level<br>- Living alone or separated from partner: some participants	Not mentioned
Tappin, 2005 (6)	Not mentioned	- Socioeconomical disadvantages<br>- Low socioeconomical level<br>- Living alone or separated from partner: some participants	The study acknowledges the link between smoking and low social class and includes deprivation categories in the baseline characteristics, indicating an awareness of socioeconomic factors.
Mcdonnell, 2023 (7)	Smoking cessation Through Optimization of clinical care in Pregnancy (STOP clinic)	- Socioeconomical disadvantages<br>- Mental disorders (depression, anxiety)<br>- Living alone or separated from partner: some participants	The study acknowledges certain vulnerability characteristics such as depression, single parenting, and the presence of a partner who smokes. It also recognizes the importance of mental health and social support for disadvantaged women and notes that smokers accessed healthcare less readily. The ethnicity of the participants is also noted, but there is no specific mention of addressing racial diversity or tailoring the intervention to different racial groups.

Hegaard, 2003 (8)	Not mentioned	- Low educational level: some participants had less than 12 years of schooling<br>- Alcohol use: some participants reported alcohol consumption	Not mentioned
Mejdoubi, 2014 (9)	Voorzorg programme	- Ethnic/racial minorities<br>- Socioeconomical disadvantages<br>- Low educational level<br>- Young pregnancy<br>- Financial insecurity<br>- Alcohol use	The study addressed vulnerability by selecting a population of young pregnant women with low socio-economic status and additional risk factors such as lack of social support, domestic violence, psychosocial symptoms, financial problems, housing difficulties, and substance abuse. The VoorZorg nurses received specialized training to support these women, focusing on smoking cessation and breastfeeding promotion. The intervention systematically inquired about smoking behaviour and offered cessation support, and after birth, it emphasized the health effects of smoking on the baby. The program also addressed barriers to breastfeeding, which can be particularly challenging for vulnerable populations.
Tappin, 2015 (10)	Up to £400 of shopping vouchers added to routine specialist pregnancy NHS 'Stop Smoking Services'	- Socioeconomical disadvantages<br>- Financial insecurity	The study included women from socioeconomically deprived areas, which suggests some consideration of vulnerability due to socioeconomic status.
Tappin, 2022 (11)	Lovetoshop financial voucher incentives	- Socioeconomical disadvantages<br>- Young pregnancy	The study addressed financial insecurity by offering free stop smoking services and financial incentives. It also considered socioeconomic status in the analysis by adjusting for the index of multiple deprivation.
Mcleod, 2004 (12)	Programme of education and support for smoking cessation or reduction, programme of education and support for breast feeding	- Ethnic/ racial minorities<br>- Socioeconomical disadvantages<br>- Low educational level<br>- Financial insecurity	The study acknowledged the higher rates of smoking among women in lower socio-economic groups and Maori women, and it collected data on ethnicity and socio-economic deprivation using the New Zealand Deprivation Index and the Community Service Card.
Hajek, 2001 (13)	Not mentioned	- Socioeconomical disadvantages<br>- Low educational level<br>- Living alone or separated from partner: some participants	Not mentioned
Dornelas, 2006 (14)	Not mentioned	- Ethnic/racial minorities<br>- Socioeconomical disadvantages<br>- Low educational level	The study took into account the vulnerability characteristics of the study population by providing an intervention that was accessible within the clinic and included additional support through telephone follow-ups. Masters-prepared mental health counsellors were employed to address mental health

		- Financial insecurity<br>- Living alone or separated from partner: some participants<br>- Depression	vulnerabilities and treat co-morbid conditions. The study recognized barriers such as transportation issues and considered the attendance rate encouraging despite these challenges.
Olson, 2019 (15)	Smoke-free moms	- Socioeconomical disadvantages<br>- Low educational level<br>- Low socioeconomical level<br>- Financial insecurity	The study focused on low-income pregnant women who were smokers at their first prenatal visit, indicating a socioeconomic disadvantage. The intervention was designed to be implemented in a clinical setting that served this population. The study considered Medicaid/uninsured status, which is often associated with financial insecurity. The baseline survey assessed mood and stress, which could be related to mental disorders or other vulnerabilities. Women we offered financial incentives for smoking cessation.
Gielen, 1997 (16)	Smoke-free moms project	- Ethnic/ racial minorities<br>- Socioeconomical disadvantages<br>- Low educational level<br>- Financial insecurity	The study took into account the vulnerability characteristics of the population by designing an intervention that was accessible to low-income, African-American women, many of whom had low educational levels. The intervention materials were written at a sixth-grade reading level, and the program was tailored to the local population with input from focus groups. A peer health counsellor from the local community was used to deliver the intervention, which could help address cultural and social barriers.
Rigotti, 2006 (17)	Proactive pregnancy-tailored telephone counselling for smoking cessation	- Ethnic/ racial minorities<br>- Socioeconomical disadvantages<br>- Low educational level<br>- Financial insecurity<br>- Depression	Not mentioned
Coleman, 2021 (18)	Miquit	- Socioeconomical disadvantages: some participants	Not mentioned
Ershoff, 1999 (19)	Not mentioned	- Ethnic/ racial minorities	The booklet provided as part of the intervention was designed to be accessible and appeal to a wide audience, including those with lower educational levels and diverse racial/ethnic backgrounds.
Walsh, 1997 (20)	Structured, cognitive-behavioural smoking cessation program	- Low educational level: some participants	Not mentioned
Stotts, 2002 (21)	One-to-One	- Ethnic/ racial minorities<br>- Low educational level	Not mentioned
Secker-Walker, 1994 (22)	Individualized smoking cessation counselling	- Socioeconomical disadvantages<br>- Low educational level<br>- Financial insecurity	The study included women from different socioeconomic backgrounds, including those with lower educational levels and those on Medicaid, which can be associated with socioeconomic disadvantages. The intervention appeared to be more effective for women receiving care in the publicly supported MIC clinic, suggesting that the intervention may have been more beneficial for this potentially more vulnerable population.

Britton, 2006 (23)	Smoke Free Baby & Me program	- Socioeconomical disadvantages<br>- Low educational level<br>- Living alone or separated from partner: some participants<br>- Financial insecurity	The study population included a significant proportion of women who were single, divorced, or separated, and over half had Medicaid insurance, indicating socioeconomic disadvantages. The study was conducted in a rural area and the sample was predominantly White, suggesting a lack of racial diversity. The study acknowledges these characteristics and suggests the need for focused attention on these groups, especially in addressing the culture that encourages tobacco use and involving women in participatory action research but does not address any issue in its implementation.
O'Connor, 1992 (24)	Windsor's self-help smoking cessation program	- Socioeconomical disadvantages<br>- Financial insecurity	The study acknowledges and addresses some aspects of vulnerability by including a diverse socioeconomic and linguistic population, providing interventions in both English and French, and individualizing the intervention.
Pbert, 2004 (25)	Quit together (qt)	- Ethnic/ racial minorities<br>- Socioeconomical disadvantages<br>- Low educational level<br>- Low socioeconomical level<br>- Financial insecurity<br>- Living alone or separated from partner: some participants	The study addressed the vulnerability of the population by providing comprehensive care through CHCs, including supplemental services that could help mitigate socioeconomic disadvantages. The intervention was tailored to the women's stage of change and delivered by existing healthcare providers, which could improve accessibility and compliance. Training for providers included information relevant to the specific needs of the population, such as the impact of smoking on nutrition and managing weight while quitting. Accommodations were made for language barriers and scheduling issues, and incentives were provided to encourage participation.
Luk, 2021 (26)	Brief advice, nicotine replacement therapy sampling, and active referral (BANSAR)	- Low educational level	Not mentioned
de Vries, 2006 (27)	MIS smoking cessation protocol	- Low educational level<br>- Alcohol use	Not mentioned
Cummins, 2016 (28)	Pregnancy-specific counselling protocol	- Ethnic/racial minorities<br>- Socioeconomical disadvantages<br>- Low educational level<br>- Low socioeconomical level<br>- Financial insecurity<br>- Living alone or separated from partner	The study was designed to be inclusive and accessible to pregnant smokers, including those with vulnerability characteristics. The counselling was offered in both English and Spanish, and the intervention included pregnancy-specific content and a more intensive counselling protocol than standard quit line counselling. The study acknowledged that many participants were economically disadvantaged and lacked social support, and proactive counselling may have provided needed support for these women.
Lowe, 1998 (29)	Healthy 2	- Socioeconomic disadvantages<br>- Low educational level	The study addressed vulnerability by developing materials specifically for women of low socioeconomic status and adapting them to a Year 5 reading level to accommodate lower educational levels. The study also took into account the preferences of the target population by using focus groups to redesign the self-help materials into a magazine-style format that was more appealing and relevant to the women.
Bullock, 2009 (30)	Baby BEEP	- Socioeconomical disadvantages<br>- Low educational level<br>- Low socioeconomical level<br>- Stress and depression	The study addressed the vulnerability of the population by targeting low-income rural pregnant women and providing individualized social support through weekly telephone calls and 24/7 beeper access to a nurse. The nurses were trained to identify and respond to psychosocial problems, which could include mental health issues or other stressors common in the lives of the participants. The use of WIC clinics

			for recruitment and the non-judgmental nature of home visits for saliva collection were also supportive measures that likely benefited the participants.
Aveyard, 2005 (31)	Pro-change programme for a healthy pregnancy	- Socioeconomical disadvantages<br>- Low educational level<br>- Young pregnancy<br>- Low socioeconomical level	Not mentioned
Abroms, 2017 (32)	Quit4baby	- Socioeconomical disadvantages<br>- Low educational level<br>- Low socioeconomical level<br>- Financial insecurity<br>- Living alone or separated from partner: some participants<br>- Substance abuse: Marijuana use<br>- Alcohol use<br>- Mental disorders (depression, anxiety)	The study targeted low-income women and those living in medically underserved areas, indicating an awareness of socioeconomic vulnerabilities. Demographic data on race/ethnicity, education, and insurance status were collected, showing that the study population included individuals with socioeconomic disadvantages. A significant portion of the study participants had mental health issues, and many were single or living with a significant other, which could indicate financial insecurity or lack of social support. The development of Quit4baby involved input from various stakeholders, which may have included considerations for the needs of vulnerable populations. Additionally, the promotion of Text4baby by governmental agencies and health plans suggests that there were efforts to facilitate the intervention for vulnerable women.
Mcbride, 2004 (33)	Usual care (UC), woman-only (WO), partner-assisted (PA)	- Ethnic/ racial minorities<br>- Low educational level<br>- Young pregnancy	Not mentioned
Malchodi, 2003 (34)	Peer-led smoking cessation program	- Ethnic/ racial minorities<br>- Socioeconomical disadvantages<br>- Low educational level<br>- Low socioeconomical level<br>- Financial insecurity<br>- Living alone or separated from partner: some participants	The study addressed vulnerability by focusing on a predominantly Hispanic population, which is a racial minority. It also served a low-income pregnant population, indicating socioeconomical disadvantages. The peer support counselling approach was chosen to create a non-hierarchical relationship and increase social support, which may be beneficial for vulnerable populations. The study included bilingual measures to accommodate participants with different language backgrounds. Peer counsellors were selected from the community and shared similar social-environmental and cultural qualities with the participants, which likely helped in addressing cultural and social vulnerabilities. The demographic characteristics of the participants indicated low educational attainment, unemployment, and single marital status, which are indicators of vulnerability.
Pollak, 2015 (35)	No lo deje para mañana deje de fumar hoy: Guía para Dejar de Fumar	- Immigrants or migration background<br>- Ethnic/ racial minorities<br>- Low educational level<br>- Young pregnancy<br>- Financial insecurity	The study addressed the vulnerability characteristics of the Latino population by recruiting Latino couples during pregnancy, considering cultural values such as familismo, tailoring the intervention to the smoking habits of the population, providing financial incentives, addressing educational barriers by discussing written materials, conducting the study in Spanish, and including young emancipated participants.
Althabe, 2016 (36)	Not mentioned	- Socioeconomical disadvantages<br>- Young pregnancy	The study indirectly addressed socioeconomic disadvantages by conducting the intervention in publicly funded health centers that were free of charge and by tailoring the intervention strategies based on formative research.

McBride, 2005 (37)	"Partner-assisted", "Women-only", "Usual care"	Not mentioned	Not mentioned
Cope, 2003 (38)	Smokescreen 1	Not mentioned	Not mentioned
Pollak, 2020 (39)	SMS text-delivered scheduled gradual reduction (SGR) program plus support texts	- Ethnic/ racial minorities<br>- Socioeconomical disadvantages<br>- Low educational level<br>- Financial insecurity	The researchers provided cell phones with unlimited texting plans to those without reliable service, developed and pilot-tested support messages with input from the target population, and offered financial incentives for survey completion. The study also provided resources to ineligible women, suggesting an effort to support all participants.
Yang, 2016 (40)	Not mentioned	- Socioeconomical disadvantages<br>- Low educational level<br>- Low socioeconomical level<br>- Financial insecurity	Not mentioned
Marin-Gomez, 2019 (41)	Tobbstop	- Low educational level	Not mentioned
Xia, 2020 (42)	Not mentioned	Not mentioned	Not mentioned
Loukopoulou, 2018 (43)	Intensive smoking cessation intervention	- Immigrants or migration background<br>- Socioeconomical disadvantages<br>- Low/ medium educational level<br>- Low socioeconomical level<br>- Financial insecurity: some unemployed	Not mentioned
Keten, 2013 (44)	Not mentioned	- Socioeconomical disadvantages<br>- Financial insecurity	Not mentioned
Tsoh, 2010 (45)	Video Doctor plus provider cueing	- Ethnic/ racial minorities<br>- Socioeconomical disadvantages<br>- Low educational level<br>- Low socioeconomical level<br>- Living alone or separated from partner: some participants<br>- Substance abuse: alcohol and illicit drug use	The study took into account the vulnerability characteristics of the study population by using a low-literacy computerized interview with audio voiceover, collecting socio-demographic information, providing compensation for participation, using non-judgmental counselling techniques, and screening for multiple behavioural risks. The intervention was designed to be accessible and sensitive to the needs of vulnerable women, including those with lower educational levels, potential financial insecurities, and those experiencing substance abuse or intimate partner violence.
Forinash, 2018 (46)	Text messaging	- Ethnic/ racial minorities<br>- Socioeconomical disadvantages<br>- Living alone or separated from partner: some participants	The study population included a majority of black patients, all of whom utilized public insurance, indicating racial and socioeconomic vulnerabilities. The clinic provided a range of services suitable for high-risk and low-risk patients, and the clinical pharmacy team offered management and consultation, which could help address the needs of patients with less healthcare access. Smoking cessation aids were

		- Mental disorders (depression, anxiety)<br>- Alcohol use	provided free of charge, which is crucial for financially insecure patients. The study also noted a high prevalence of mental illness among participants, and nearly half lived with another tobacco user, suggesting additional vulnerabilities. The authors acknowledged barriers in their patient population and specific strategies to address them were the provision of free pharmacotherapy and pharmacist-driven education.
Soltani, 2019 (47)	Family counselling using the BASNEF model	- Low educational level	Not mentioned
Albrecht, 1998 (48)	Teen freshstart (TFS) and Teen Freshstart Plus Buddy (TFSB)	- Ethnic/ racial minorities<br>- Young pregnancy	The study population included a racially diverse group, with a majority being of African-American heritage. The intervention included modifications to make it more relevant to pregnant teens and incorporated strategies to enhance engagement and participation. The significant dropout rate and transient living conditions of the participants suggest that socioeconomic challenges were present, but the paper does not detail any specific actions taken to address these vulnerabilities.
Stanton, 2004 (49)	Not mentioned	- Immigrants or migration background<br>- Ethnic/ racial minorities<br>- Socioeconomical disadvantages<br>- Low educational level<br>- Low socioeconomical level	Not mentioned
Hjalmarson, 1991 (50)	Self-help manual	Not mentioned	Not mentioned
Windsor, 1985 (51)	A Pregnant Woman's Self-Help Guide to Quit Smoking	- Socioeconomical disadvantages<br>- Low socioeconomical level	Not mentioned
Alemán, 2016 (52)	A multifaceted strategy to promote screening for tobacco exposure, based on the 'Five As' strategy for tobacco cessation	- Socioeconomical disadvantages<br>- Young pregnancy	Not mentioned
Jaakkola, 2001 (53)	Population-based smoking cessation programme	Not mentioned	Not mentioned
Price, 1991 (54)	Not mentioned	- Socioeconomical disadvantages<br>- Low educational level<br>- Young pregnancy<br>- Low socioeconomical level	The study population included vulnerable women of low socioeconomic status and with low education. The researchers chose a tailored minimal-contact intervention using media as it was considered the most practical given the resources available to this population. The majority of participants were single, not employed, and had not graduated from high school. The paper acknowledges that indigent women may

		- Financial insecurity<br>- Living alone or separated from partner	prioritize other concerns over smoking cessation and suggests that addressing the broader socioeconomic and educational needs may be necessary for more effective interventions.
Higgins, 2022 (55)	Best practices combined with financial incentives (BP + FI)	- Socioeconomical disadvantages<br>- Low educational level<br>- Substance abuse<br>- Mental disorders (depression, anxiety)	The study acknowledges the vulnerability of socioeconomically disadvantaged women to smoking during pregnancy and includes them in the study.
Herbert, 2011 (56)	Not mentioned	- Socioeconomical disadvantages<br>- Low educational level<br>- Low socioeconomical level<br>- Financial insecurity<br>- Living alone or separated from partner: some participants	The study took into account the vulnerability characteristics of the population by including a high percentage of mothers, many of whom smoked during pregnancy, and by noting the socioeconomic disadvantages of the participants, with 42% reporting a household income of less than $15,000. The recruitment strategy was designed to reach families through public health nursing offices, family resource centres, and day-care centres, which may cater to vulnerable populations. The intervention was inclusive of both smoking and non-smoking parents, and the research team was trained to be non-threatening, respectful, and accepting. Effective strategies were learned to recruit parents who might be reluctant to discuss their children's ETS exposure or who feared repercussions such as child custody issues. The study also successfully recruited participants from low-income groups and rural areas.
Patten, 2020 (57)	Healthy pregnancies project	- Ethnic/ racial minorities<br>- Socioeconomical disadvantages<br>- Low educational level<br>- Young pregnancy<br>- Low socioeconomical level	The study population included Indigenous women of low socioeconomic status, and the intervention was designed to be culturally sensitive by providing pregnancy-and culture-specific materials, counselling, and incorporating local "Native Sisters" who were bilingual and culturally competent. Training addressed historical trauma and health disparities, and traditional healthy ways of coping were encouraged. The program was advertised as a healthy pregnancies project to reduce stigma and make it more accessible.
Everett-Murphy, 2010 (58)	ACOG 5As Guideline	- Ethnic minorities<br>- Socioeconomical disadvantages:<br>- Low educational level<br>- Young pregnancy<br>- Low socioeconomical level<br>- Financial insecurity<br>- Some living alone or separated from partner	The study addressed the vulnerability characteristics of the population by tailoring intervention materials to the language, reading levels, cultural values, and life circumstances of the target group. It also took into account the high prevalence of smoking and exposure to environmental tobacco smoke in the community. The study provided tailored educational materials and self-help guides, and used local cultural formats to make the intervention more accessible and relatable.

Table 6.4. Detailed description of father involvement

Rationale for categorizing studies into following groups:

According to the evidence found in the 58 articles (1-58) chosen, the involvement of the expecting fathers was classified into *active role*, *passive role,* or *no role*, as follows:

1. ***Active role in the interventions*** when the partner was involved as receiver of the intervention along with the expectant mother.
2. ***Passive role in the interventions*** when the father himself was not considered in recruitment but received a form education or was requested to provide support.
3. ***No role in the interventions*** when the smoking status of the father was either asked to the target women or not, but the father was not involved in any activity of the intervention delivered.

Role	Author, Year, Reference	Father involvement
ACTIVE	Eades, 2012 (1)	Inviting partners or significant others to support the woman's quit attempts both verbally and by mail and asking women to bring a partner or support person with them on their second visit. Additionally, a letter was provided for other household members, including fathers or partners, requesting their support.
	Moore, 2002 (4)	Fathers or partners were provided with a booklet that accompanied the first booklet given to the pregnant women.
	Stotts, 2002 (21)	Father or partner involvement included receiving newsletters and a video related to the intervention and being considered in the individualization of counselling sessions based on their smoking status.
	Luk, 2021 (26)	Expectant fathers were the primary participants in the intervention, which aimed to help them quit smoking. They were engaged through proactive recruitment at prenatal clinics, provided with brief cessation advice, a 1-week sample of NRT, and active referral to smoking cessation services. Additionally, they received follow-up telephone boosters to support their quit attempts.
	de Vries, 2006 (27)	Fathers were provided with booklet to partners of pregnant women to emphasize the importance of quitting smoking and to offer suggestions on how to quit.
	Mcbride, 2004 (33)	Father involvement included receiving telephone counselling and a support guide to help them assist their pregnant partners in quitting smoking. Fathers who smoked were also provided with cessation aids and counselling. They received six calls from health advisors, three during pregnancy and three postpartum, which included developing a written agreement on support behaviours and stage-appropriate counselling for smoking cessation.
	Pollak, 2015 (35)	Expectant fathers were recruited and were the primary focus of the smoking abstinence outcome. The interventions compared included one that was couple-based, involving both the expectant fathers and their partners. Fathers participated in counselling sessions designed to help them quit smoking and improve communication with their partner. These sessions occurred both during pregnancy and postpartum, with at least one session postpartum including both members of the couple.
	McBride, 2005 (37)	Fathers or male partners were involved in the partner-assisted support intervention by participating in six telephone counselling sessions with their pregnant partners. These sessions included motivational interviewing techniques to encourage smoking cessation and abstinence, with a focus on

		communication about supportive and unsupportive behaviours, developing alternatives to negative behaviour, and planning for high-risk situations. Additionally, if necessary, the male partners were encouraged to quit smoking.
	Yang, 2016 (40)	Fathers were indirectly involved in the study through their relationship with the pregnant participants and their smoking behaviour, which was targeted by the intervention.
	Xia, 2020 (42)	Fathers were the primary subjects of the randomized controlled trial, which aimed to promote smoking cessation among them. They were involved by being recruited at obstetrics registration centres where they registered with their pregnant partners. The study encouraged their attendance at prenatal visits and designed an intervention to change their attitudes towards smoking, especially in light of their roles as providers and role models for their children.
	Soltani, 2019 (47)	Fathers or spouses were actively involved in the intervention by attending counselling sessions and being part of the educational process aimed at reducing second-hand smoke exposure for pregnant women. The study highlights the importance of their participation and the need for social and behavioural changes among men to take more responsibility for maternal and child health.
	Stanton, 2004 (49)	Fathers were involved in the study as participants who were identified through their pregnant partners booking into an antenatal clinic. They were eligible if they were current smokers living with their pregnant partner. Fathers participated in baseline and follow-up telephone interviews, received intervention materials including a video, nicotine patches, and support materials, and had their smoking status verified by a general practitioner. The intervention materials were tailored to fathers, focusing on their role in the child's life and the health risks of passive smoking for the new-born.
	Jaakkola, 2001 (53)	The programme included health education material for both pregnant women and their husbands, indicating some level of father involvement.
	Herbert, 2011 (56)	The trial tested if parents' participation in an intervention based on an empowerment ideology and participatory experiences decreased the number of cigarettes smoked in homes. Father involvement in the study consisted of 4 fathers out of 60 participants, which is approximately 7% of the total participants.
	Everett-Murphy, 2010 (58)	Fathers were the target audience for educational materials designed to help support the women in quitting smoking, and actively reading the materials provided as part of the intervention.
**PASSIVE**	Mejdoubi, 2014 (9)	Father involvement is only mentioned in the context of choosing a date for smoking cessation with the pregnant woman and her partner.
	Mcleod, 2004 (12)	Father or partner involvement was only considered in the context of social support for smoking cessation and in terms of whether the partner smoked, which was a factor in the woman's ability to quit smoking.
	Hajek, 2001 (13)	Father involvement in the intervention was limited to the possibility of the woman's partner or friend witnessing her commitment to stop smoking on the last page of the booklet provided.
	Coleman, 2021 (18)	Fathers or partners' smoking status was considered in personalizing the MiQuit intervention for the pregnant participants.
	Britton, 2006 (23)	The study encouraged the involvement of fathers or partners by urging them to accompany pregnant women to subsequent visits and highlighted the importance of their involvement in smoking cessation protocols.
	Lowe, 1998 (29)	Fathers were included in the use of contracts that the subject and her partner were supposed to sign to support the subject's attempt to quit smoking.
	Aveyard, 2005 (31)	Neither the leaflet given to the control group, nor the stage-based manuals directly addressed partner quitting. The only way that these self-help interventions could therefore, influence partners' smoking would be if the partners' motivation to stop was bolstered by the pregnant woman attempting to quit, or the woman shared the manual with her partner.

**NO ROLE**	Panjari, M., 1999 (2)	Women were asked about their partner's smoking status.
	Bailey, 2015 (3)	Not mentioned
	Polańska, 2004 (5)	Women were asked about their partner's smoking status.
	Tappin, 2005 (6)	Not mentioned
	Mcdonnell, 2023 (7)	Women were asked about their partner's smoking status.
	Hegaard, 2003 (8)	Women were asked about their partner's smoking status.
	Tappin, 2015 (10)	Women were asked about their partner's smoking status.
	Tappin, 2022 (11)	Women were asked about their partner's smoking status.
	Dornelas, 2006 (14)	Not mentioned
	Olson, 2019 (15)	Not mentioned
	Gielen, 1997 (16)	Not mentioned
	Rigotti, 2006 (17)	Women were asked about their partner's smoking status and their support level.
	Ershoff, 1999 (19)	Women were asked about their partner's smoking status.
	Walsh, 1997 (20)	Not mentioned
	Secker-Walker, 1994 (22)	Not mentioned
	O'Connor, 1992 (24)	Not mentioned
	Pbert, 2004 (25)	Not mentioned
	Cummins, 2016 (28)	Not mentioned
	Bullock, 2009 (30)	Women were asked about their partner's smoking status and influence was statistically measured.
	Abroms, 2017 (32)	Women were asked about their partner's smoking status.
	Malchodi, 2003 (34)	Not mentioned
	Althabe, 2016 (36)	Women were asked about their partner's smoking status.
	Cope, 2003 (38)	Not mentioned
	Pollak, 2020 (39)	Women were asked about their partner's smoking status.

	Marin-Gomez, 2019 (41)	Not mentioned
	Loukopoulou, 2018 (43)	Women were asked about their partner's smoking status.
	Keten, 2013 (44)	Not mentioned
	Tsoh, 2010 (45)	Not mentioned
	Forinash, 2018 (46)	Not mentioned
	Albrecht, 1998 (48)	Not mentioned
	Hjalmarson, 1991 (50)	Not mentioned
	Windsor, 1985 (51)	Not mentioned
	Alemán, 2016 (52)	Not mentioned
	Price, 1991 (54)	Not mentioned
	Higgins, 2022 (55)	Not mentioned
	Patten, 2020 (57)	Not mentioned

References:

1. Eades SJ, Sanson-Fisher RW, Wenitong M, Panaretto K, D'Este C, Gilligan C, et al. An intensive smoking intervention for pregnant Aboriginal and Torres Strait Islander women: a randomised controlled trial. Med J Aust. 2012;197(1):42-6.
2. Panjari M, Bell R, Bishop S, Astbury J, Rice G, Doery J. A randomized controlled trial of a smoking cessation intervention during pregnancy. Aust N Z J Obstet Gynaecol. 1999;39(3):312-7.
3. Bailey BA. Effectiveness of a Pregnancy Smoking Intervention: The Tennessee Intervention for Pregnant Smokers Program. Health Educ Behav. 2015;42(6):824-31.
4. Moore L, Campbell R, Whelan A, Mills N, Lupton P, Misselbrook E, et al. Self help smoking cessation in pregnancy: cluster randomised controlled trial. BMJ. 2002;325(7377):1383.
5. Polanska K, Hanke W, Sobala W, Lowe JB. Efficacy and effectiveness of the smoking cessation program for pregnant women. Int J Occup Med Environ Health. 2004;17(3):369-77.
6. Tappin DM, Lumsden MA, Gilmour WH, Crawford F, McIntyre D, Stone DH, et al. Randomised controlled trial of home based motivational interviewing by midwives to help pregnant smokers quit or cut down. BMJ. 2005;331(7513):373-7.
7. McDonnell BP, Dicker P, Keogan S, Clancy L, Regan C. Smoking cessation Through Optimization of clinical care in Pregnancy: the STOP pragmatic randomized controlled trial. American Journal of Obstetrics & Gynecology Mfm. 2023;5(1):100763-undefined.
8. Hegaard HK, Kjaergaard H, Moller LF, Wachmann H, Ottesen B. Multimodal intervention raises smoking cessation rate during pregnancy. Acta Obstet Gynecol Scand. 2003;82(9):813-9.
9. Mejdoubi J, van den Heijkant SC, van Leerdam FJ, Crone M, Crijnen A, HiraSing RA. Effects of nurse home visitation on cigarette smoking, pregnancy outcomes and breastfeeding: a randomized controlled trial. Midwifery. 2014;30(6):688-95.
10. Tappin D, Bauld L, Purves D, Boyd K, Sinclair L, MacAskill S, et al. Financial incentives for smoking cessation in pregnancy: randomised controlled trial. BMJ. 2015;350:h134.
11. Tappin D, Sinclair L, Kee F, McFadden M, Robinson-Smith L, Mitchell A, et al. Effect of financial voucher incentives provided with UK stop smoking services on the cessation of smoking in pregnant women (CPIT III): pragmatic, multicentre, single blinded, phase 3, randomised controlled trial. BMJ. 2022;379:e071522.
12. McLeod D, Pullon S, Benn C, Cookson T, Dowell A, Viccars A, et al. Can support and education for smoking cessation and reduction be provided effectively by midwives within primary maternity care? 2004;20(1):37-50.
13. Hajek P, West R, Lee A, Foulds J, Owen L, Eiser JR, et al. Randomized controlled trial of a midwife-delivered brief smoking cessation intervention in pregnancy. Addiction. 2001;96(3):485-94.
14. Dornelas EA, Magnavita J, Beazoglou T, Fischer EH, Oncken C, Lando H, et al. Efficacy and cost-effectiveness of a clinic-based counseling intervention tested in an ethnically diverse sample of pregnant smokers. Patient Educ Couns. 2006;64(1-3):342-9.
15. Olson AL, Boardman MB, Johnson DJ. Smoke-Free Moms: Financial Rewards for Smoking Cessation by Low-Income Rural Pregnant Women. Am J Prev Med. 2019;56(6):852-9.
16. Gielen AC, Windsor R, Faden RR, O'Campo P, Repke J, Davis M. Evaluation of a smoking cessation intervention for pregnant women in an urban prenatal clinic. Health Educ Res. 1997;12(2):247-54.

17. Rigotti NA, Park ER, Regan S, Chang Y, Perry K, Loudin B, et al. Efficacy of telephone counseling for pregnant smokers: a randomized controlled trial. Obstet Gynecol. 2006;108(1):83-92.
18. Coleman T, Clark M, Welch C, Whitemore R, Leonardi-Bee J, Cooper S, et al. Effectiveness of offering tailored text message, self-help smoking cessation support to pregnant women who want information on stopping smoking: MiQuit3 randomised controlled trial and meta-analysis. Addiction. 2021;117(4):1079-94.
19. Ershoff DH, Quinn VP, Boyd NR, Stern J, Gregory M, Wirtschafter D. The Kaiser Permanente prenatal smoking-cessation trial: when more isn't better, what is enough? 1999;17(3):161-8.
20. Walsh RA, Redman S, Brinsmead MW, Byrne JM, Melmeth A. A smoking cessation program at a public antenatal clinic. Am J Public Health. 1997;87(7):1201-4.
21. Stotts AL, Diclemente CC, Dolan-Mullen P. One-to-one: a motivational intervention for resistant pregnant smokers. Addict Behav. 2002;27(2):275-92.
22. Secker-Walker RH, Solomon LJ, Flynn BS, Skelly JM, Lepage SS, Goodwin GD, et al. Individualized smoking cessation counseling during prenatal and early postnatal care. Am J Obstet Gynecol. 1994;171(5):1347-55.
23. Britton GRA. The effectiveness of a nurse-managed perinatal smoking cessation program implemented in a rural county 2006.
24. O'Connor AM, Davies BL, Dulberg CS, Buhler PL, Nadon C, McBride BH, et al. Effectiveness of a pregnancy smoking cessation program. J Obstet Gynecol Neonatal Nurs. 1992;21(5):385-92.
25. Pbert L, Ockene JK, Zapka J, Ma Y, Goins KV, Oncken C, et al. A community health center smoking-cessation intervention for pregnant and postpartum women. Am J Prev Med. 2004;26(5):377-85.
26. Luk TT, Lam TH, Leung WC, Leung KY, Cheung KW, Kwa C, et al. Brief Advice, Nicotine Replacement Therapy Sampling, and Active Referral for Expectant Fathers Who Smoke Cigarettes: A Randomized Clinical Trial. JAMA Intern Med. 2021;181(8):1081-9.
27. de Vries H, Bakker M, Mullen PD, van Breukelen G. The effects of smoking cessation counseling by midwives on Dutch pregnant women and their partners. Patient Educ Couns. 2006;63(1-2):177-87.
28. Cummins SE, Tedeschi GJ, Anderson CM, Zhu SH. Telephone Intervention for Pregnant Smokers: A Randomized Controlled Trial. Am J Prev Med. 2016;51(3):318-26.
29. Lowe JB, Balanda KP, Clare G. Evaluation of antenatal smoking cessation programs for pregnant women. Aust N Z J Public Health. 1998;22(1):55-9.
30. Bullock L, Everett KD, Mullen PD, Geden E, Longo DR, Madsen R. Baby BEEP: A randomized controlled trial of nurses' individualized social support for poor rural pregnant smokers. Matern Child Health J. 2009;13(3):395-406.
31. Aveyard P, Lawrence T, Evans O, Cheng KK. The influence of in-pregnancy smoking cessation programmes on partner quitting and women's social support mobilization: a randomized controlled trial [ISRCTN89131885]. BMC Public Health. 2005;5:80.
32. Abroms LC, Johnson PR, Leavitt LE, Cleary SD, Bushar J, Brandon TH, et al. A Randomized Trial of Text Messaging for Smoking Cessation in Pregnant Women. Am J Prev Med. 2017;53(6):781-90.
33. McBride CM, Baucom DH, Peterson BL, Pollak KI, Palmer C, Westman E, et al. Prenatal and postpartum smoking abstinence a partner-assisted approach. Am J Prev Med. 2004;27(3):232-8.
34. Malchodi CS, Oncken C, Dornelas EA, Caramanica L, Gregonis E, Curry SL. The effects of peer counseling on smoking cessation and reduction. Obstet Gynecol. 2003;101(3):504-10.

35. Pollak KI, Lyna P, Bilheimer AK, Gordon KC, Peterson BL, Gao X, et al. Efficacy of a couple-based randomized controlled trial to help Latino fathers quit smoking during pregnancy and postpartum: the Parejas trial. Cancer Epidemiol Biomarkers Prev. 2015;24(2):379-85.
36. Althabe F, Aleman A, Berrueta M, Morello P, Gibbons L, Colomar M, et al. A Multifaceted Strategy to Implement Brief Smoking Cessation Counseling During Antenatal Care in Argentina and Uruguay: A Cluster Randomized Trial. Nicotine Tob Res. 2016;18(5):1083-92.
37. McBride CM BD, Peterson BL et al. Partner-assisted support intervention did not improve smoking abstinence among women during and after pregnancy. Evidence-based Healthcare and Public Health. 2005;9(3):233-4.
38. Cope GF, Nayyar P, Holder R. Feedback from a point-of-care test for nicotine intake to reduce smoking during pregnancy. Ann Clin Biochem. 2003;40(Pt 6):674-9.
39. Pollak KI, Lyna P, Gao X, Noonan D, Bejarano Hernandez S, Subudhi S, et al. Efficacy of a Texting Program to Promote Cessation Among Pregnant Smokers: A Randomized Control Trial. Nicotine Tob Res. 2020;22(7):1187-94.
40. Yang L, Tong EK, Mao Z, Hu TW, Lee AH. A Clustered Randomized Controlled Trial to Reduce Secondhand Smoke Exposure Among Nonsmoking Pregnant Women in Sichuan Province, China. Nicotine Tob Res. 2016;18(5):1163-70.
41. Marin-Gomez FX, Garcia-Moreno Marchan R, Mayos-Fernandez A, Flores-Mateo G, Granado-Font E, Barrera Uriarte ML, et al. Exploring Efficacy of a Serious Game (Tobbstop) for Smoking Cessation During Pregnancy: Randomized Controlled Trial. JMIR Serious Games. 2019;7(3):e14381.
42. Xia W, Li HCW, Cai W, Song P, Zhou X, Lam KWK, et al. Effectiveness of a video-based smoking cessation intervention focusing on maternal and child health in promoting quitting among expectant fathers in China: A randomized controlled trial. PLoS Med. 2020;17(9):e1003355.
43. Loukopoulou AN, Vardavas CI, Farmakides G, Rosolymos C, Chrelias C, Tzatzarakis M, et al. Counselling for smoking cessation during pregnancy reduces tobacco-specific nitrosamine (NNAL) concentrations: A randomized controlled trial. Eur J Midwifery. 2018;2:14.
44. Keten E, Golbasi Z. The Effect of Three Different Smoking Cessation Intervention for Smoker Pregnant Women. TAF Preventive Medicine Bulletin. 2013;12(5):553-62.
45. Tsoh JY, Kohn MA, Gerbert B. Promoting smoking cessation in pregnancy with Video Doctor plus provider cueing: a randomized trial. Acta Obstet Gynecol Scand. 2010;89(4):515-23.
46. Forinash AB, Yancey A, Chamness D, Koerner J, Inteso C, Miller C, et al. Smoking Cessation Following Text Message Intervention in Pregnant Women. Ann Pharmacother. 2018;52(11):1109-16.
47. Soltani F, Barzegar F, Sangestani G, Roshanaii G, Maleki A. The effectiveness of family counselling on reducing exposure to secondhand smoke at home among pregnant women in Iran. Tob Prev Cessat. 2019;5:41.
48. Albrecht S, Payne L, Stone CA, Reynolds MD. A preliminary study of the use of peer support in smoking cessation programs for pregnant adolescents. J Am Acad Nurse Pract. 1998;10(3):119-25.
49. Stanton WR, Lowe JB, Moffatt J, Del Mar CB. Randomised control trial of a smoking cessation intervention directed at men whose partners are pregnant. Prev Med. 2004;38(1):6-9.
50. Hjalmarson AI, Hahn L, Svanberg B. Stopping smoking in pregnancy: effect of a self-help manual in controlled trial. Br J Obstet Gynaecol. 1991;98(3):260-4.
51. Windsor RA, Cutter G, Morris J, Reese Y, Manzella B, Bartlett EE, et al. The effectiveness of smoking cessation methods for smokers in public health maternity clinics: a randomized trial. Am J Public Health. 1985;75(12):1389-92.

52. Aleman A, Morello P, Colomar M, Llambi L, Berrueta M, Gibbons L, et al. Brief Counseling on Secondhand Smoke Exposure in Pregnant Women in Argentina and Uruguay. Int J Environ Res Public Health. 2016;14(1).
53. Jaakkola N, Zahlsen K, Jaakkola JJ. Effects of a population-based smoking cessation programme on smoking in pregnancy. Eur J Public Health. 2001;11(4):446-9.
54. Price JH, Krol RA, Desmond SM, Losh DP, Roberts SM, Snyder FF. Comparison of three antismoking interventions among pregnant women in an urban setting: a randomized trial. 1991;68(2):595-604.
55. Higgins ST, Nighbor TD, Kurti AN, Heil SH, Slade EP, Shepard DS, et al. Randomized Controlled Trial Examining the Efficacy of Adding Financial Incentives to Best practices for Smoking Cessation Among pregnant and Newly postpartum Women. Prev Med. 2022;165(Pt B):107012.
56. Herbert RJ, Gagnon AJ, O'Loughlin JL, Rennick JE. Testing an empowerment intervention to help parents make homes smoke-free: a randomized controlled trial. J Community Health. 2011;36(4):650-7.
57. Patten CA, Lando HA, Desnoyers CA, Bock MJ, Alexie L, Decker PA, et al. Healthy Pregnancies Project: Cluster Randomized Controlled Trial of a Community Intervention to Reduce Tobacco Use among Alaska Native Women. Int J Environ Res Public Health. 2020;17(24).
58. Everett-Murphy K, Steyn K, Mathews C, Petersen Z, Odendaal H, Gwebushe N, et al. The effectiveness of adapted, best practice guidelines for smoking cessation counseling with disadvantaged, pregnant smokers attending public sector antenatal clinics in Cape Town, South Africa. Acta Obstet Gynecol Scand. 2010;89(4):478-89.

7. META-ANALYSIS RESULTS

I. FOREST PLOTS OF RESULTS

Fig 1. Forest plot of the meta-analysis of random effects on smoking cessation of individual-based interventions

Study or Subgroup	Experimental Events	Experimental Total	Control Events	Control Total	Weight	Odds ratio IV, Random, 95% CI
Althabe 2016	52	476	34	407	3.9%	1.35 [0.85 , 2.12]
Bailey 2015	417	1486	45	461	4.2%	3.61 [2.60 , 5.00]
Britton 2006	19	66	21	69	3.0%	0.92 [0.44 , 1.94]
Bullock 2009	22	129	22	128	3.3%	0.99 [0.52 , 1.90]
Coleman 2021	26	501	23	501	3.5%	1.14 [0.64 , 2.02]
Cope 2003	22	109	4	83	2.1%	4.99 [1.65 , 15.12]
Cummins 2016	120	584	119	588	4.3%	1.02 [0.77 , 1.35]
de Vries 2006	27	141	42	177	3.6%	0.76 [0.44 , 1.31]
Dornelas 2006	15	53	5	52	2.1%	3.71 [1.24 , 11.13]
Eades 2012	11	148	4	115	2.0%	2.23 [0.69 , 7.19]
Ershoff 1999	14	60	19	61	2.8%	0.67 [0.30 , 1.51]
Everett-Murphy 2010	30	358	2	269	1.5%	12.21 [2.89 , 51.56]
Forinash 2018	8	14	5	16	1.4%	2.93 [0.66 , 13.09]
Gielen 1997	12	193	11	198	2.7%	1.13 [0.48 , 2.62]
Higgins 2022	30	81	9	88	2.8%	5.16 [2.27 , 11.77]
Hjalmarson 1991	134	444	51	209	4.1%	1.34 [0.92 , 1.95]
Keten 2013	29	41	18	43	2.6%	3.36 [1.36 , 8.30]
Loukopoulou 2018	19	42	9	42	2.5%	3.03 [1.17 , 7.87]
Lowe 1998	4	44	0	34	0.5%	7.67 [0.40 , 147.48]
Malchodi 2003	16	67	16	75	2.9%	1.16 [0.53 , 2.54]
McDonnell 2023	21	219	6	211	2.5%	3.62 [1.43 , 9.17]
Mejdoubi 2014	162	237	154	223	4.1%	0.97 [0.65 , 1.44]
Moore 2002	113	600	144	695	4.4%	0.89 [0.67 , 1.17]
Patten 2020	27	188	29	164	3.5%	0.78 [0.44 , 1.38]
Pbert 2004	20	80	14	114	3.0%	2.38 [1.12 , 5.06]
Polanska 2004	126	261	60	181	4.1%	1.88 [1.27 , 2.79]
Pollak 2015	55	173	59	175	3.9%	0.92 [0.59 , 1.43]
Secker-Walker 1994	20	188	20	226	3.3%	1.23 [0.64 , 2.35]
Stotts 2002	20	47	29	86	3.1%	1.46 [0.70 , 3.02]
Tappin 2005	17	351	19	411	3.2%	1.05 [0.54 , 2.05]
Tappin 2015	69	306	26	303	3.8%	3.10 [1.91 , 5.03]
Tsoh 2010	6	23	2	19	1.2%	3.00 [0.53 , 17.02]
Walsh 1997	17	127	7	125	2.5%	2.61 [1.04 , 6.52]
Windsor 1985	14	102	2	104	1.4%	8.11 [1.79 , 36.68]
**Total (95% CI)**		**7939**		**6653**	**100.0%**	**1.66 [1.34 , 2.06]**
Total events:	1714		1030			

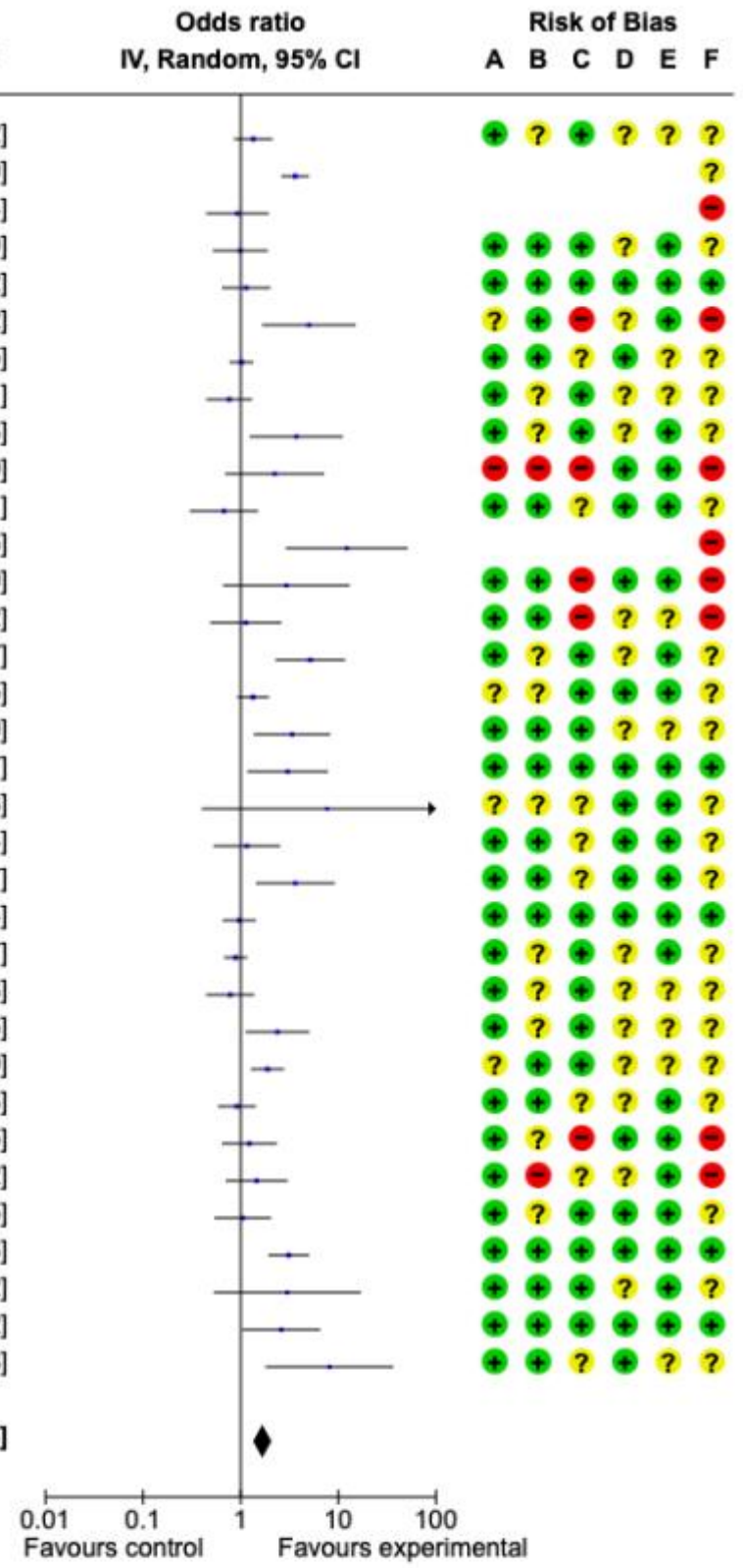

Heterogeneity: Tau² = 0.26; Chi² = 131.81, df = 33 (P < 0.00001); I² = 75%
Test for overall effect: Z = 4.60 (P < 0.00001)
Test for subgroup differences: Not applicable

Risk of bias legend
(A) Bias arising from the randomization process
(B) Bias due to deviations from intended interventions
(C) Bias due to missing outcome data
(D) Bias in measurement of the outcome
(E) Bias in selection of the reported result
(F) Overall bias

Fig.2. Forest plot of the meta-analysis of random effects on smoking cessation of group-based interventions

Study or Subgroup	Experimental Events	Experimental Total	Control Events	Control Total	Weight	Odds ratio IV, Random, 95% CI	Risk of Bias A	B	C	D	E	F
Albrecht 1998	3	10	5	10	4.4%	0.43 [0.07 , 2.68]	?	?	−	?	+	−
Hegaard 2003	23	327	7	320	14.9%	3.38 [1.43 , 8.00]						−
Jaakkola 2001	58	306	22	152	25.4%	1.38 [0.81 , 2.36]						−
McLeod 2004	19	67	13	56	16.0%	1.31 [0.58 , 2.96]	+	?	+	?	?	?
O'Connor 1992	15	101	5	101	11.2%	3.35 [1.17 , 9.60]	+	+	+	+	+	+
Panjari 1999	40	339	38	393	28.2%	1.25 [0.78 , 2.00]	+	?	+	?	+	?
**Total (95% CI)**		**1150**		**1032**	**100.0%**	**1.60 [1.06 , 2.39]**						
Total events:	158		90									

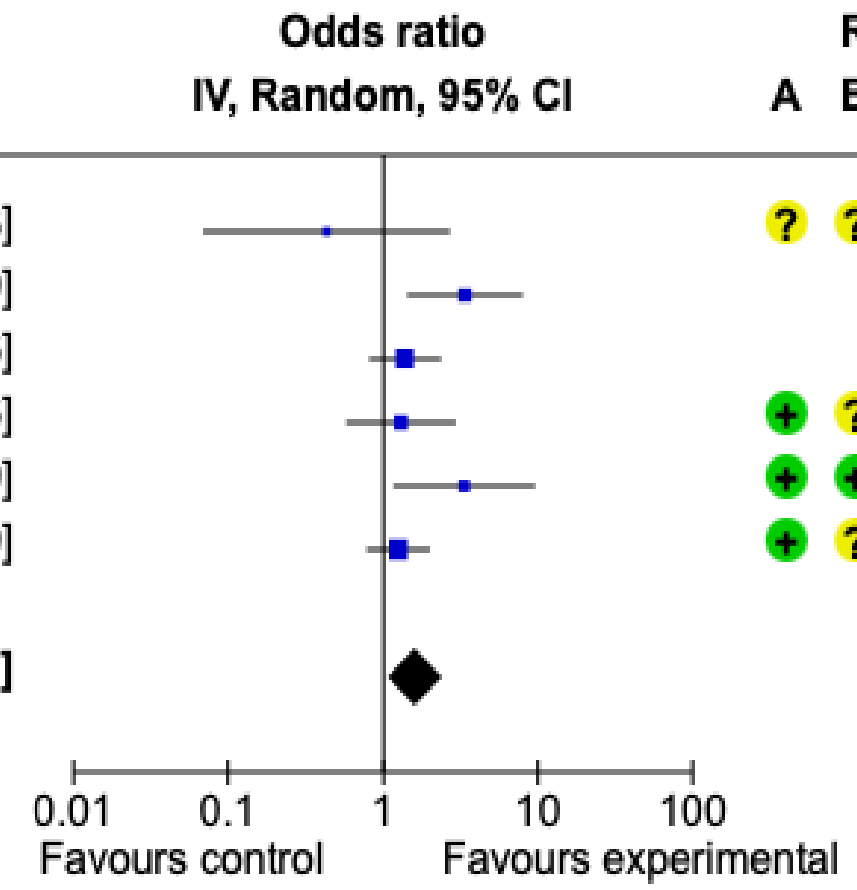

Heterogeneity: Tau² = 0.09; Chi² = 8.23, df = 5 (P = 0.14); I² = 39%

Test for overall effect: Z = 2.26 (P = 0.02)

Test for subgroup differences: Not applicable

Risk of bias legend

(A) Bias arising from the randomization process

(B) Bias due to deviations from intended interventions

(C) Bias due to missing outcome data

(D) Bias in measurement of the outcome

(E) Bias in selection of the reported result

(F) Overall bias

Fig. 3. Forest plot of the meta-analysis of random effects on smoking cessation of telephone-based interventions

Study or Subgroup	Experimental Events	Experimental Total	Control Events	Control Total	Weight	Odds ratio IV, Random, 95% CI	Risk of Bias A	B	C	D	E	F
Bailey 2015	417	1486	45	461	10.8%	3.61 [2.60 , 5.00]						?
Britton 2006	19	66	21	69	8.3%	0.92 [0.44 , 1.94]						−
Cummins 2016	120	584	119	588	11.0%	1.02 [0.77 , 1.35]	+	+	?	+	?	?
Ershoff 1999	14	60	19	61	7.9%	0.67 [0.30 , 1.51]	+	+	?	+	+	?
Higgins 2022	30	81	9	88	7.8%	5.16 [2.27 , 11.77]	+	?	+	?	+	?
Malchodi 2003	16	67	16	75	8.0%	1.16 [0.53 , 2.54]	+	+	?	+	+	?
McBride 2004	61	193	60	198	10.2%	1.06 [0.69 , 1.63]	+	+	+	?	+	?
McBride 2005	41	195	30	195	9.7%	1.46 [0.87 , 2.46]	?	?	?	−	+	−
O'Connor 1992	15	101	5	101	6.4%	3.35 [1.17 , 9.60]	+	+	+	+	+	+
Patten 2020	27	188	29	164	9.4%	0.78 [0.44 , 1.38]	+	?	+	?	?	?
Tappin 2022	126	471	58	470	10.7%	2.59 [1.84 , 3.65]	+	+	+	+	+	+
**Total (95% CI)**		**3492**		**2470**	**100.0%**	**1.55 [1.04 , 2.31]**						
Total events:	886		411									

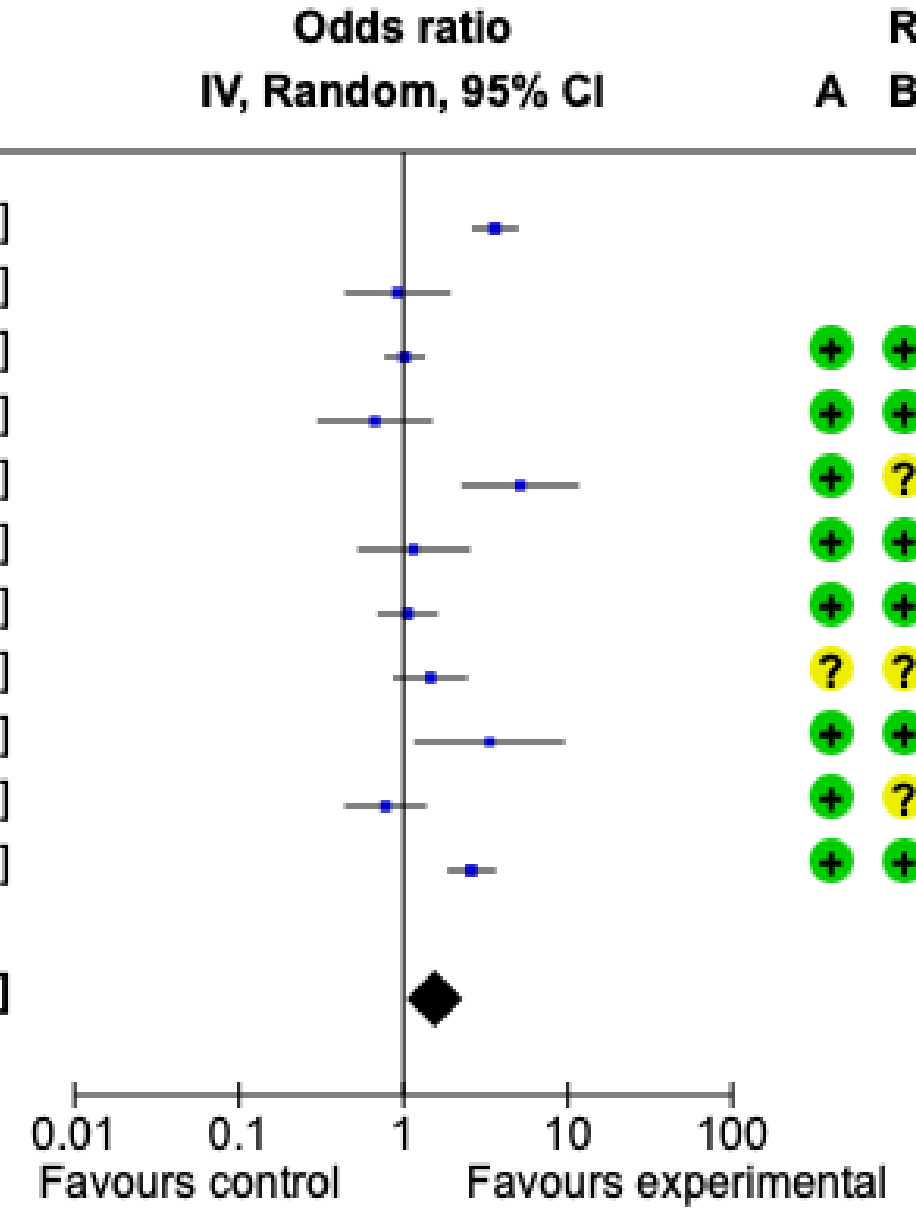

Heterogeneity: $Tau^2 = 0.35$; $Chi^2 = 67.39$, df = 10 ($P < 0.00001$); $I^2 = 85\%$

Test for overall effect: Z = 2.17 (P = 0.03)

Test for subgroup differences: Not applicable

Risk of bias legend

(A) Bias arising from the randomization process

(B) Bias due to deviations from intended interventions

(C) Bias due to missing outcome data

(D) Bias in measurement of the outcome

(E) Bias in selection of the reported result

(F) Overall bias

Fig. 4. Forest plot of the meta-analysis of random effects on smoking cessation of home-based interventions

Study or Subgroup	Experimental Events	Experimental Total	Control Events	Control Total	Weight	Odds ratio IV, Random, 95% CI	A	B	C	D	E	F
Keten 2013	29	41	18	43	16.6%	3.36 [1.36 , 8.30]	+	+	+	?	?	?
Mejdoubi 2014	162	237	154	223	30.6%	0.97 [0.65 , 1.44]	+	+	+	+	+	+
Polanska 2004	126	261	60	181	30.6%	1.88 [1.27 , 2.79]	?	+	+	?	?	?
Tappin 2005	17	351	19	411	22.2%	1.05 [0.54 , 2.05]	+	?	+	+	+	?
**Total (95% CI)**		**890**		**858**	**100.0%**	**1.48 [0.91 , 2.42]**						
Total events:	334		251									

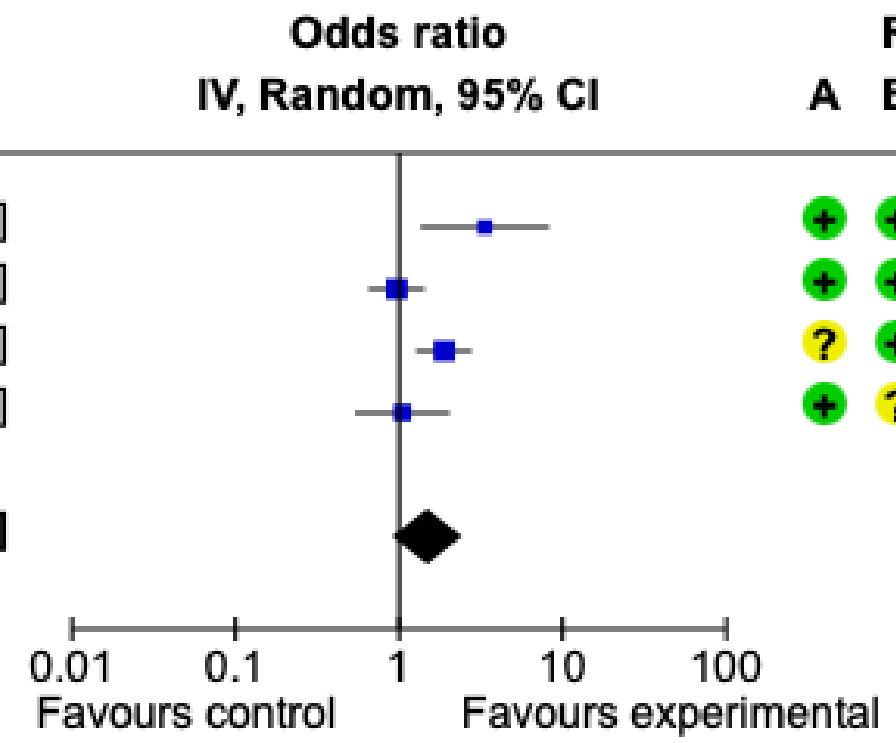

Heterogeneity: Tau² = 0.16; Chi² = 9.83, df = 3 (P = 0.02); I² = 69%
Test for overall effect: Z = 1.58 (P = 0.11)
Test for subgroup differences: Not applicable

Risk of bias legend
(A) Bias arising from the randomization process
(B) Bias due to deviations from intended interventions
(C) Bias due to missing outcome data
(D) Bias in measurement of the outcome
(E) Bias in selection of the reported result
(F) Overall bias

Fig. 5. Forest plot of the meta-analysis of random effects on smoking cessation of written health education materials

Study or Subgroup	Experimental Events	Experimental Total	Control Events	Control Total	Weight	Odds ratio IV, Random, 95% CI	A	B	C	D	E	F
Althabe 2016	52	476	34	407	15.1%	1.35 [0.85 , 2.12]	+	?	+	?	?	?
de Vries 2006	27	141	42	177	13.4%	0.76 [0.44 , 1.31]	+	?	+	?	?	?
Everett-Murphy 2010	30	358	2	269	4.2%	12.21 [2.89 , 51.56]						−
Gielen 1997	12	193	11	198	8.8%	1.13 [0.48 , 2.62]	+	+	−	?	?	−
Hajek 2001	17	545	15	575	10.7%	1.20 [0.59 , 2.43]	+	?	?	+	+	?
Jaakkola 2001	58	306	22	152	13.6%	1.38 [0.81 , 2.36]						−
O'Connor 1992	15	101	5	101	6.7%	3.35 [1.17 , 9.60]	+	+	+	+	+	+
Polanska 2004	126	261	60	181	16.3%	1.88 [1.27 , 2.79]	?	+	+	?	?	?
Rigotti 2006	21	209	16	222	11.1%	1.44 [0.73 , 2.84]	+	+	+	+	+	+
**Total (95% CI)**		**2590**		**2282**	**100.0%**	**1.51 [1.09 , 2.09]**						
Total events:	358		207									

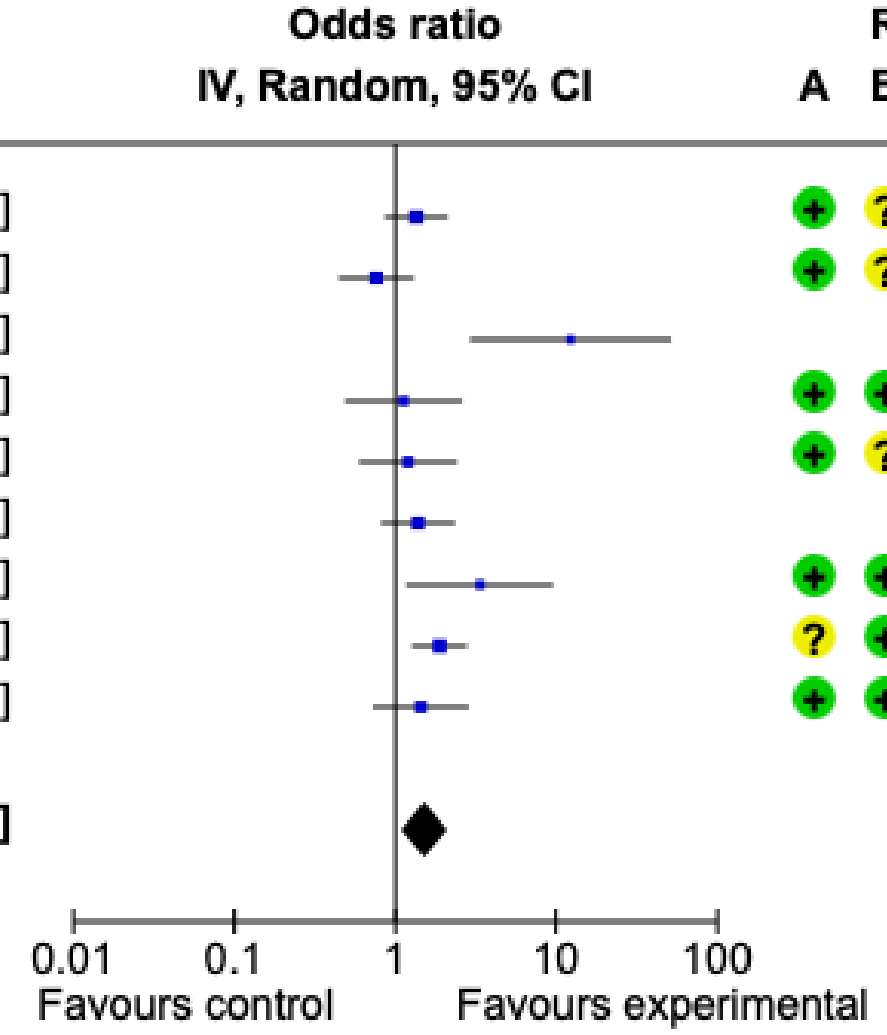

Heterogeneity: Tau² = 0.13; Chi² = 18.66, df = 8 (P = 0.02); I² = 57%
Test for overall effect: Z = 2.46 (P = 0.01)
Test for subgroup differences: Not applicable

Risk of bias legend
(A) Bias arising from the randomization process
(B) Bias due to deviations from intended interventions
(C) Bias due to missing outcome data
(D) Bias in measurement of the outcome
(E) Bias in selection of the reported result
(F) Overall bias

Fig. 6. Forest plot of the meta-analysis of random effects on smoking cessation of text message services

Study or Subgroup	Experimental Events	Experimental Total	Control Events	Control Total	Weight	Odds ratio IV, Random, 95% CI	A	B	C	D	E	F
Abroms 2017	32	250	25	247	41.5%	1.30 [0.75 , 2.27]	+	+	+	+	+	+
Coleman 2021	26	501	23	501	38.7%	1.14 [0.64 , 2.02]	+	+	+	+	+	+
Forinash 2018	8	14	5	16	5.7%	2.93 [0.66 , 13.09]	+	+	−	+	+	−
Pollak 2020	9	154	9	160	14.1%	1.04 [0.40 , 2.70]	+	+	+	+	+	+
**Total (95% CI)**		**919**		**924**	**100.0%**	**1.25 [0.88 , 1.79]**						
Total events:	75		62									

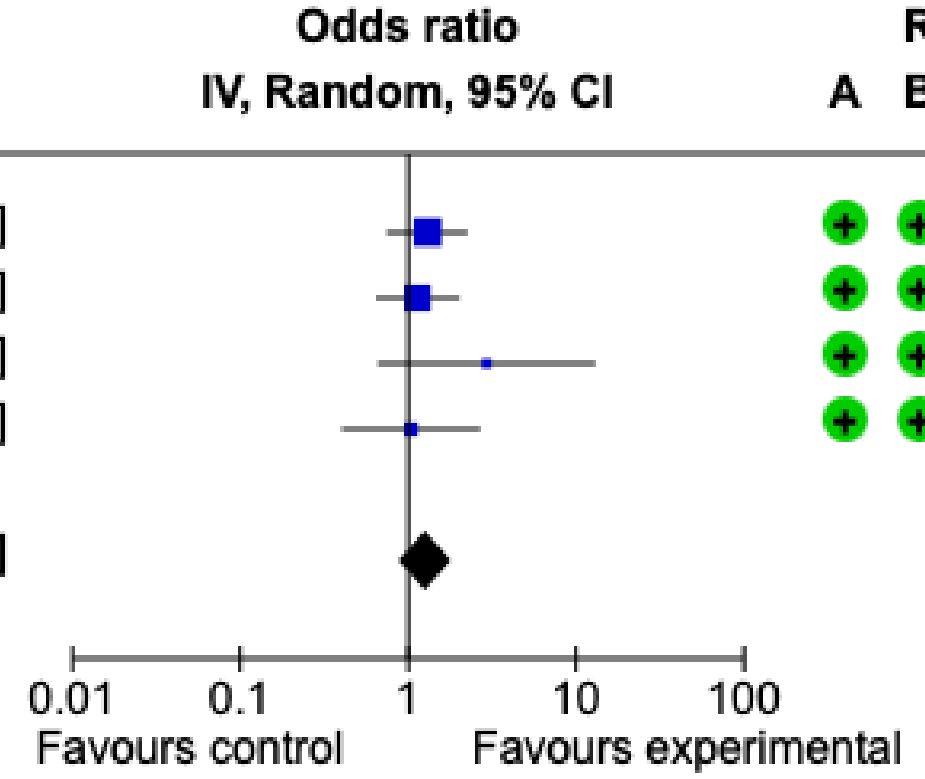

Heterogeneity: $Tau^2 = 0.00$; $Chi^2 = 1.51$, df = 3 (P = 0.68); $I^2 = 0\%$
Test for overall effect: Z = 1.24 (P = 0.21)
Test for subgroup differences: Not applicable

Risk of bias legend
(A) Bias arising from the randomization process
(B) Bias due to deviations from intended interventions
(C) Bias due to missing outcome data
(D) Bias in measurement of the outcome
(E) Bias in selection of the reported result
(F) Overall bias

Fig. 7. Forest plot of the meta-analysis of random effects on smoking cessation of peer-support approach

Study or Subgroup	Experimental Events	Experimental Total	Control Events	Control Total	Weight	Odds ratio IV, Random, 95% CI	Risk of Bias A	B	C	D	E	F
Albrecht 1998	3	10	5	10	11.4%	0.43 [0.07 , 2.68]	?	?	−	?	+	−
Everett-Murphy 2010	30	358	2	269	15.1%	12.21 [2.89 , 51.56]						−
Gielen 1997	12	193	11	198	22.9%	1.13 [0.48 , 2.62]	+	+	−	?	?	−
Malchodi 2003	16	67	16	75	23.7%	1.16 [0.53 , 2.54]	+	+	?	+	+	?
Patten 2020	27	188	29	164	26.9%	0.78 [0.44 , 1.38]	+	?	+	?	?	?
**Total (95% CI)**		**816**		**716**	**100.0%**	**1.32 [0.61 , 2.86]**						
Total events:	88		63									

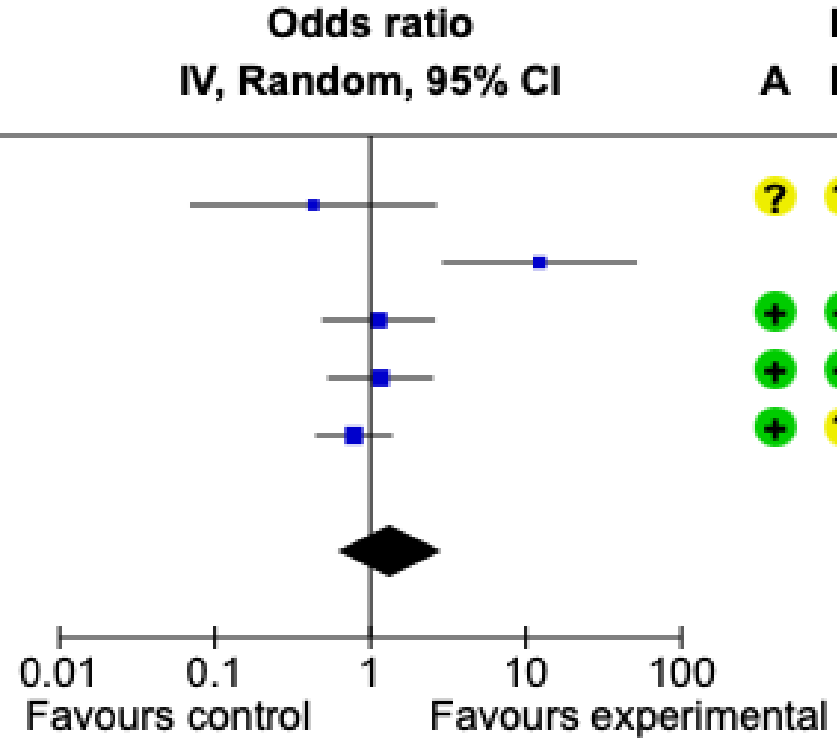

Heterogeneity: $Tau^2 = 0.50$; $Chi^2 = 13.14$, df = 4 (P = 0.01); $I^2 = 70\%$
Test for overall effect: Z = 0.70 (P = 0.48)
Test for subgroup differences: Not applicable

Risk of bias legend
(A) Bias arising from the randomization process
(B) Bias due to deviations from intended interventions
(C) Bias due to missing outcome data
(D) Bias in measurement of the outcome
(E) Bias in selection of the reported result
(F) Overall bias

Fig. 8. Forest plot of the meta-analysis of random effects on smoking cessation of self-help approach

Study or Subgroup	Experimental Events	Experimental Total	Control Events	Control Total	Weight	Odds ratio IV, Random, 95% CI	Risk of Bias A	B	C	D	E	F
Bailey 2015	417	1486	45	461	11.0%	3.61 [2.60 , 5.00]						?
Coleman 2021	26	501	23	501	9.9%	1.14 [0.64 , 2.02]	+	+	+	+	+	+
Cope 2003	22	109	4	83	7.0%	4.99 [1.65 , 15.12]	?	+	−	?	+	−
Ershoff 1999	14	60	19	61	8.6%	0.67 [0.30 , 1.51]	+	+	?	+	+	?
Gielen 1997	12	193	11	198	8.4%	1.13 [0.48 , 2.62]	+	+	−	?	?	−
Hajek 2001	17	545	17	575	9.3%	1.06 [0.53 , 2.09]	+	?	?	+	+	?
Keten 2013	29	41	18	43	8.1%	3.36 [1.36 , 8.30]	+	+	+	?	?	?
Lowe 1998	4	44	0	34	2.0%	7.67 [0.40 , 147.48]	?	?	?	+	+	?
Malchodi 2003	16	67	16	75	8.7%	1.16 [0.53 , 2.54]	+	+	?	+	+	?
Moore 2002	113	600	144	695	11.2%	0.89 [0.67 , 1.17]	+	?	+	?	+	?
O'Connor 1992	15	101	5	101	7.3%	3.35 [1.17 , 9.60]	+	+	+	+	+	+
Price 1991	4	46	1	24	3.1%	2.19 [0.23 , 20.77]	+	+	+	?	?	?
Windsor 1985	14	102	2	104	5.2%	8.11 [1.79 , 36.68]	+	+	?	+	?	?
**Total (95% CI)**		**3895**		**2955**	**100.0%**	**1.84 [1.16 , 2.92]**						
Total events:	703		305									

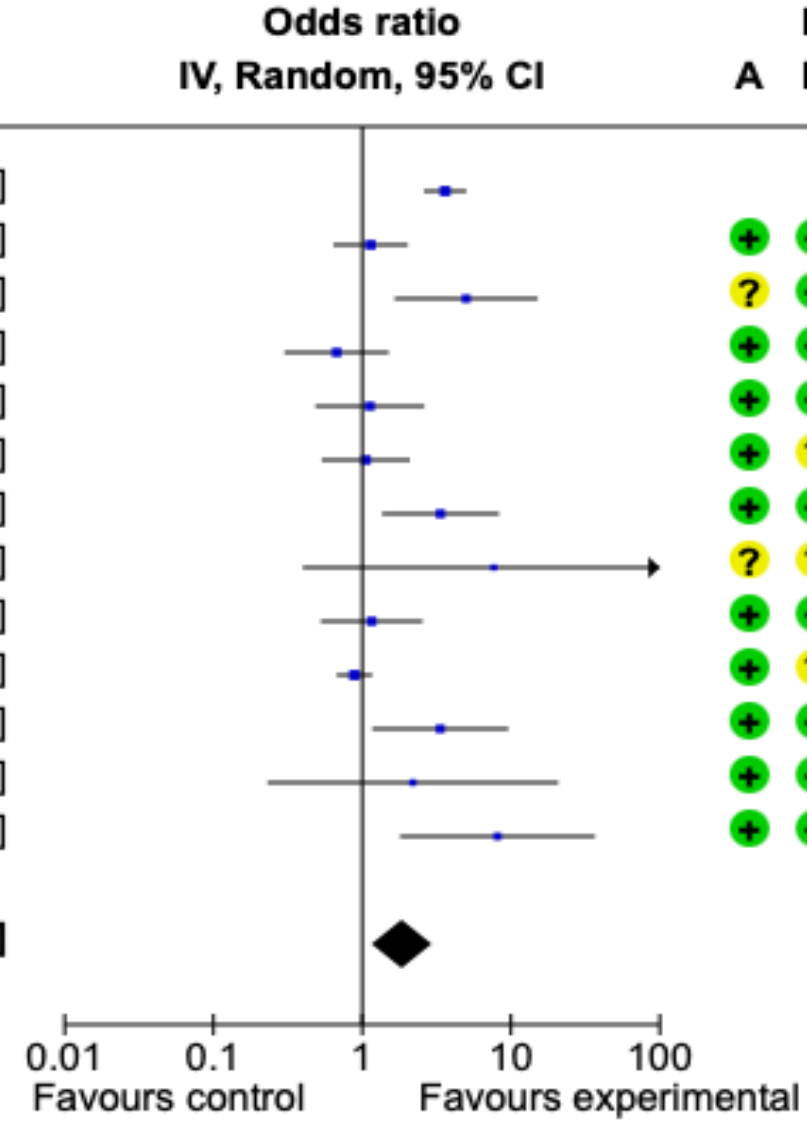

Heterogeneity: Tau² = 0.48; Chi² = 63.77, df = 12 ($P < 0.00001$); I² = 81%
Test for overall effect: Z = 2.57 (P = 0.01)
Test for subgroup differences: Not applicable

Risk of bias legend
(A) Bias arising from the randomization process
(B) Bias due to deviations from intended interventions
(C) Bias due to missing outcome data
(D) Bias in measurement of the outcome
(E) Bias in selection of the reported result
(F) Overall bias

Fig. 9. Forest plot of the meta-analysis of random effects on smoking cessation of face-to-face approach

Study or Subgroup	Experimental Events	Experimental Total	Control Events	Control Total	Weight	Odds ratio IV, Random, 95% CI	Risk of Bias A	B	C	D	E	F
Albrecht 1998	3	10	5	10	1.2%	0.43 [0.07 , 2.68]	?	?	−	?	+	−
Althabe 2016	52	476	34	407	4.8%	1.35 [0.85 , 2.12]	+	?	+	?	?	?
Bailey 2015	417	1486	45	461	5.3%	3.61 [2.60 , 5.00]						?
Britton 2006	19	66	21	69	3.6%	0.92 [0.44 , 1.94]						−
Bullock 2009	22	129	22	128	4.0%	0.99 [0.52 , 1.90]	+	+	+	?	+	?
Cope 2003	22	109	4	83	2.5%	4.99 [1.65 , 15.12]	?	+	−	?	+	−
Dornelas 2006	15	53	5	52	2.5%	3.71 [1.24 , 11.13]	+	?	+	?	+	?
Eades 2012	11	148	4	115	2.3%	2.23 [0.69 , 7.19]	−	−	−	+	+	−
Everett-Murphy 2010	30	358	2	269	1.8%	12.21 [2.89 , 51.56]						−
Gielen 1997	12	193	11	198	3.3%	1.13 [0.48 , 2.62]	+	+	−	?	?	−
Hajek 2001	17	545	17	575	3.9%	1.06 [0.53 , 2.09]	+	?	?	+	+	?
Hegaard 2003	23	327	7	320	3.2%	3.38 [1.43 , 8.00]						−
Jaakkola 2001	58	306	22	152	4.5%	1.38 [0.81 , 2.36]						−
Keten 2013	29	41	18	43	3.1%	3.36 [1.36 , 8.30]	+	+	+	?	?	?
Loukopoulou 2018	19	42	9	42	2.9%	3.03 [1.17 , 7.87]	+	+	+	+	+	+
Lowe 1998	4	44	0	34	0.5%	7.67 [0.40 , 147.48]	?	?	?	+	+	?
Malchodi 2003	16	67	16	75	3.5%	1.16 [0.53 , 2.54]	+	+	?	+	+	?
McDonnell 2023	21	219	6	211	3.0%	3.62 [1.43 , 9.17]	+	+	?	+	+	?
Mejdoubi 2014	162	237	154	223	5.0%	0.97 [0.65 , 1.44]	+	+	+	+	+	+
O'Connor 1992	15	101	5	101	2.6%	3.35 [1.17 , 9.60]	+	+	+	+	+	+
Olson 2019	24	66	20	68	3.7%	1.37 [0.67 , 2.83]						?
Pbert 2004	20	80	14	114	3.6%	2.38 [1.12 , 5.06]	+	?	+	?	?	?
Polanska 2004	126	261	60	181	5.0%	1.88 [1.27 , 2.79]	?	+	+	?	?	?
Pollak 2015	55	173	59	175	4.8%	0.92 [0.59 , 1.43]	+	+	?	?	+	?
Secker-Walker 1994	20	188	20	226	4.0%	1.23 [0.64 , 2.35]	+	?	−	+	+	−
Tappin 2005	17	351	19	411	3.9%	1.05 [0.54 , 2.05]	+	?	+	+	+	?
Tappin 2015	69	306	26	303	4.7%	3.10 [1.91 , 5.03]	+	+	+	+	+	+
Tappin 2022	126	471	58	470	5.2%	2.59 [1.84 , 3.65]	+	+	+	+	+	+
Windsor 1985	14	102	2	104	1.7%	8.11 [1.79 , 36.68]	+	+	?	+	?	?
**Total (95% CI)**		**6955**		**5620**	**100.0%**	**1.89 [1.50 , 2.37]**						
Total events:	1438		685									

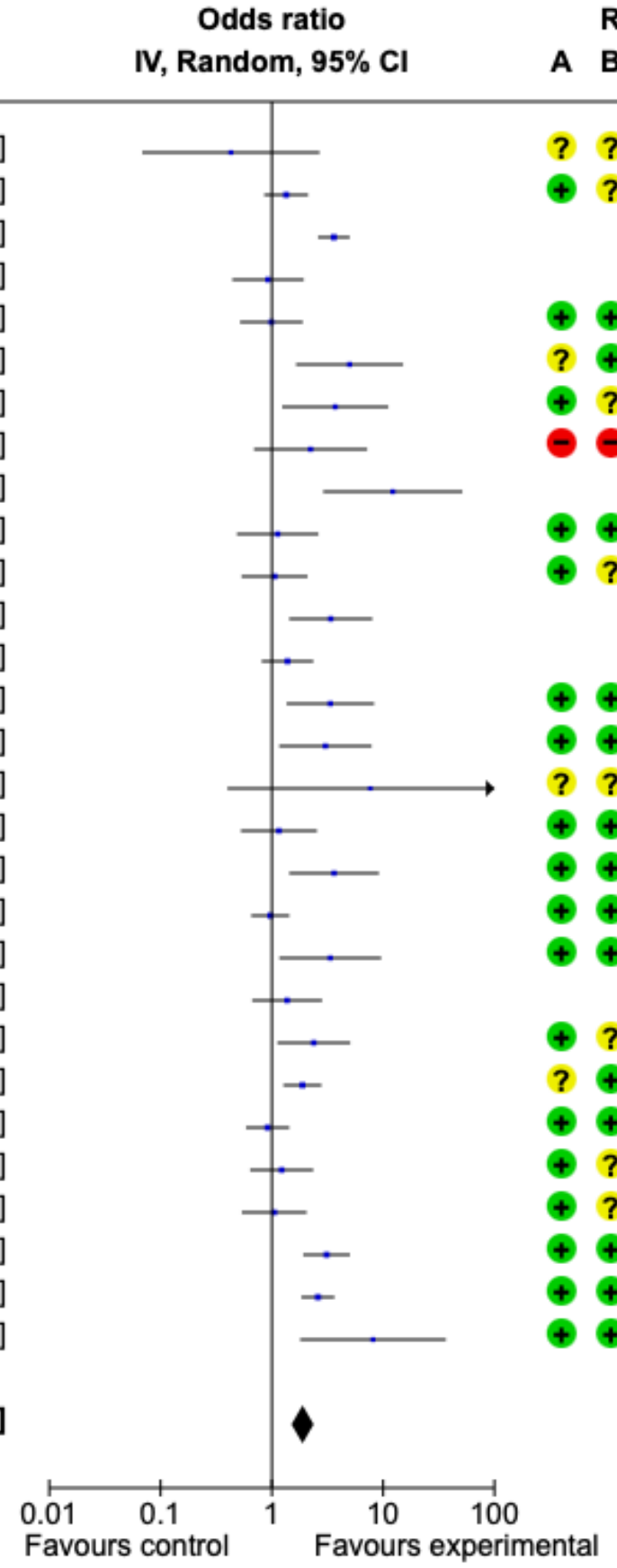

Heterogeneity: Tau² = 0.23; Chi² = 90.64, df = 28 (P < 0.00001); I² = 69%
Test for overall effect: Z = 5.44 (P < 0.00001)
Test for subgroup differences: Not applicable

Risk of bias legend
(A) Bias arising from the randomization process
(B) Bias due to deviations from intended interventions
(C) Bias due to missing outcome data
(D) Bias in measurement of the outcome
(E) Bias in selection of the reported result
(F) Overall bias

Fig. 10. Forest plot of the meta-analysis of random effects on smoking cessation of technology-mediated approach

Study or Subgroup	Experimental Events	Experimental Total	Control Events	Control Total	Weight	Odds ratio IV, Random, 95% CI	A	B	C	D	E	F
**5.2.1 Tailored educational video**												
de Vries 2006	27	141	42	177	8.2%	0.76 [0.44 , 1.31]	+	?	+	?	?	?
Hjalmarson 1991	134	444	51	209	9.9%	1.34 [0.92 , 1.95]	?	?	+	+	+	?
McBride 2004	61	193	60	198	9.4%	1.06 [0.69 , 1.63]	+	+	+	?	+	?
McLeod 2004	19	67	13	56	5.9%	1.31 [0.58 , 2.96]	+	?	+	?	?	?
Panjari 1999	40	339	38	393	9.0%	1.25 [0.78 , 2.00]	+	?	+	?	+	?
Patten 2020	27	188	29	164	8.0%	0.78 [0.44 , 1.38]	+	?	+	?	?	?
Pollak 2020	9	154	9	160	5.0%	1.04 [0.40 , 2.70]	+	+	+	+	+	+
Price 1991	4	46	1	24	1.3%	2.19 [0.23 , 20.77]	+	+	+	?	?	?
Stotts 2002	20	47	29	86	6.6%	1.46 [0.70 , 3.02]	+	−	?	?	+	−
Tsoh 2010	6	23	2	19	2.1%	3.00 [0.53 , 17.02]	+	+	+	?	+	?
Walsh 1997	17	127	7	125	5.2%	2.61 [1.04 , 6.52]	+	+	+	+	+	+
**Subtotal (95% CI)**		**1769**		**1611**	**70.6%**	**1.17 [0.97 , 1.40]**						
Total events:	364		281									
Heterogeneity: Tau² = 0.00; Chi² = 9.91, df = 10 (P = 0.45); I² = 0%												
Test for overall effect: Z = 1.64 (P = 0.10)												
**5.2.2 Web-based**												
Coleman 2021	26	501	23	501	7.9%	1.14 [0.64 , 2.02]	+	+	+	+	+	+
Marin-Gomez 2019	12	21	3	21	2.7%	8.00 [1.79 , 35.74]	?	?	−	+	+	−
Tsoh 2010	6	23	2	19	2.1%	3.00 [0.53 , 17.02]	+	+	+	?	+	?
**Subtotal (95% CI)**		**545**		**541**	**12.7%**	**2.59 [0.73 , 9.21]**						
Total events:	44		28									
Heterogeneity: Tau² = 0.85; Chi² = 6.28, df = 2 (P = 0.04); I² = 68%												
Test for overall effect: Z = 1.47 (P = 0.14)												
**5.2.3 Smartphone applications / Gamification**												
de Vries 2006	27	141	42	177	8.2%	0.76 [0.44 , 1.31]	+	?	+	?	?	?
Higgins 2022	30	81	9	88	5.8%	5.16 [2.27 , 11.77]	+	?	+	?	+	?
Marin-Gomez 2019	12	21	3	21	2.7%	8.00 [1.79 , 35.74]	?	?	−	+	+	−
**Subtotal (95% CI)**		**243**		**286**	**16.7%**	**2.90 [0.60 , 13.96]**						
Total events:	69		54									
Heterogeneity: Tau² = 1.68; Chi² = 19.37, df = 2 (P < 0.0001); I² = 90%												
Test for overall effect: Z = 1.32 (P = 0.19)												
**Total (95% CI)**		**2557**		**2438**	**100.0%**	**1.43 [1.08 , 1.88]**						
Total events:	477		363									
Heterogeneity: Tau² = 0.16; Chi² = 37.94, df = 16 (P = 0.002); I² = 58%												
Test for overall effect: Z = 2.52 (P = 0.01)												
Test for subgroup differences: Chi² = 2.71, df = 2 (P = 0.26), I² = 26.1%												

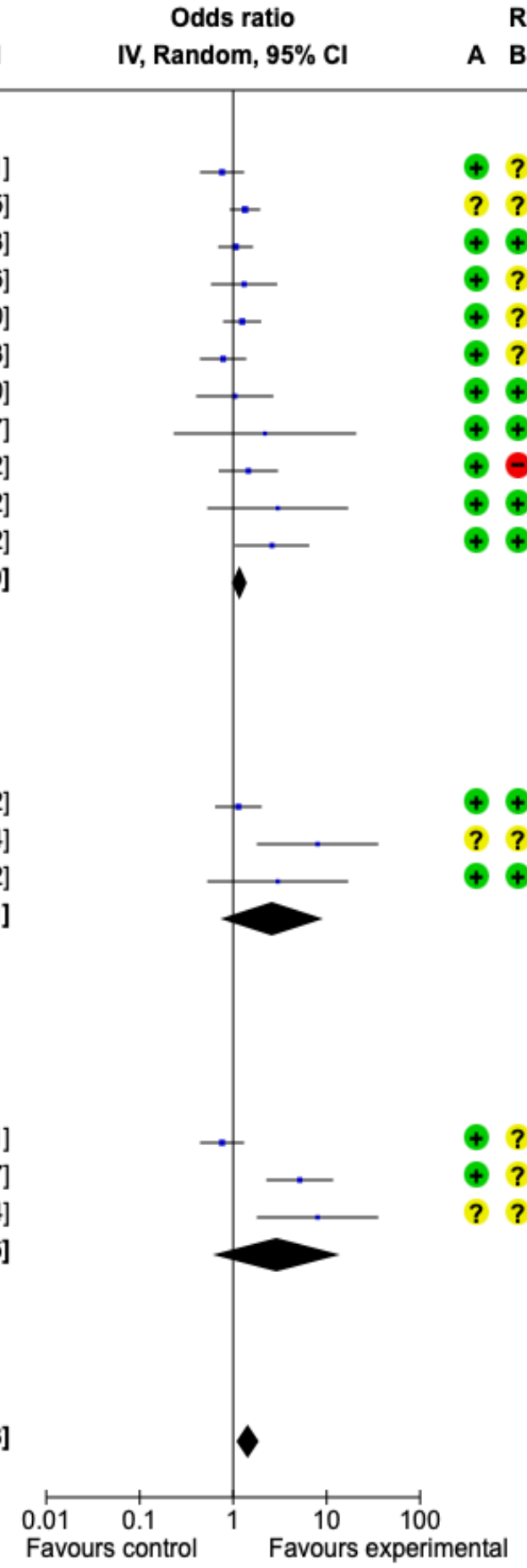

Risk of bias legend
(A) Bias arising from the randomization process
(B) Bias due to deviations from intended interventions
(C) Bias due to missing outcome data
(D) Bias in measurement of the outcome
(E) Bias in selection of the reported result
(F) Overall bias

Fig. 11. Forest plot of the meta-analysis of random effects on smoking cessation of studies including vulnerability approaches

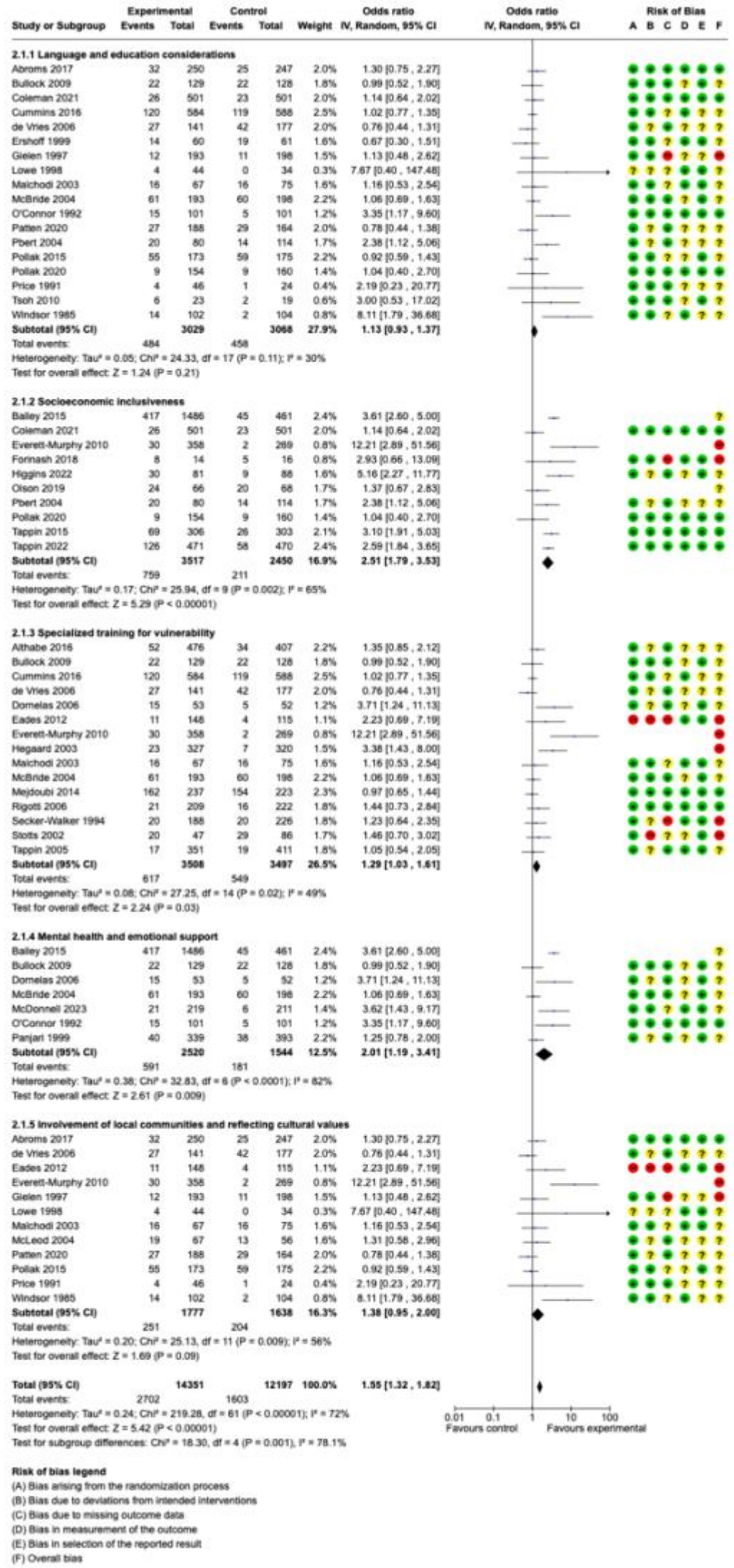

Fig. 12. Forest plot of the meta-analysis of random effects on smoking cessation of studies not including any vulnerability approaches

Study or Subgroup	Experimental Events	Experimental Total	Control Events	Control Total	Weight	Odds ratio IV, Random, 95% CI	Risk of Bias A	B	C	D	E	F
**6.2.1 None**												
Albrecht 1998	3	10	5	10	2.0%	0.43 [0.07 , 2.68]	?	?	−	?	+	−
Britton 2006	19	66	21	69	7.5%	0.92 [0.44 , 1.94]						−
Cope 2003	22	109	4	83	4.5%	4.99 [1.65 , 15.12]	?	+	−	?	+	−
Hajek 2001	17	545	17	575	8.1%	1.06 [0.53 , 2.09]	+	?	?	+	+	?
Hjalmarson 1991	134	444	51	209	12.1%	1.34 [0.92 , 1.95]	?	?	+	+	+	?
Jaakkola 2001	58	306	22	152	9.9%	1.38 [0.81 , 2.36]						−
Keten 2013	19	41	18	43	6.3%	1.20 [0.51 , 2.84]	+	+	+	?	?	?
Loukopoulou 2018	19	42	9	42	5.5%	3.03 [1.17 , 7.87]	+	+	+	+	+	+
Marin-Gomez 2019	12	21	3	21	2.9%	8.00 [1.79 , 35.74]	?	?	−	+	+	−
McBride 2005	41	185	30	195	10.1%	1.57 [0.93 , 2.64]	?	?	?	−	+	−
Moore 2002	113	600	144	695	13.4%	0.89 [0.67 , 1.17]	+	?	+	?	+	?
Polanska 2004	126	261	60	181	11.8%	1.88 [1.27 , 2.79]	?	+	+	?	?	?
Walsh 1997	17	127	7	125	5.8%	2.61 [1.04 , 6.52]	+	+	+	+	+	+
**Subtotal (95% CI)**		**2757**		**2400**	**100.0%**	**1.51 [1.14 , 2.00]**						
Total events:	600		391									
Heterogeneity: Tau² = 0.13; Chi² = 30.48, df = 12 (P = 0.002); I² = 61%												
Test for overall effect: Z = 2.89 (P = 0.004)												
**Total (95% CI)**		**2757**		**2400**	**100.0%**	**1.51 [1.14 , 2.00]**						
Total events:	600		391									
Heterogeneity: Tau² = 0.13; Chi² = 30.48, df = 12 (P = 0.002); I² = 61%												
Test for overall effect: Z = 2.89 (P = 0.004)												
Test for subgroup differences: Not applicable												

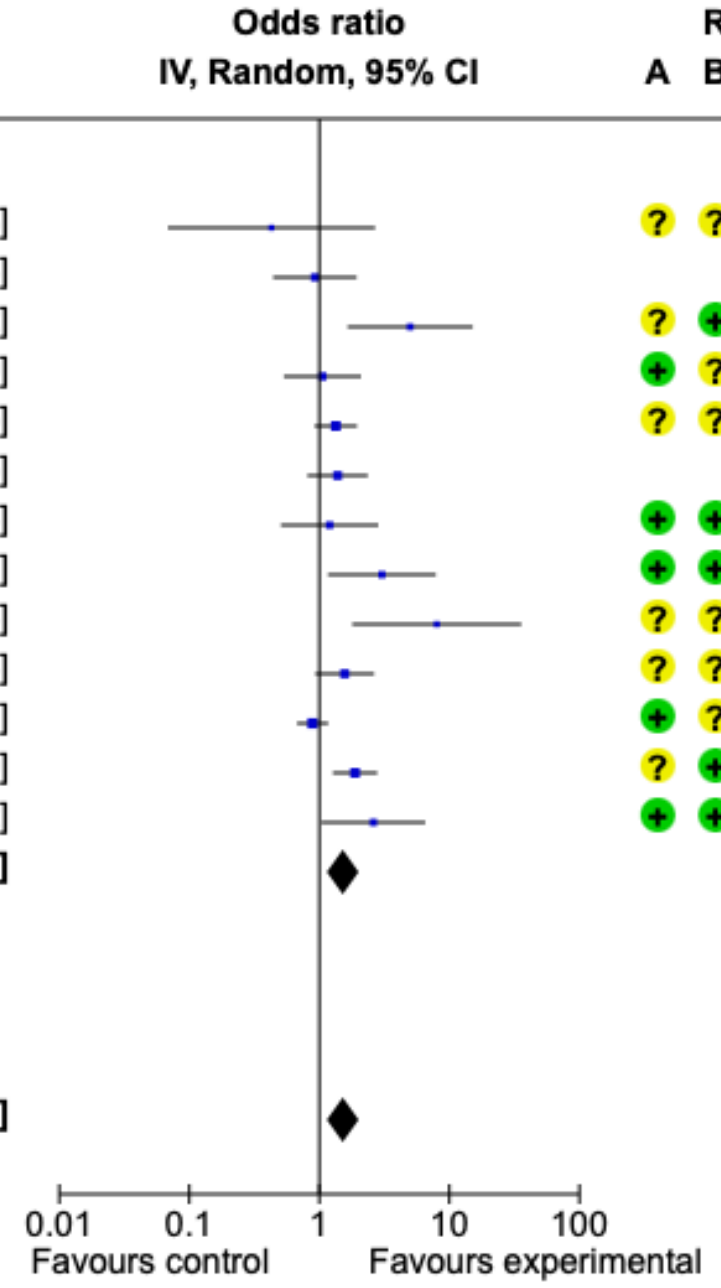

Risk of bias legend
(A) Bias arising from the randomization process
(B) Bias due to deviations from intended interventions
(C) Bias due to missing outcome data
(D) Bias in measurement of the outcome
(E) Bias in selection of the reported result
(F) Overall bias

Fig. 13. Forest plot of the meta-analysis of random effects on smoking cessation of studies involving the father in either an active or passive role

Study or Subgroup	Experimental Events	Experimental Total	Control Events	Control Total	Weight	Odds ratio IV, Random, 95% CI	A	B	C	D	E	F
**7.1.1 Active**												
de Vries 2006	27	141	42	177	7.6%	0.76 [0.44 , 1.31]	+	?	+	?	?	?
Eades 2012	11	148	4	115	2.3%	2.23 [0.69 , 7.19]	−	−	−	+	+	−
Everett-Murphy 2010	30	358	2	269	1.6%	12.21 [2.89 , 51.56]						−
Jaakkola 2001	58	306	22	152	7.8%	1.38 [0.81 , 2.36]						−
McBride 2004	61	193	60	198	10.1%	1.06 [0.69 , 1.63]	+	+	+	?	+	?
McBride 2005	41	195	30	195	8.1%	1.46 [0.87 , 2.46]	?	?	?	−	+	−
Moore 2002	113	600	144	695	14.7%	0.89 [0.67 , 1.17]	+	?	+	?	+	?
Pollak 2015	55	173	59	175	9.6%	0.92 [0.59 , 1.43]	+	+	?	?	+	?
Stotts 2002	20	47	29	86	5.0%	1.46 [0.70 , 3.02]	+	−	?	?	+	−
**Subtotal (95% CI)**		**2161**		**2062**	**66.8%**	**1.20 [0.90 , 1.59]**						
Total events:	416		392									
Heterogeneity: Tau² = 0.10; Chi² = 19.18, df = 8 (P = 0.01); I² = 58%												
Test for overall effect: Z = 1.26 (P = 0.21)												
**7.1.2 Passive**												
Britton 2006	19	66	21	69	4.9%	0.92 [0.44 , 1.94]						−
Coleman 2021	26	501	23	501	7.1%	1.14 [0.64 , 2.02]	+	+	+	+	+	+
Hajek 2001	17	545	17	575	5.6%	1.06 [0.53 , 2.09]	+	?	?	+	+	?
Lowe 1998	4	44	0	34	0.4%	7.67 [0.40 , 147.48]	?	?	?	+	+	?
McLeod 2004	19	67	13	56	4.2%	1.31 [0.58 , 2.96]	+	?	+	?	?	?
Mejdoubi 2014	162	237	154	223	11.0%	0.97 [0.65 , 1.44]	+	+	+	+	+	+
**Subtotal (95% CI)**		**1460**		**1458**	**33.2%**	**1.05 [0.81 , 1.36]**						
Total events:	247		228									
Heterogeneity: Tau² = 0.00; Chi² = 2.37, df = 5 (P = 0.80); I² = 0%												
Test for overall effect: Z = 0.40 (P = 0.69)												
**Total (95% CI)**		**3621**		**3520**	**100.0%**	**1.12 [0.93 , 1.35]**						
Total events:	663		620									
Heterogeneity: Tau² = 0.04; Chi² = 21.55, df = 14 (P = 0.09); I² = 35%												
Test for overall effect: Z = 1.22 (P = 0.22)												
Test for subgroup differences: Chi² = 0.44, df = 1 (P = 0.51), I² = 0%												

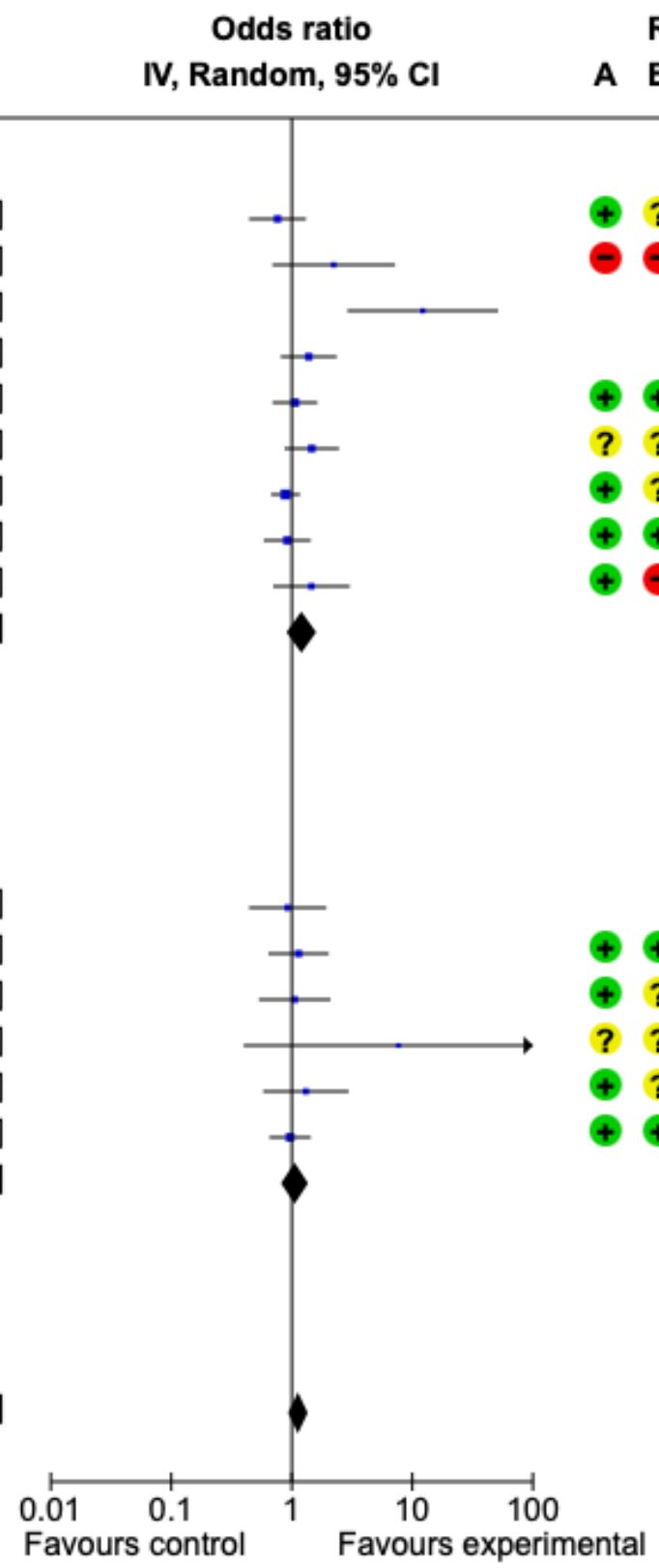

Risk of bias legend
(A) Bias arising from the randomization process
(B) Bias due to deviations from intended interventions
(C) Bias due to missing outcome data
(D) Bias in measurement of the outcome
(E) Bias in selection of the reported result
(F) Overall bias

Fig. 14. Forest plot of the meta-analysis of random effects on smoking cessation of studies not involving the father in any active or passive role

Study or Subgroup	Experimental Events	Experimental Total	Control Events	Control Total	Weight	Odds ratio IV, Random, 95% CI
**7.2.1 No involvement**						
Abroms 2017	32	250	25	247	3.7%	1.30 [0.75 , 2.27]
Albrecht 1998	3	10	5	10	1.0%	0.43 [0.07 , 2.68]
Althabe 2016	52	476	34	407	4.1%	1.35 [0.85 , 2.12]
Bailey 2015	417	1486	45	461	4.5%	3.61 [2.60 , 5.00]
Bullock 2009	22	129	22	128	3.3%	0.99 [0.52 , 1.90]
Cope 2003	22	109	4	83	2.0%	4.99 [1.65 , 15.12]
Cummins 2016	120	584	119	588	4.6%	1.02 [0.77 , 1.35]
Dornelas 2006	15	53	5	52	2.0%	3.71 [1.24 , 11.13]
Ershoff 1999	14	60	19	61	2.8%	0.67 [0.30 , 1.51]
Forinash 2018	8	14	5	16	1.4%	2.93 [0.66 , 13.09]
Gielen 1997	12	193	11	198	2.7%	1.13 [0.48 , 2.62]
Hegaard 2003	23	327	7	320	2.7%	3.38 [1.43 , 8.00]
Higgins 2022	30	81	9	88	2.8%	5.16 [2.27 , 11.77]
Hjalmarson 1991	134	444	51	209	4.3%	1.34 [0.92 , 1.95]
Keten 2013	29	41	18	43	2.5%	3.36 [1.36 , 8.30]
Loukopoulou 2018	19	42	9	42	2.4%	3.03 [1.17 , 7.87]
Malchodi 2003	16	67	16	75	2.9%	1.16 [0.53 , 2.54]
Marin-Gomez 2019	12	21	3	21	1.4%	8.00 [1.79 , 35.74]
McDonnell 2023	21	219	6	211	2.5%	3.62 [1.43 , 9.17]
O'Connor 1992	15	101	5	101	2.2%	3.35 [1.17 , 9.60]
Olson 2019	24	66	20	68	3.1%	1.37 [0.67 , 2.83]
Panjari 1999	40	339	38	393	4.0%	1.25 [0.78 , 2.00]
Patten 2020	27	188	29	164	3.6%	0.78 [0.44 , 1.38]
Pbert 2004	20	80	14	114	3.0%	2.38 [1.12 , 5.06]
Polanska 2004	126	261	60	181	4.3%	1.88 [1.27 , 2.79]
Pollak 2020	9	154	9	160	2.4%	1.04 [0.40 , 2.70]
Price 1991	4	46	1	24	0.7%	2.19 [0.23 , 20.77]
Rigotti 2006	21	209	16	222	3.2%	1.44 [0.73 , 2.84]
Secker-Walker 1994	20	188	20	226	3.3%	1.23 [0.64 , 2.35]
Tappin 2005	17	351	19	411	3.3%	1.05 [0.54 , 2.05]
Tappin 2015	69	306	26	303	3.9%	3.10 [1.91 , 5.03]
Tappin 2022	126	471	58	470	4.4%	2.59 [1.84 , 3.65]
Tsoh 2010	6	23	2	19	1.1%	3.00 [0.53 , 17.02]
Walsh 1997	17	127	7	125	2.5%	2.61 [1.04 , 6.52]
Windsor 1985	14	102	2	104	1.3%	8.11 [1.79 , 36.68]
**Subtotal (95% CI)**		**7618**		**6345**	**100.0%**	**1.85 [1.51 , 2.27]**
Total events:	1556		739			
**Total (95% CI)**		**7618**		**6345**	**100.0%**	**1.85 [1.51 , 2.27]**
Total events:	1556		739			

Subtotal: Heterogeneity: $Tau^2 = 0.21$; $Chi^2 = 106.47$, df = 34 ($P < 0.00001$); $I^2 = 68\%$
Test for overall effect: $Z = 5.93$ ($P < 0.00001$)

Total: Heterogeneity: $Tau^2 = 0.21$; $Chi^2 = 106.47$, df = 34 ($P < 0.00001$); $I^2 = 68\%$
Test for overall effect: $Z = 5.93$ ($P < 0.00001$)
Test for subgroup differences: Not applicable

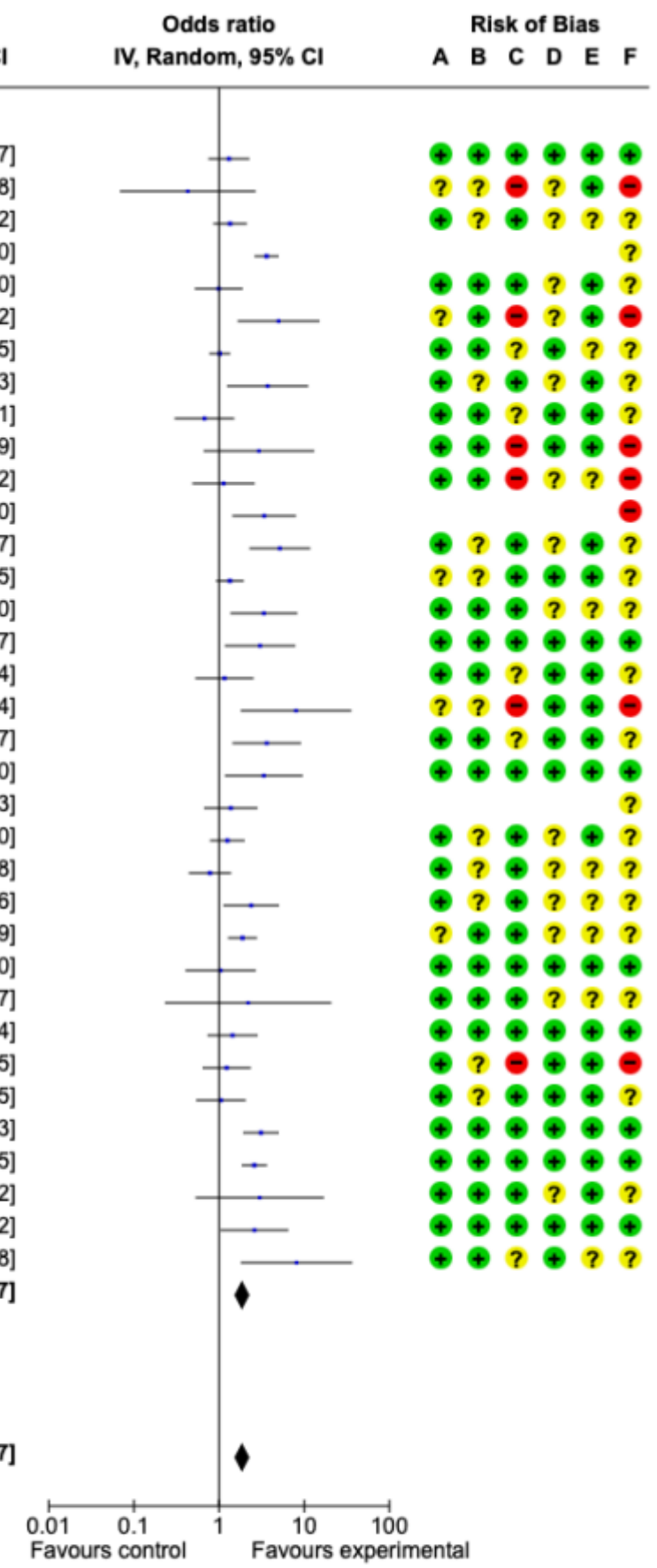

Risk of bias legend
(A) Bias arising from the randomization process
(B) Bias due to deviations from intended interventions
(C) Bias due to missing outcome data
(D) Bias in measurement of the outcome
(E) Bias in selection of the reported result
(F) Overall bias

Fig. 15. Forest plot of the meta-analysis of random effects on partner's smoking cessation

Study or Subgroup	Experimental Events	Experimental Total	Control Events	Control Total	Weight	Odds ratio IV, Random, 95% CI	Risk of Bias A	B	C	D	E	F
Aveyard 2005	24	465	15	465	16.0%	1.63 [0.85 , 3.15]	+	?	+	?	?	?
Luk 2021	36	527	19	526	21.4%	1.96 [1.11 , 3.46]	+	+	+	+	+	+
Stanton 2004	48	291	25	270	26.2%	1.94 [1.16 , 3.24]	+	+	+	+	+	+
Xia 2020	75	333	34	368	36.4%	2.86 [1.85 , 4.42]	+	+	+	+	+	+
**Total (95% CI)**		**1616**		**1629**	**100.0%**	**2.18 [1.67 , 2.83]**						
Total events:	183		93									

Heterogeneity: Tau² = 0.00; Chi² = 2.55, df = 3 (P = 0.47); I² = 0%

Test for overall effect: Z = 5.78 (P < 0.00001)

Test for subgroup differences: Not applicable

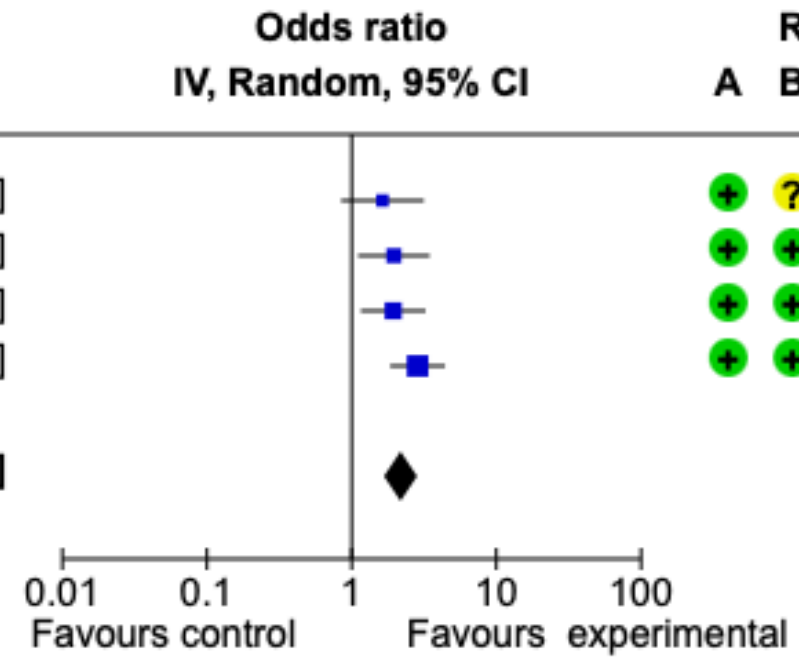

Risk of bias legend

(A) Bias arising from the randomization process

(B) Bias due to deviations from intended interventions

(C) Bias due to missing outcome data

(D) Bias in measurement of the outcome

(E) Bias in selection of the reported result

(F) Overall bias

II. FUNNEL PLOTS OF RESULTS

Fig 1. Interventions including vulnerability approaches

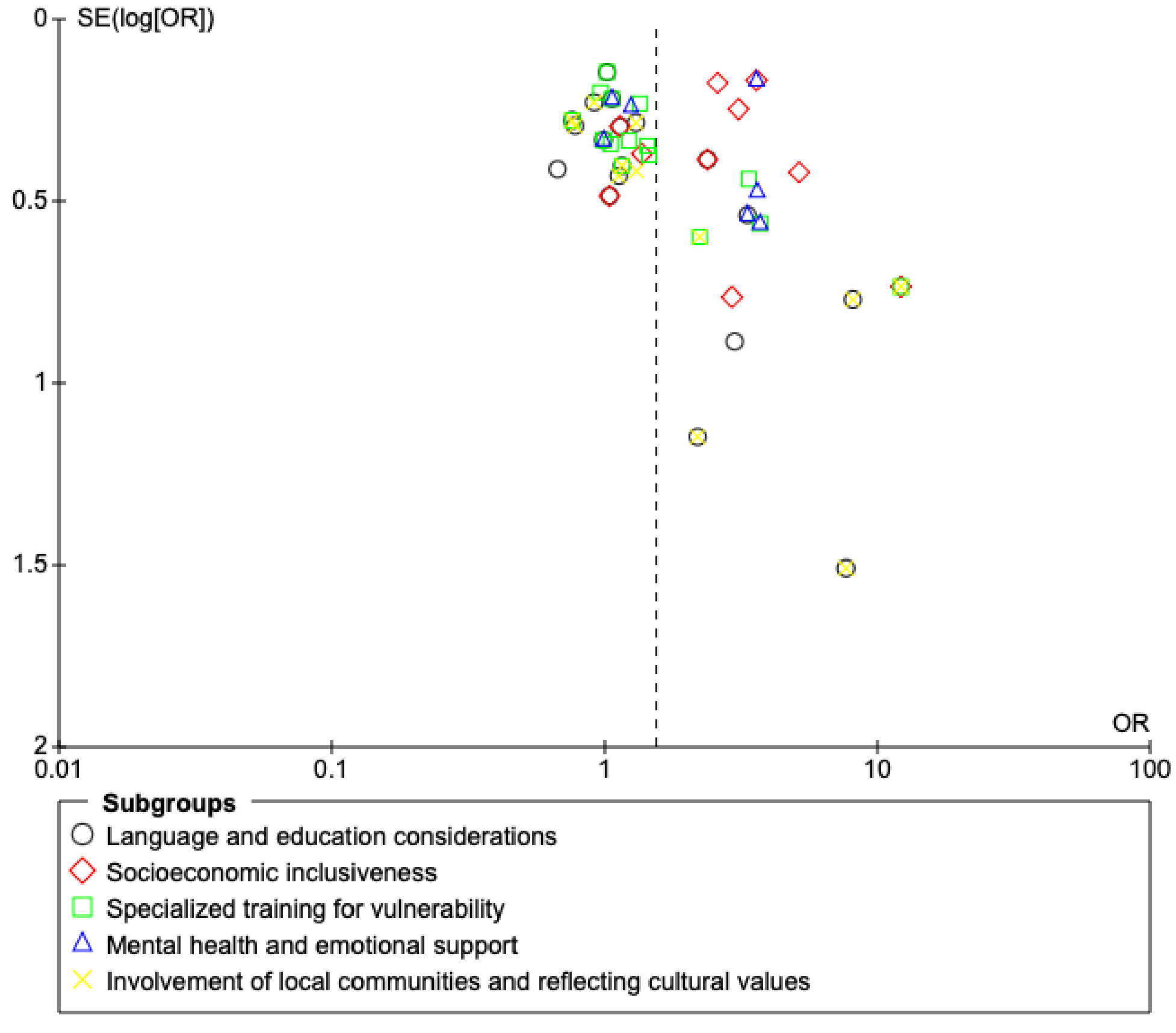

Fig 2. Interventions not including vulnerability approaches

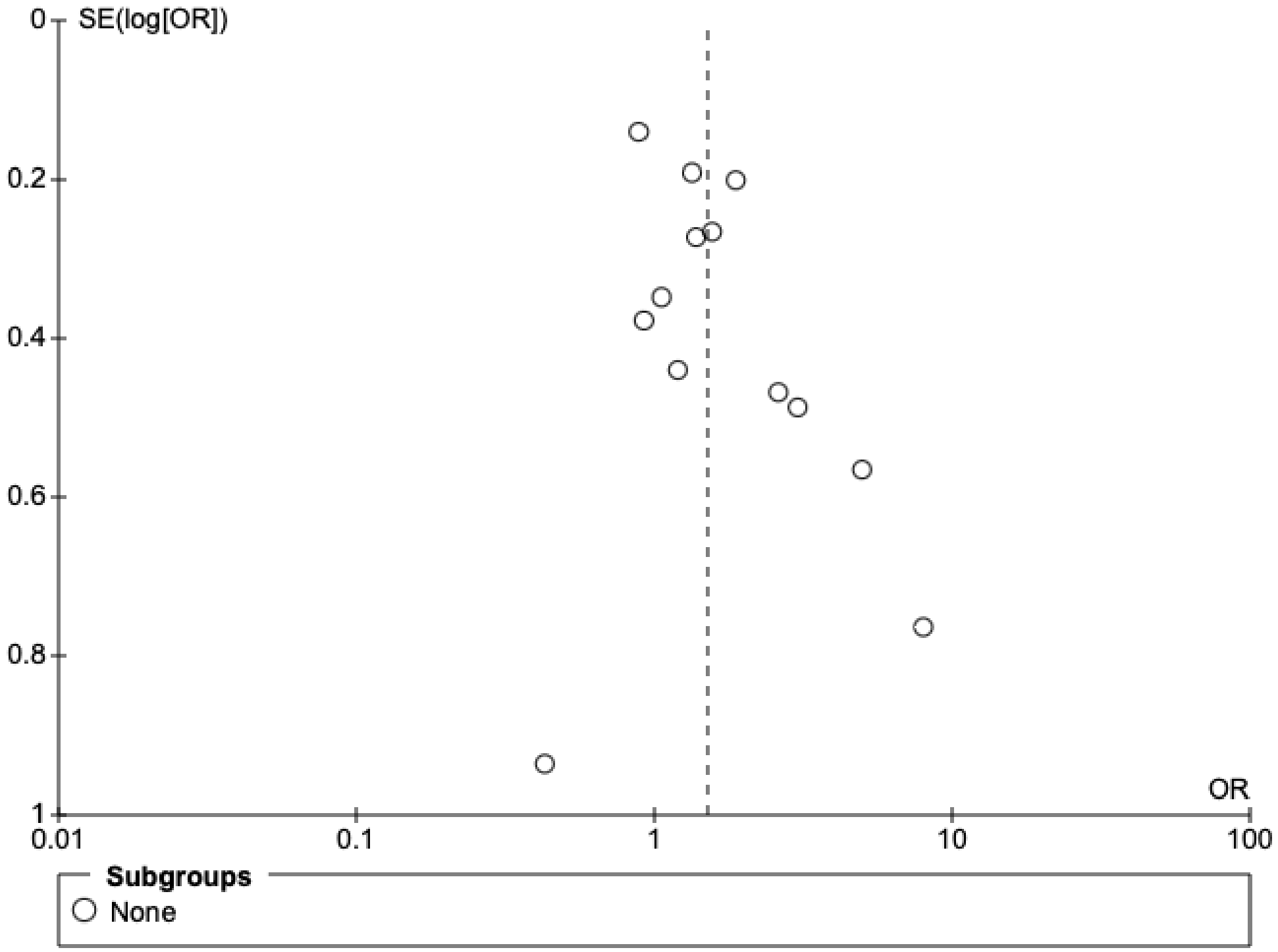

Fig 3. Interventions including father involvement

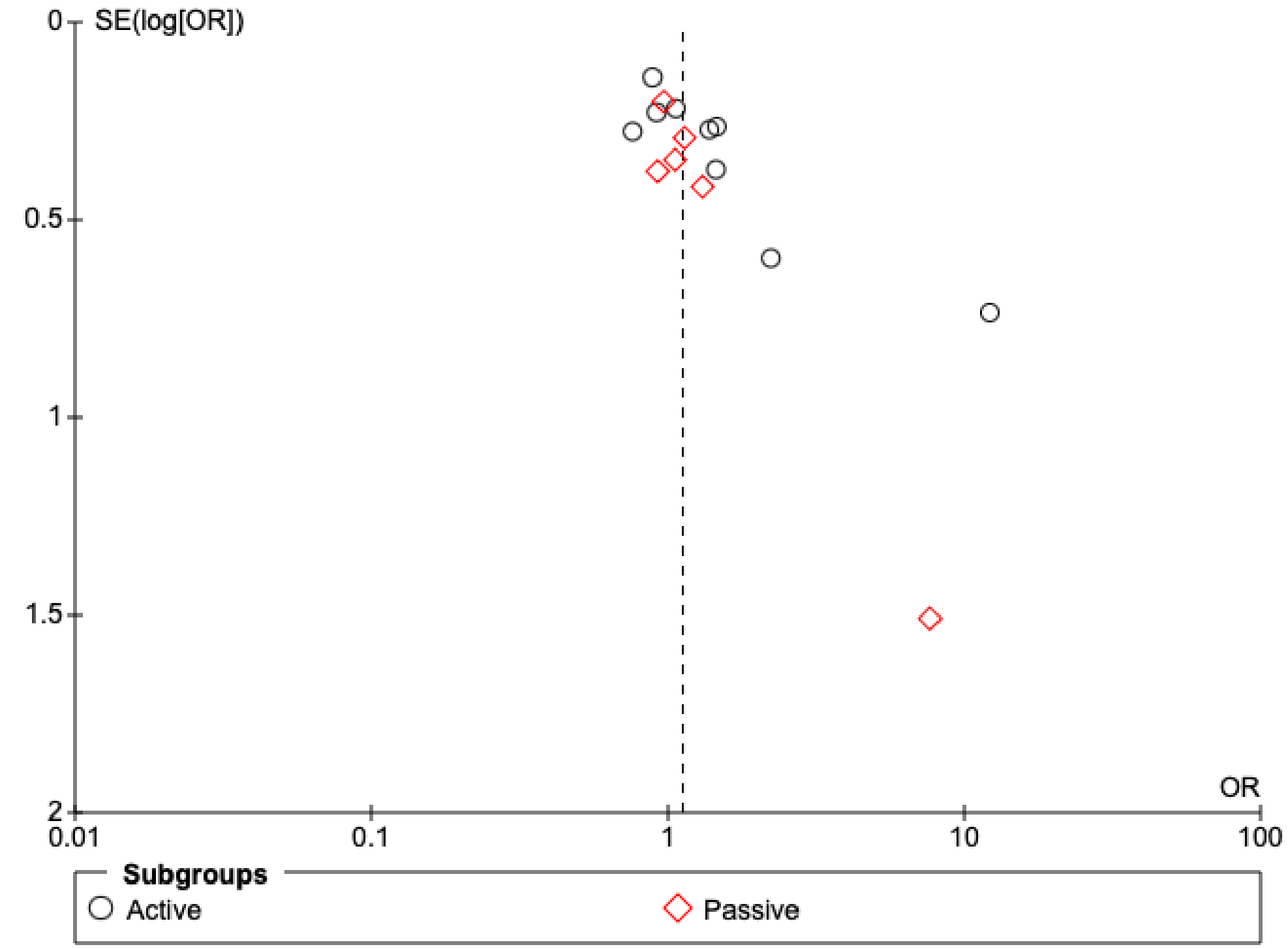

Fig 4. Interventions not including father involvement

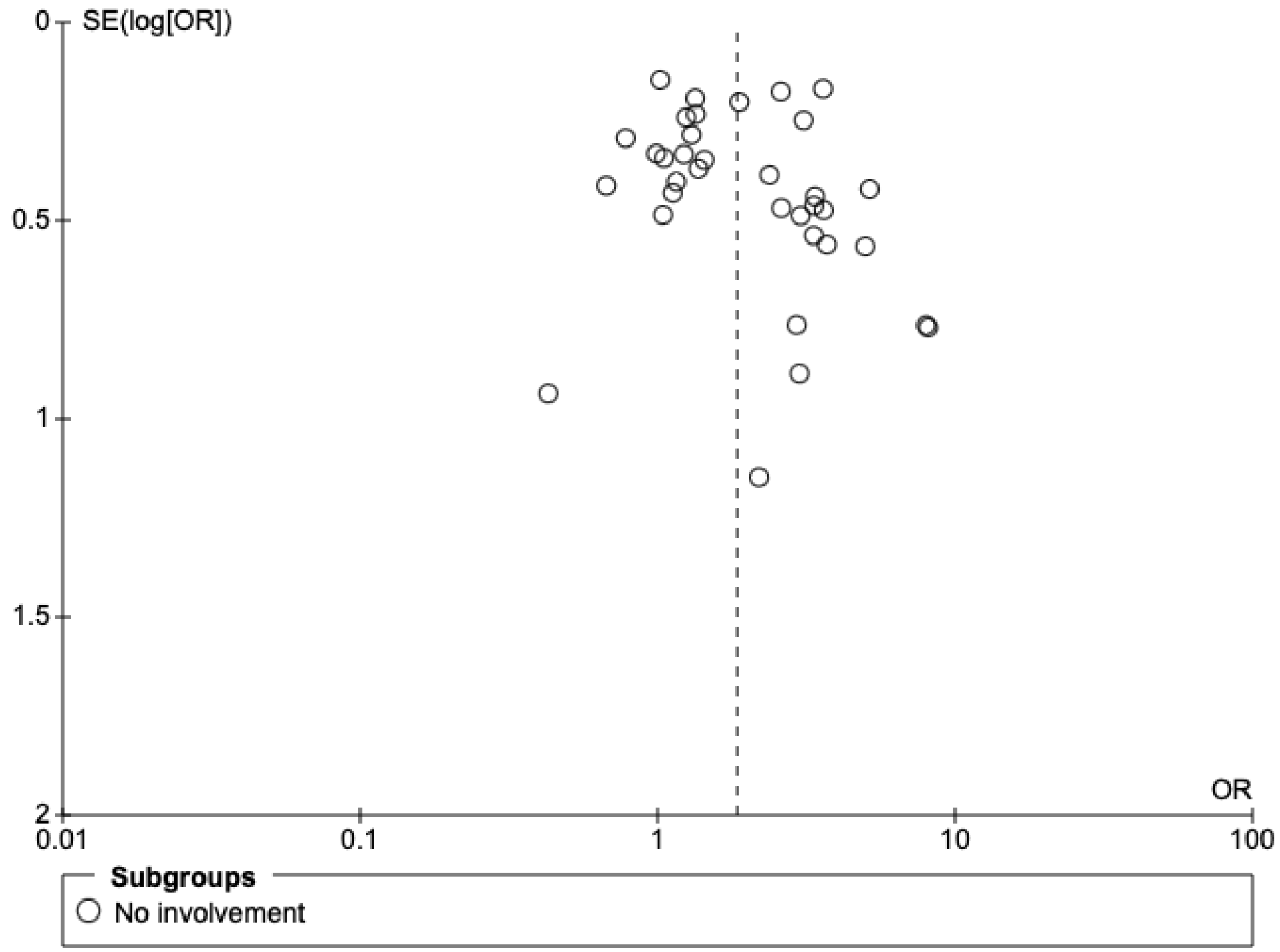

Fig 5. Interventions on paternal smoking cessation outcome

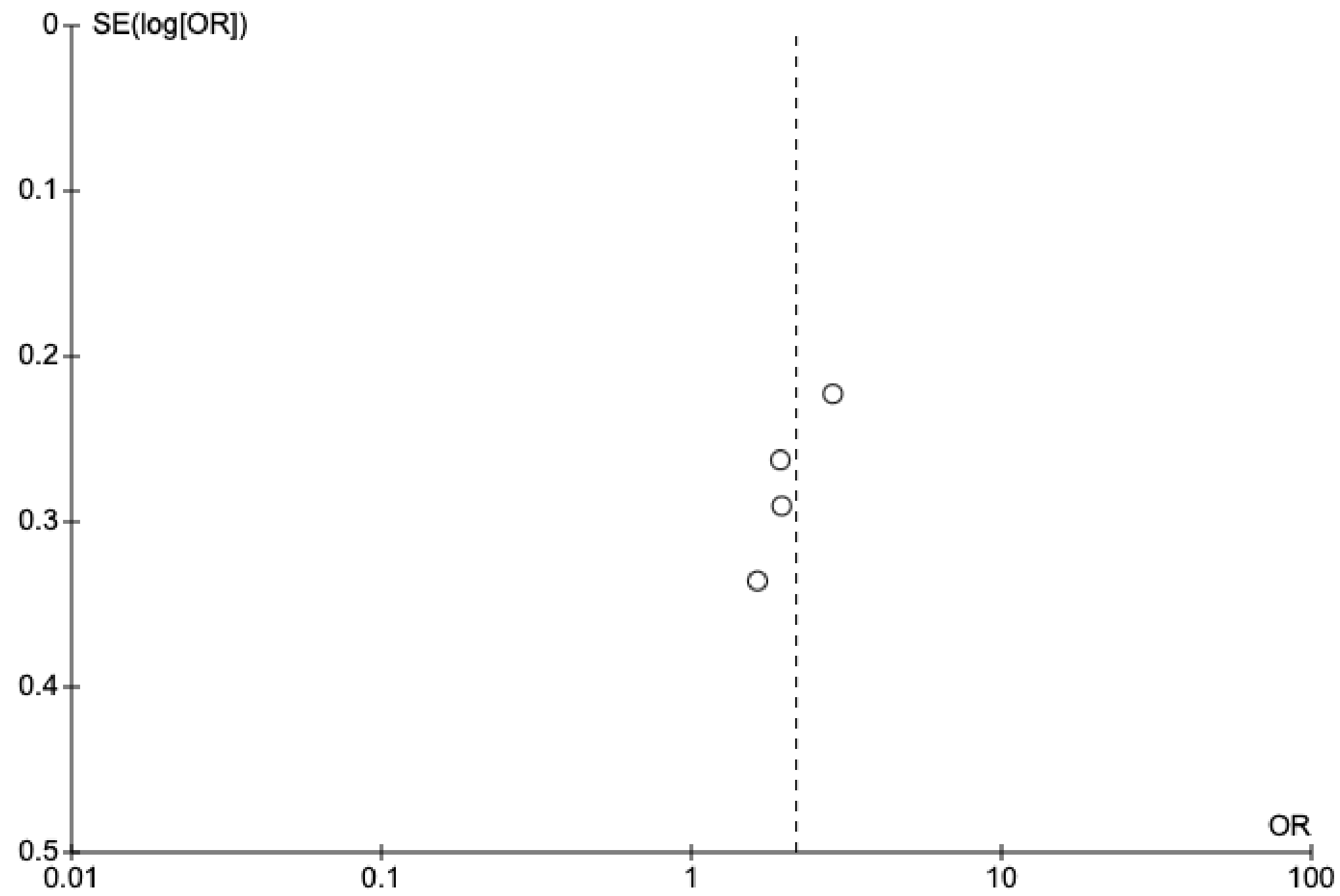

III. EGGER'S TEST RESULTS FOR PUBLICATION BIAS

Two-tailed α = 0.10 (conventional threshold for meta-analytic publication bias assessment). Test not applicable for k < 10 studies.

Analysis	k (studies)	Egger Intercept	SE (Intercept)	t Statistic	p-value (two-tailed)	Significant (α = 0.10)?	Interpretation
Overall maternal (all studies)	50	0,0465	0,1627	0,286	0,7762	No	No evidence of small-study effects
6.2.1 No vulnerability approach	13	-0,1839	0,236	-0,779	0,4522	No	No evidence of small-study effects
2.1.1 Language & education	19	-0,4042	0,1727	-2,34	0,0317	Yes	Negative intercept – smaller studies show smaller effects (possible heterogeneity artifact)
2.1.2 Socioeconomic inclusiveness	10	1,0809	0,3622	2,984	0,0175	Yes	Positive intercept – smaller studies show larger effects (classic publication bias)
2.1.3 Specialised provider training	15	-0,4801	0,1826	-2,63	0,0208	Yes	Negative intercept – smaller studies show smaller effects (possible heterogeneity artifact)
2.1.4 Mental health & emotional support	7	0,7017	0,6531	1,074	0,3317	n/a (<10 studies)	
2.1.5 Community involvement	12	-0,7598	0,2482	-3,061	0,012	Yes	Negative intercept – smaller studies show smaller effects (possible heterogeneity artifact)
7.2.1 No father involvement	35	0,3614	0,2034	1,777	0,0848	Yes	Positive intercept – smaller studies show larger effects (classic publication bias)
7.1.1 Active father involvement	9	-0,5993	0,2014	-2,975	0,0207	n/a (<10 studies)	
7.1.2 Passive father involvement	6	-0,3305	0,1269	-2,604	0,0598	n/a (<10 studies)	

Notes: Egger's test regresses log(OR) on SE(log OR) with inverse-variance weights. A significant intercept ($p < 0.10$) suggests small-study effects or publication bias. For subgroups with significant heterogeneity ($I^2 > 60\%$), a significant Egger's test may reflect heterogeneity rather than true publication bias. 0.5 continuity correction applied to studies with zero-event cells.

8. GRADE ASSESSMENT

Fig 1. Vulnerability approaches compared to no approach for maternal smoking cessation

Summary of findings:

Vulnerability approaches compared to no approach for maternal smoking cessation

Patient or population: maternal smoking cessation
Setting:
Intervention: Vulnerability approaches
Comparison: no approach

Outcomes	Anticipated absolute effects* (95% CI): Risk with no approach	Anticipated absolute effects* (95% CI): Risk with Vulnerability approaches	Relative effect (95% CI)	№ of participants (studies)	Certainty of the evidence (GRADE)	Comments
Language and education considerations assessed with: Maternal smoking cessation	15 per 100	**17 per 100** (14 to 19)	**OR 1.13** (0.93 to 1.37)	6097 (18 RCTs)	⊕⊕◯◯ Low[a,b,c]	Language and education considerations likely results in little to no difference in increasing maternal smoking cessation.
Socioeconomic inclusiveness assessed with: Maternal smoking cessation	9 per 100	**19 per 100** (14 to 25)	**OR 2.51** (1.79 to 3.53)	5967 (10 RCTs)	⊕⊕◯◯ Low[d,e,f]	The evidence suggests that socioeconomic inclusiveness results in a slight increase in maternal smoking cessation.
Specialized training for vulnerability assessed with: Maternal smoking cessation	16 per 100	**19 per 100** (16 to 23)	**OR 1.29** (1.03 to 1.61)	7005 (15 RCTs)	⊕⊕◯◯ Low[a,b]	Specialized training for vulnerability may result in a slight increase in maternal smoking cessation.
Mental health and emotional support assessed with: Maternal smoking cessation	12 per 100	**21 per 100** (14 to 31)	**OR 2.01** (1.19 to 3.41)	4064 (7 RCTs)	⊕◯◯◯ Very low[a,f,g]	Mental health and emotional support may increase maternal smoking cessation but the evidence is very uncertain.
Involvement of local communities and reflecting cultural values assessed with: Maternal smoking cessation	12 per 100	**16 per 100** (12 to 22)	**OR 1.38** (0.95 to 2.00)	3415 (12 RCTs)	⊕◯◯◯ Very low[a,e,h]	Involvement of local communities and reflecting cultural values may increase maternal smoking cessation but the evidence is very uncertain.
All vulnerability approaches assessed with: Maternal smoking cessation	13 per 100	**19 per 100** (17 to 22)	**OR 1.55** (1.32 to 1.82)	26548 (37 RCTs)	⊕◯◯◯ Very low[c,d,g]	The evidence suggests that vulnerability approaches slightly increase maternal smoking cessation.
No vulnerability approach - total	16 per 100	**23 per 100** (18 to 28)	**OR 1.51** (1.14 to 2.00)	5157 (13 RCTs)	⊕◯◯◯ Very low[a,e,h]	The evidence is very uncertain about the effect of no vulnerability approach in increasing maternal smoking cessation.

***The risk in the intervention group** (and its 95% confidence interval) is based on the assumed risk in the comparison group and the **relative effect** of the intervention (and its 95% CI).

CI: confidence interval; **OR:** odds ratio

GRADE Working Group grades of evidence
High certainty: we are very confident that the true effect lies close to that of the estimate of the effect.
Moderate certainty: we are moderately confident in the effect estimate: the true effect is likely to be close to the estimate of the effect, but there is a possibility that it is substantially different.
Low certainty: our confidence in the effect estimate is limited: the true effect may be substantially different from the estimate of the effect.
Very low certainty: we have very little confidence in the effect estimate: the true effect is likely to be substantially different from the estimate of effect.

Explanations

a. Under 25% of studies presenting low risk of bias
b. Low heterogeneity among studies (0-50%)
c. Narrow confidence interval
d. Under 50% of studies presenting low risk of bias
e. Moderate heterogeneity among studies (51-69%)
f. Wide confidence interval likely due to heterogeneity
g. High level of heterogeneity (>70%)
h. Moderate range in confidence interval

Fig 2. Father involvement compared to no involvement for maternal smoking cessation

Summary of findings:

Father involvement compared to no involvement for maternal smoking cessation

Patient or population: maternal smoking cessation
Setting:
Intervention: Father involvement
Comparison: no involvement

Outcomes	Anticipated absolute effects* (95% CI)		Relative effect (95% CI)	№ of participants (studies)	Certainty of the evidence (GRADE)	Comments
	**Risk with no involvement**	**Risk with Father involvement**				
Active involvement assessed with: Maternal smoking cessation	19 per 100	**22 per 100** (17 to 27)	**OR 1.20** (0.90 to 1.59)	4223 (9 RCTs)	⊕◯◯◯ Very low[a,b,c]	Father's active involvement may increase maternal smoking cessation but the evidence is very uncertain.
Passive involvement assessed with: Maternal smoking cessation	16 per 100	**16 per 100** (13 to 20)	**OR 1.05** (0.81 to 1.36)	2918 (6 RCTs)	⊕⊕⊕◯ Moderate[c,d,e]	Father's passive involvement likely results in little to no difference in increasing maternal smoking cessation.
Father Involvement assessed with: Maternal smoking cessation	18 per 100	**19 per 100** (17 to 22)	**OR 1.12** (0.93 to 1.35)	7141 (15 RCTs)	⊕⊕◯◯ Low[a,c,e]	The evidence suggests that father involvement results in little to no difference in increasing maternal smoking cessation.
No father involvement assessed with: Maternal smoking cessation	12 per 100	**20 per 100** (17 to 23)	**OR 1.85** (1.51 to 2.27)	13963 (35 RCTs)	⊕◯◯◯ Very low[a,b,f]	The evidence is very uncertain about the effect of no father involvement on increasing maternal smoking cessation.

***The risk in the intervention group** (and its 95% confidence interval) is based on the assumed risk in the comparison group and the **relative effect** of the intervention (and its 95% CI).

CI: confidence interval; **OR:** odds ratio

GRADE Working Group grades of evidence
High certainty: we are very confident that the true effect lies close to that of the estimate of the effect.
Moderate certainty: we are moderately confident in the effect estimate: the true effect is likely to be close to the estimate of the effect, but there is a possibility that it is substantially different.
Low certainty: our confidence in the effect estimate is limited: the true effect may be substantially different from the estimate of the effect.
Very low certainty: we have very little confidence in the effect estimate: the true effect is likely to be substantially different from the estimate of effect.

Explanations

a. Under 25% of studies presenting low risk of bias
b. Moderate level of heterogeneity (51-69%)
c. Narrow confidence interval
d. Under 50% of studies presenting low risk of bias
e. Low level of heterogeneity (0-50%)
f. Wide confidence interval likely due to high heterogeneity

Fig 2. Father directed interventions compared to usual care for increasing smoking cessation rates among expectant fathers

Summary of findings:

Father directed interventions compared to usual care for increasing smoking cessation rates among expectant fathers

Patient or population: increasing smoking cessation rates among expectant fathers
Setting:
Intervention: father directed interventions
Comparison: usual care

Outcomes	Anticipated absolute effects* (95% CI)		Relative effect (95% CI)	№ of participants (studies)	Certainty of the evidence (GRADE)	Comments
	**Risk with usual care**	**Risk with father directed interventions**				
Father's involvement assessed with: Paternal smoking cessation	6 per 100	**12 per 100** (9 to 15)	**OR 2.18** (1.67 to 2.83)	3245 (4 RCTs)	⊕⊕⊕⊕ High[a,b,c]	Father directed interventions result in increased paternal smoking cessation.

***The risk in the intervention group** (and its 95% confidence interval) is based on the assumed risk in the comparison group and the **relative effect** of the intervention (and its 95% CI).

CI: confidence interval; **OR:** odds ratio

GRADE Working Group grades of evidence
High certainty: we are very confident that the true effect lies close to that of the estimate of the effect.
Moderate certainty: we are moderately confident in the effect estimate: the true effect is likely to be close to the estimate of the effect, but there is a possibility that it is substantially different.
Low certainty: our confidence in the effect estimate is limited: the true effect may be substantially different from the estimate of the effect.
Very low certainty: we have very little confidence in the effect estimate: the true effect is likely to be substantially different from the estimate of effect.

Explanations

a. Over 75% of studies presenting low risk of bias
b. Low heterogeneity among studies (<50%)
c. Narrow confidence interval
